# Supplementary material for: Advanced multi-modal mass spectrometry imaging reveals functional differences of placental villous compartments at microscale resolution
Source: Nat Commun. 2025 Feb 28;16:2061. doi: 10.1038/s41467-025-57107-y (PMC11871073; doi:10.1038/s41467-025-57107-y)
Supplement: Supplementary file 1 — Supplementary Information [file 41467_2025_57107_MOESM1_ESM.pdf]

Supplementary Information

Advanced multi-modal mass spectrometry imaging reveals functional differences of placental villous compartments at microscale resolution

Marija Veličković[1], Leena Kadam[2], Joonhoon Kim[3], Kevin J Zemaitis[1], Dušan Veličković[1], Yuqian Gao[4], Ruonan Wu[4], Thomas L Fillmore[1], Daniel Orton[4], Sarah M. Williams[1], Matthew E Monroe[4], Ronald J Moore[4], Paul D Piehowski[1], Lisa M Bramer[4]*, Leslie Myatt[2]*, Kristin E Burnum-Johnson[1*]

[1]The Environmental Molecular Sciences Laboratory, Pacific Northwest National Laboratory, Richland, WA, USA. [2]Department of Obstetrics & Gynecology, Oregon Health & Science University, Portland, OR, USA. [3]Energy and Environment Directorate, Pacific Northwest National Laboratory, Richland, WA, USA. [4]Biological Sciences Division, Pacific Northwest National Laboratory, Richland, WA, USA.

*Corresponding authors:
Kristin E Burnum-Johnson, Kristin.Burnum-Johnson@pnnl.gov
Leslie Myatt, myattl@ohsu.edu
Lisa M Bramer, lisa.bramer@pnnl.gov

Summary:

Supplementary Figure 1 - Page 2

Supplementary Figure 2 - Page 3

Supplementary Figure 3 - Page 62

Supplementary Figure 4 – Page 63

Supplementary Figure 5 – Page 65

Supplementary Statistical Methods – Page 67

Supplementary References – Page 77

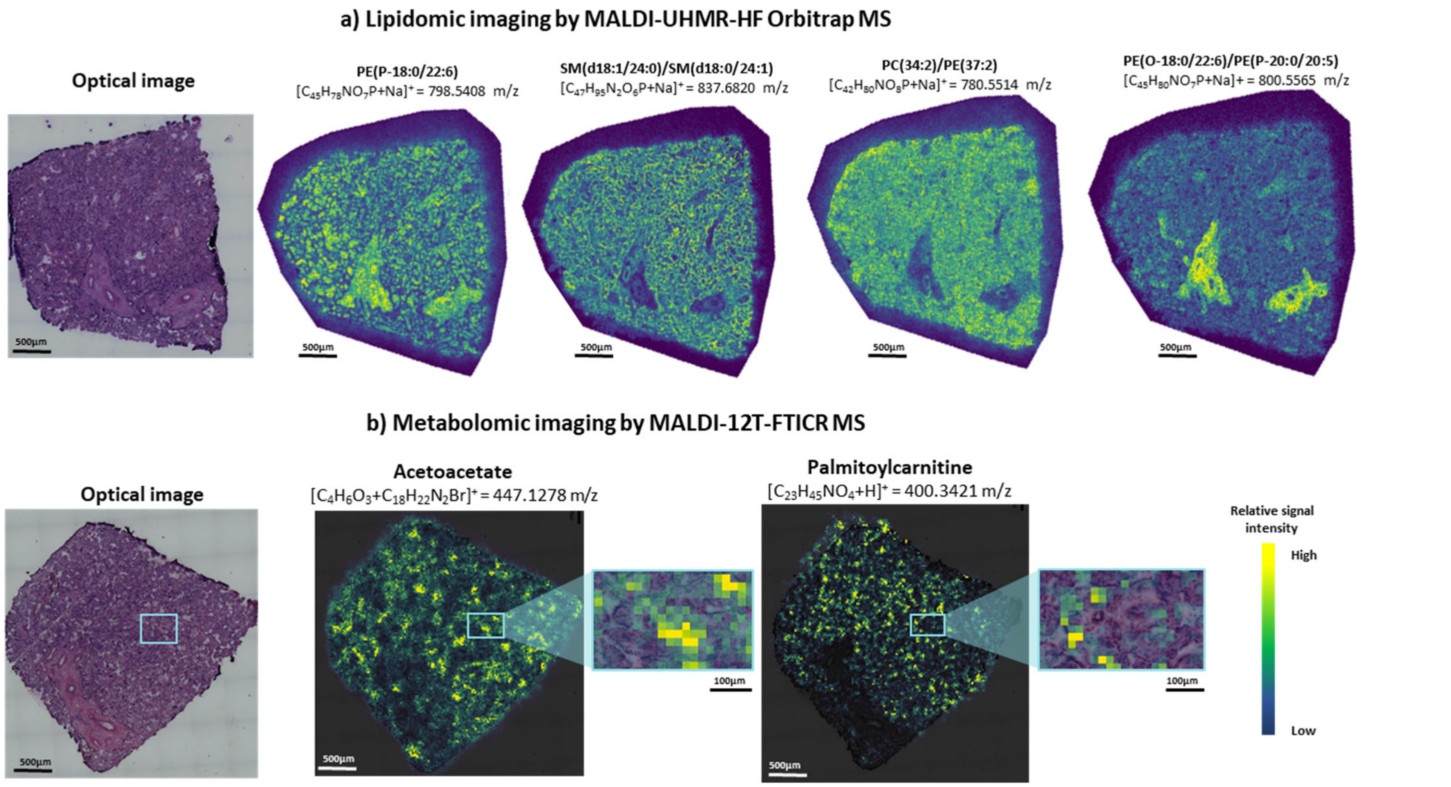

Supplementary Figure 1. Multi-modal MALDI MSI. a) Lipidomic imaging by MALDI-UHMR-HF Orbitrap MS. H&E-stained optical image and example ion images of section 2, from left to right in the following order: lipids localized in core and stem villi; lipid localized in STB; lipids present in both, STB and core, compartments; lipids detected in stem villi. b) Metabolomic imaging by MALDI-12T-FTICR MS. H&E-stained optical image of placental section 4 and example ion images of acetoacetate and palmitoylcarnitine detected in core and STB, respectively.

a) **Protein: sp|O00468|AGRIN_HUMAN; Peptide: R.SIESTLDDLFR.N**

i) Dataset: MIPI_microPOTS_Placenta_19_R2_Core_03Jan23_Bart_BEH-CoA-23-11-19; scan number for MS/MS: 76059

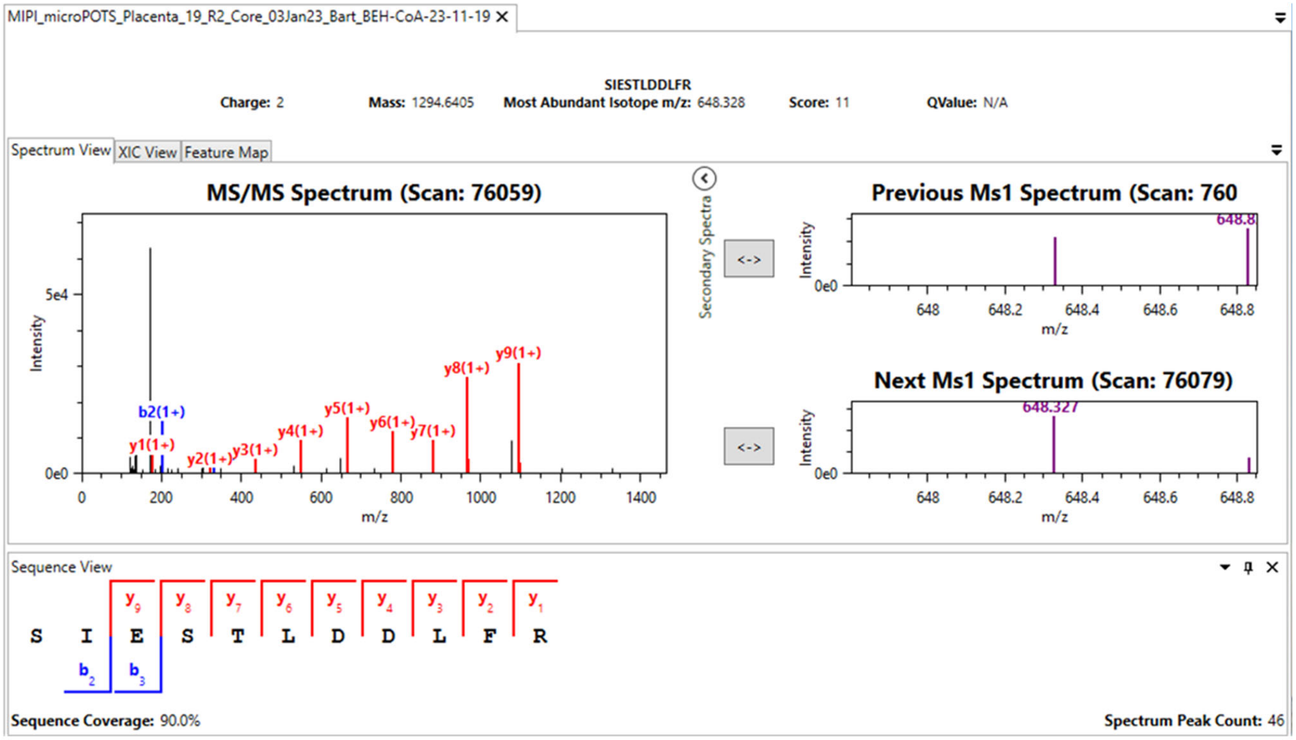

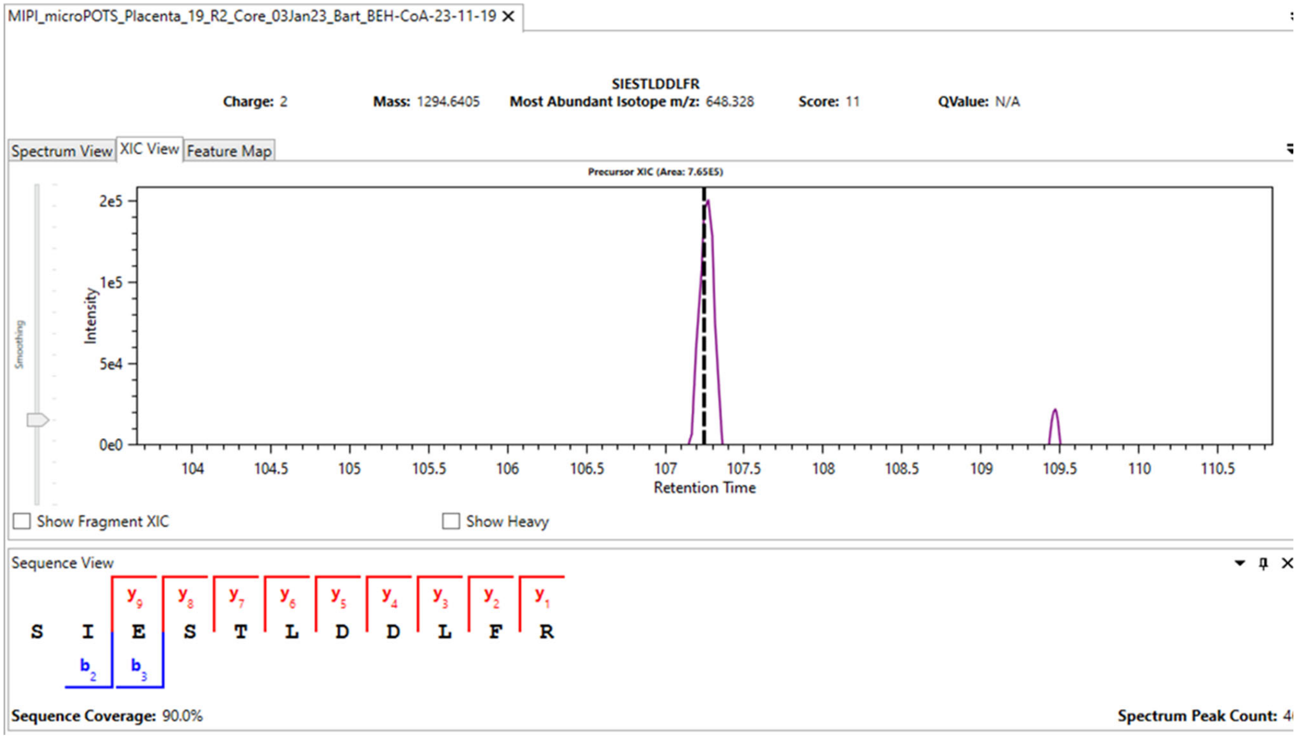

ii) Dataset: MIPI_microPOTS_Placenta_26_R5_Core_03Jan23_Bart_BEH-CoA-23-11-19; scan number for MS/MS: 79023.

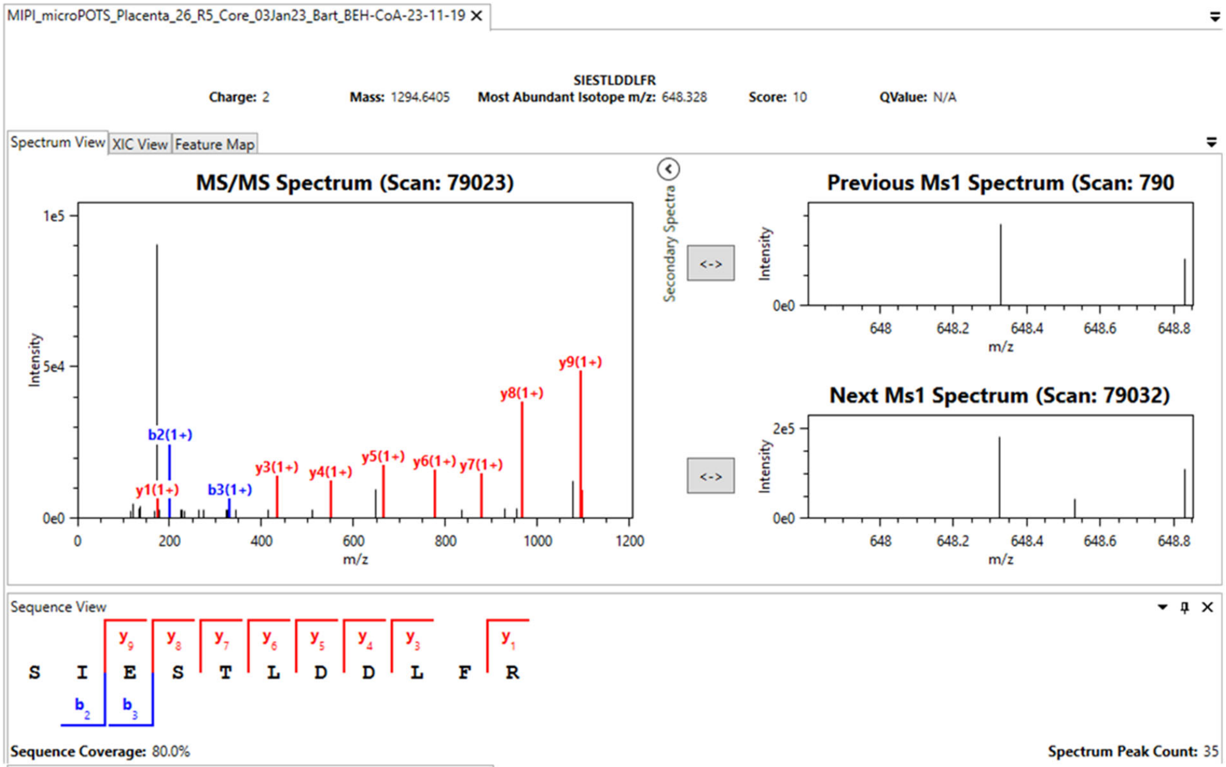

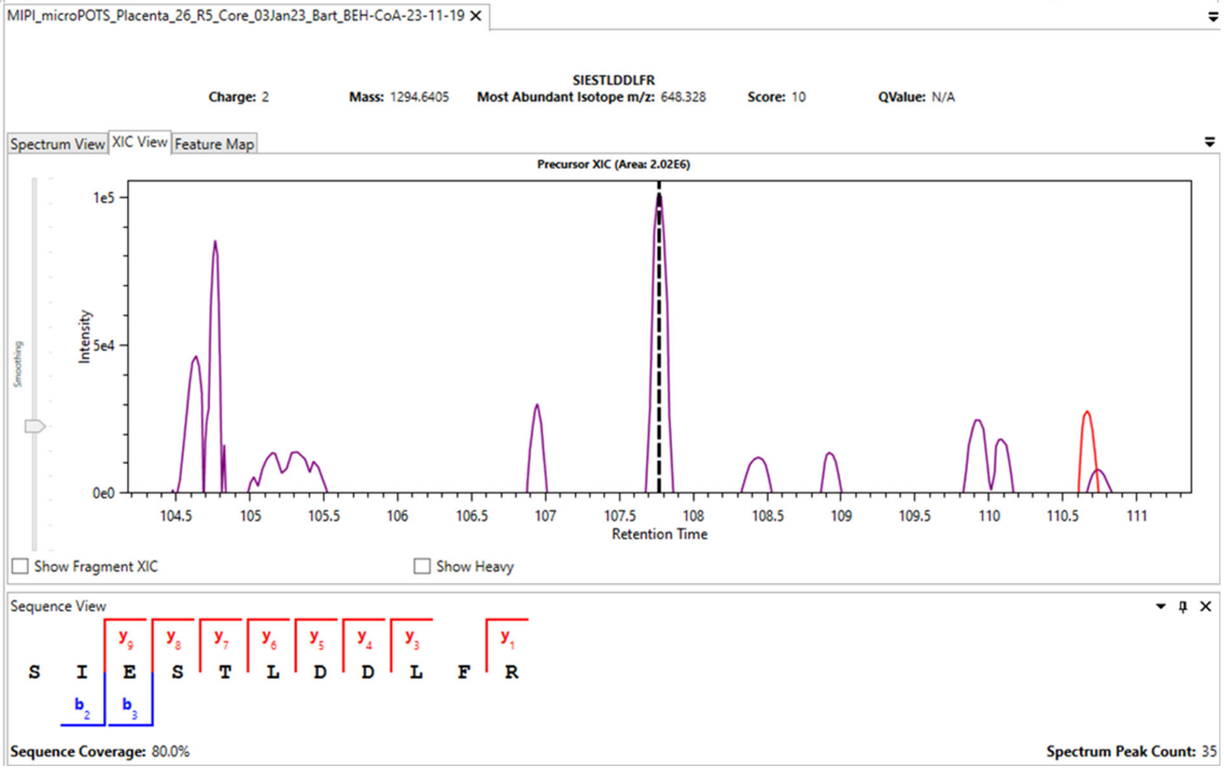

iii) Dataset: MIPI_microPOTS_Placenta_26_R4_Core_03Jan23_Bart_BEH-CoA-23-11-19; Scan Number for MS/MS: 80041.

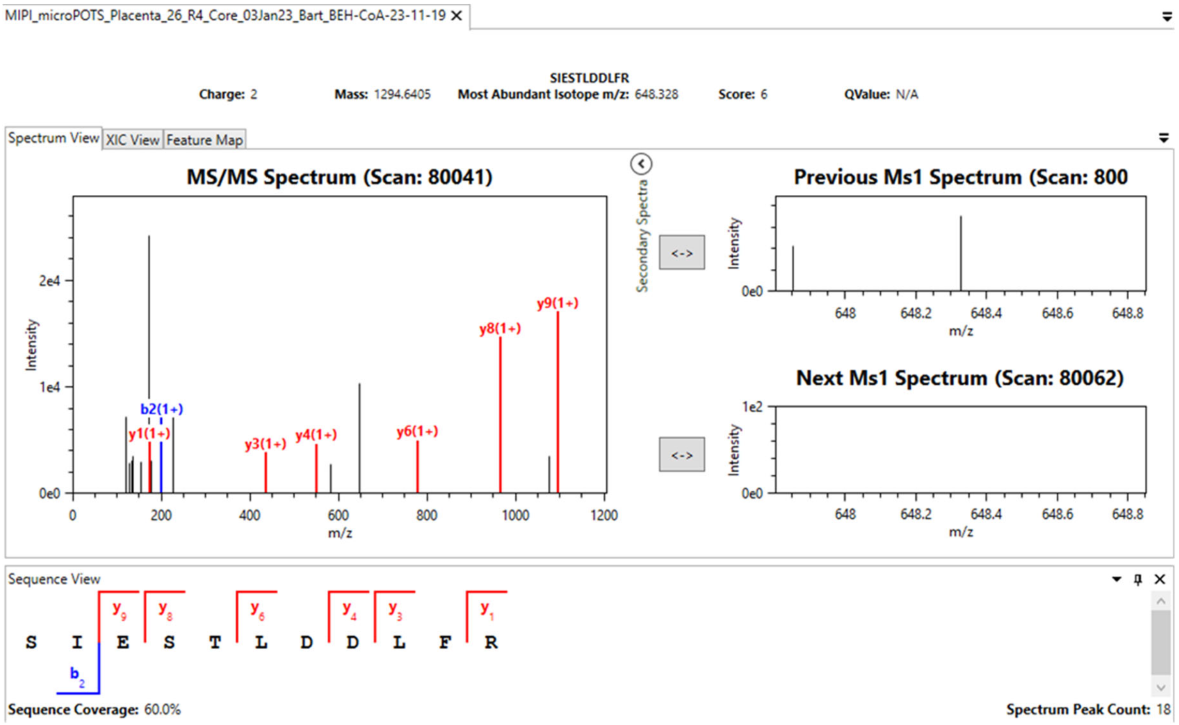

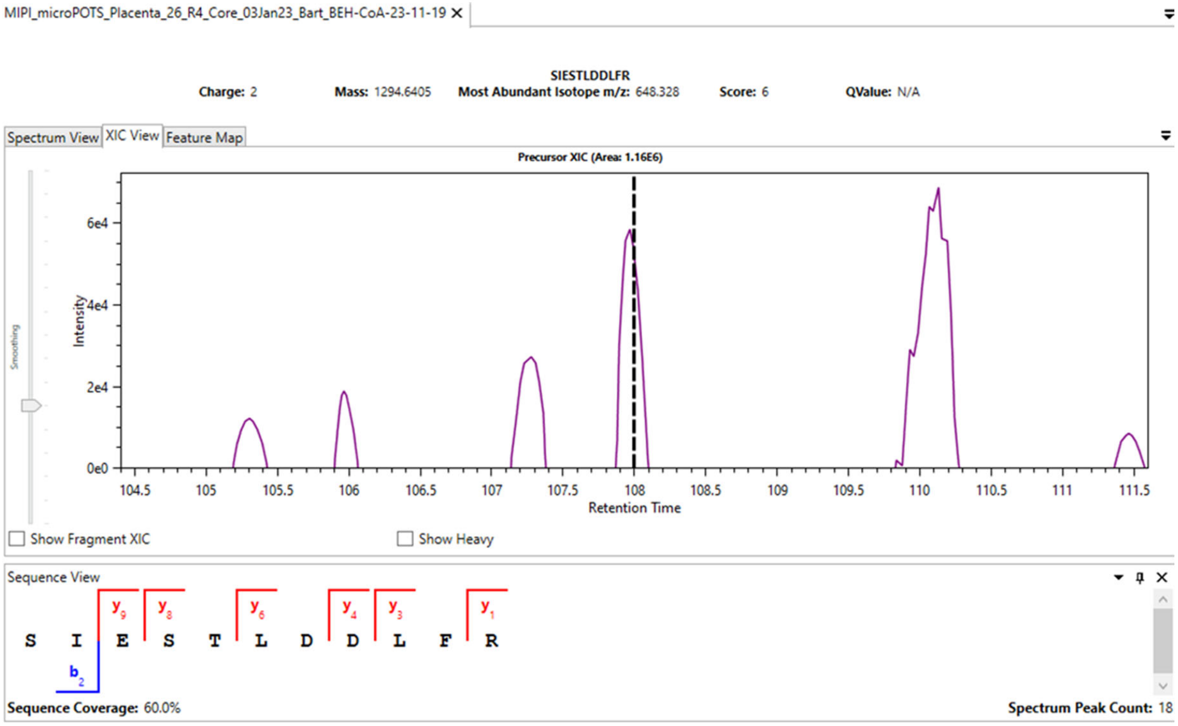

iv) Dataset: MIPI_microPOTS_Placenta_26_R2_Core_03Jan23_Bart_BEH-CoA-23-11-19; Scan Number for MS/MS: 80120.

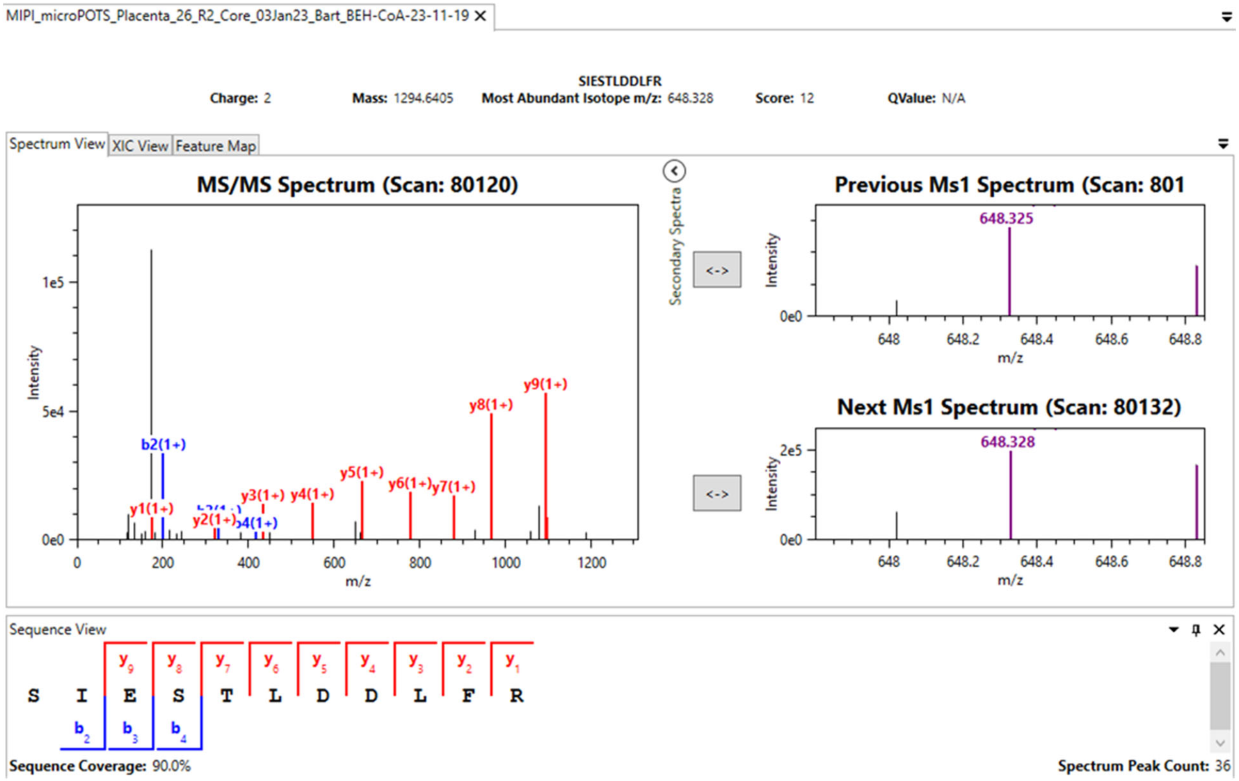

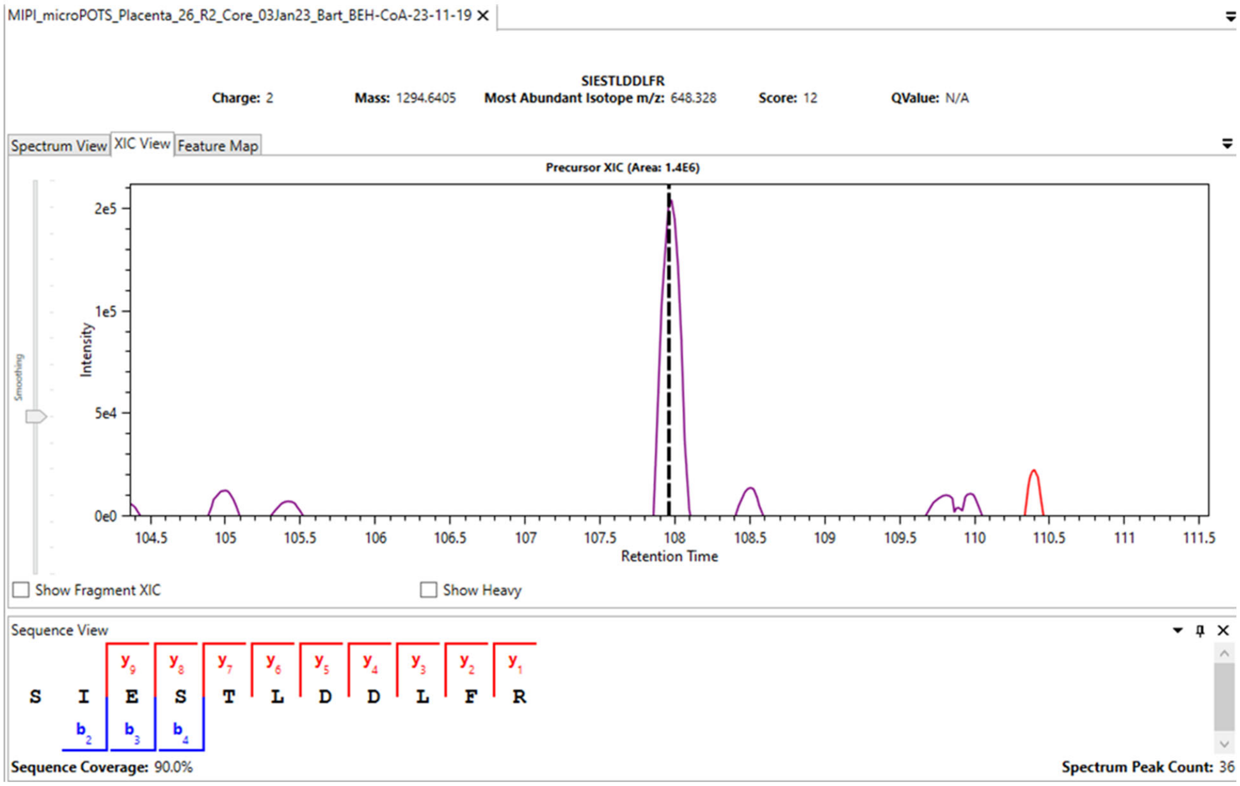

b) Protein: sp|O14975|S27A2_HUMAN; Peptide: R.DETLTYAQVDR.R

i) Dataset: MIPI_microPOTS_Placenta_19_R2_STB_03Jan23_Bart_BEH-CoA-23-11-19; Scan Number for MS/MS: 25760.

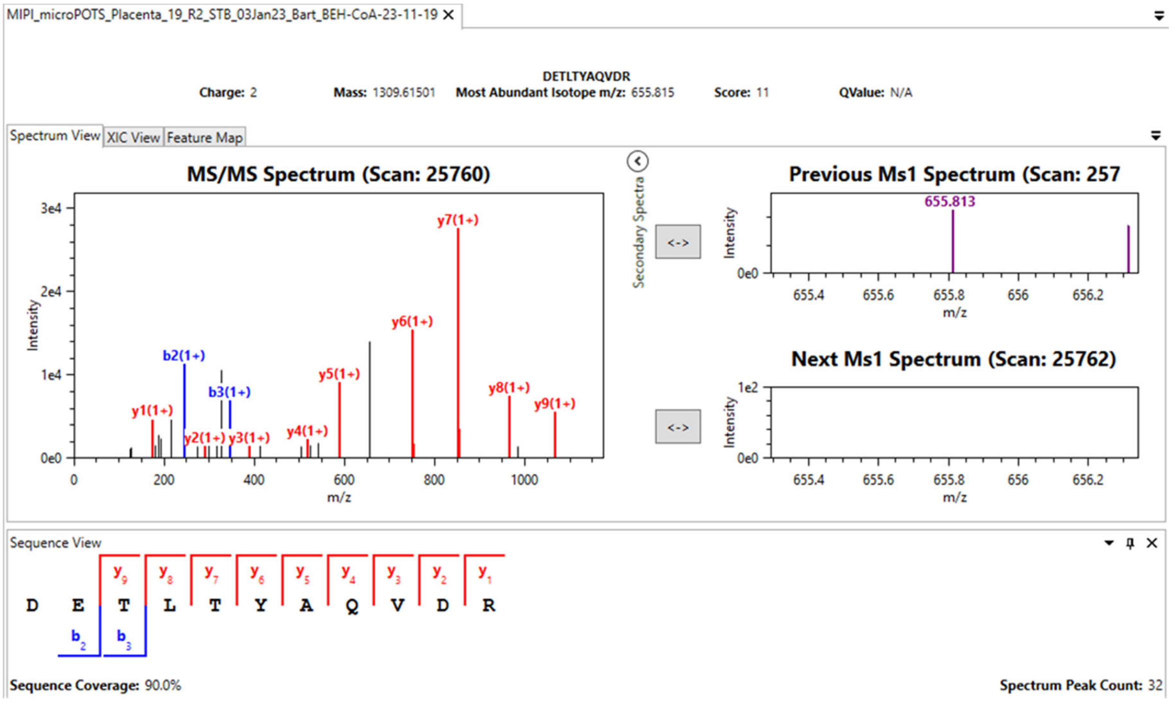

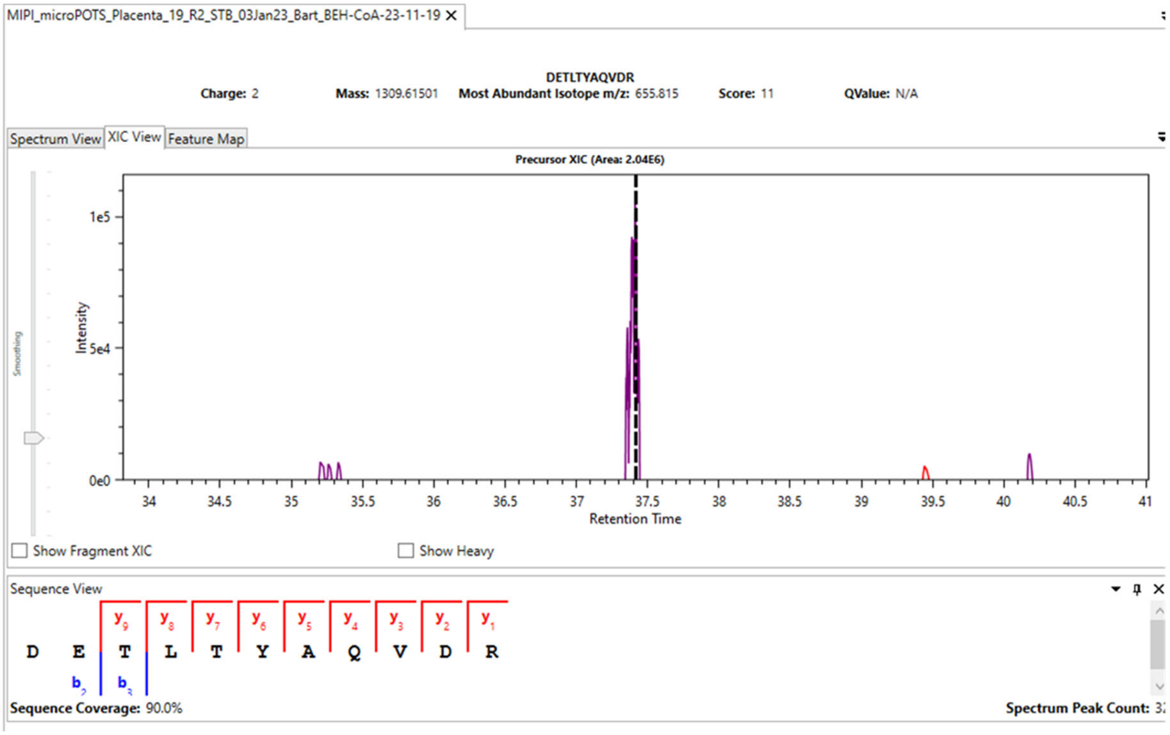

ii) Dataset: MIPI_microPOTS_Placenta_23_R2_STB_03Jan23_Bart_BEH-CoA-23-11-19; Scan Number for MS/MS: 26152.

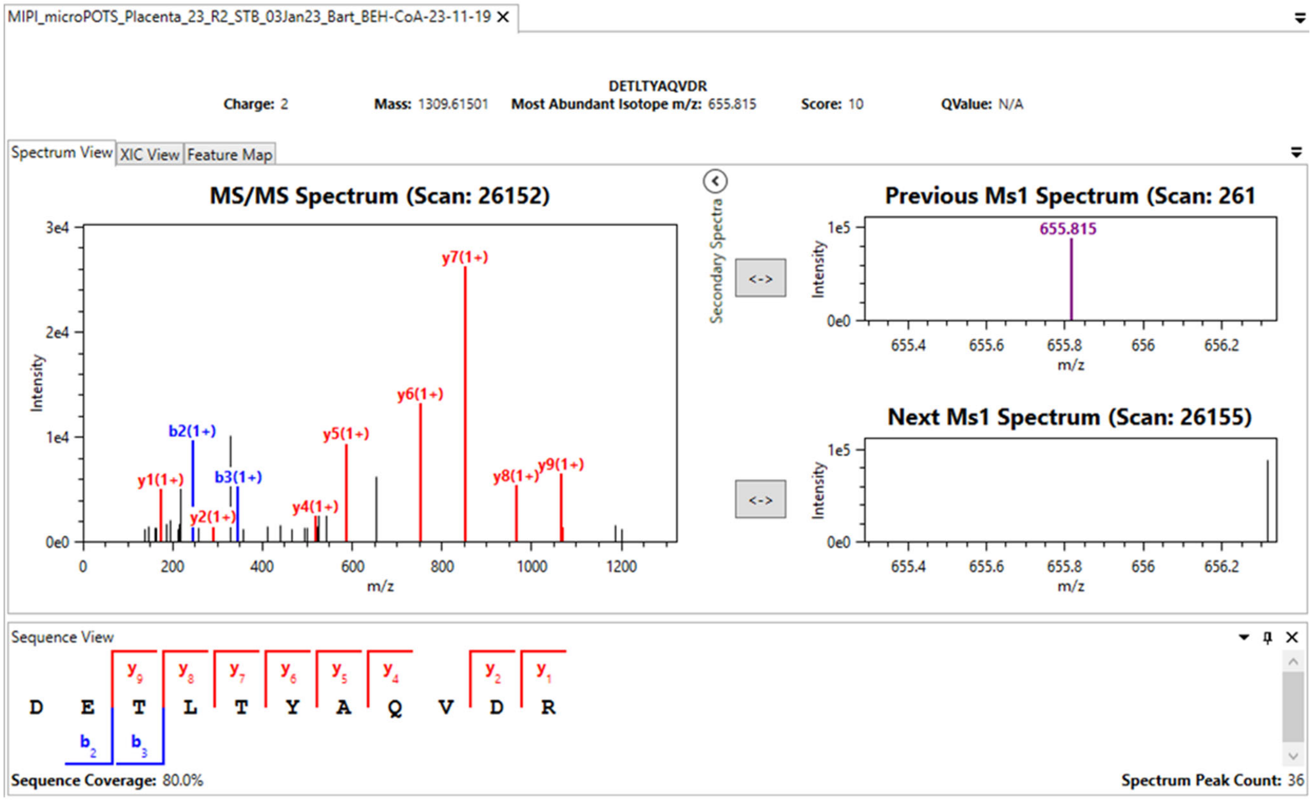

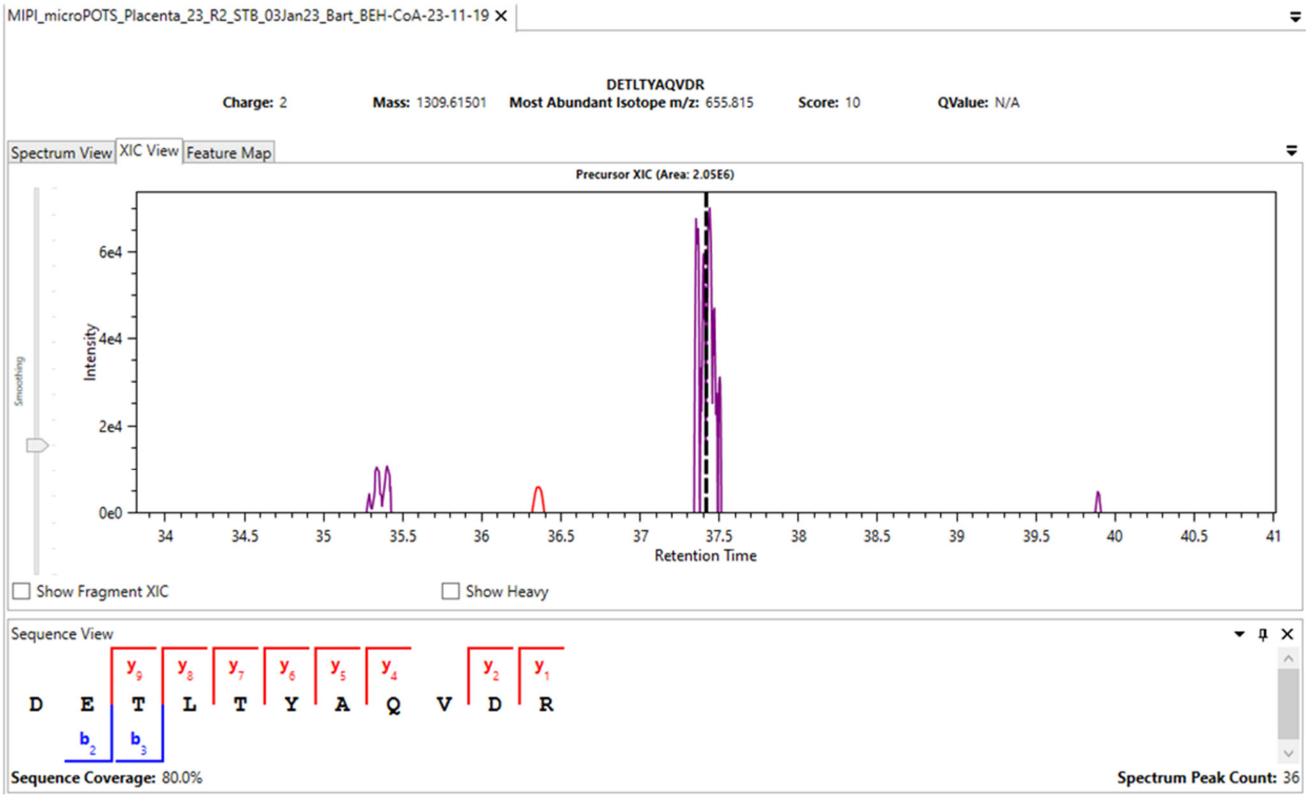

iii) Dataset: MIPI_microPOTS_Placenta_19_R5_STB_03Jan23_Bart_BEH-CoA-23-11-19; Scan Number for MS/MS: 26851

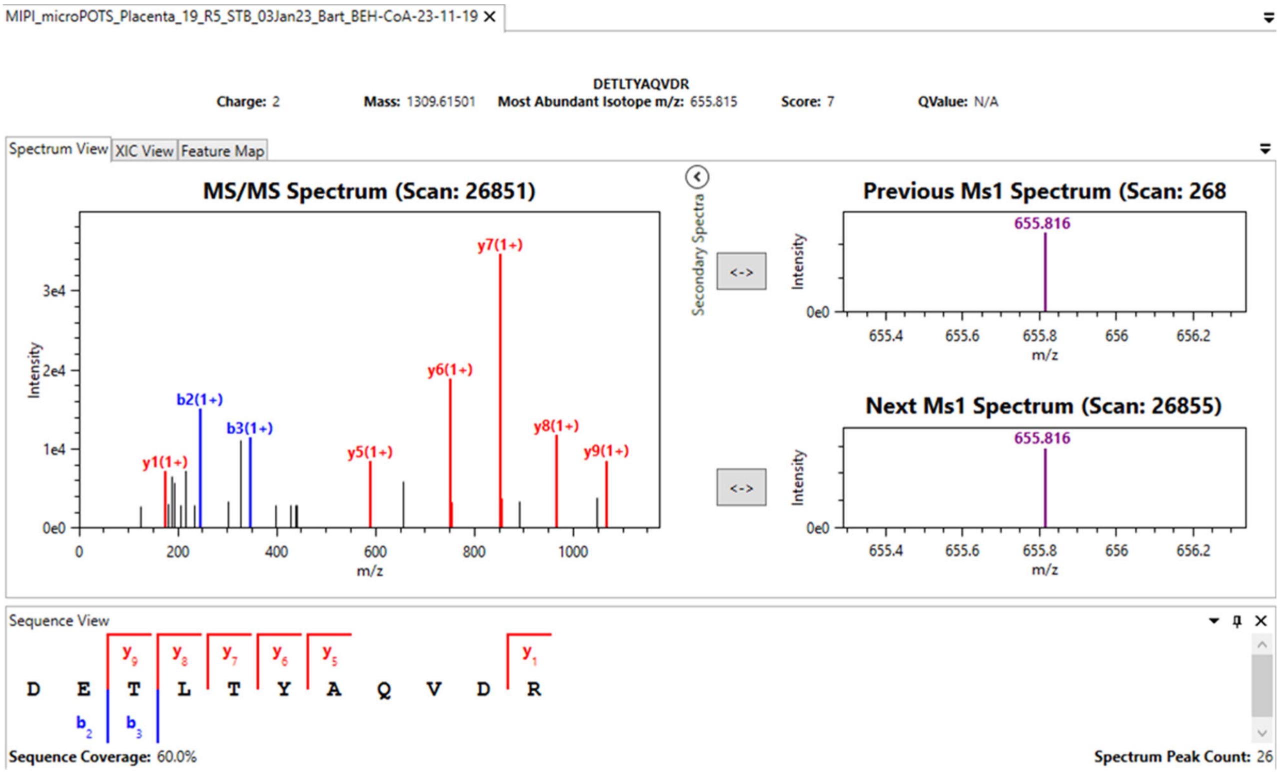

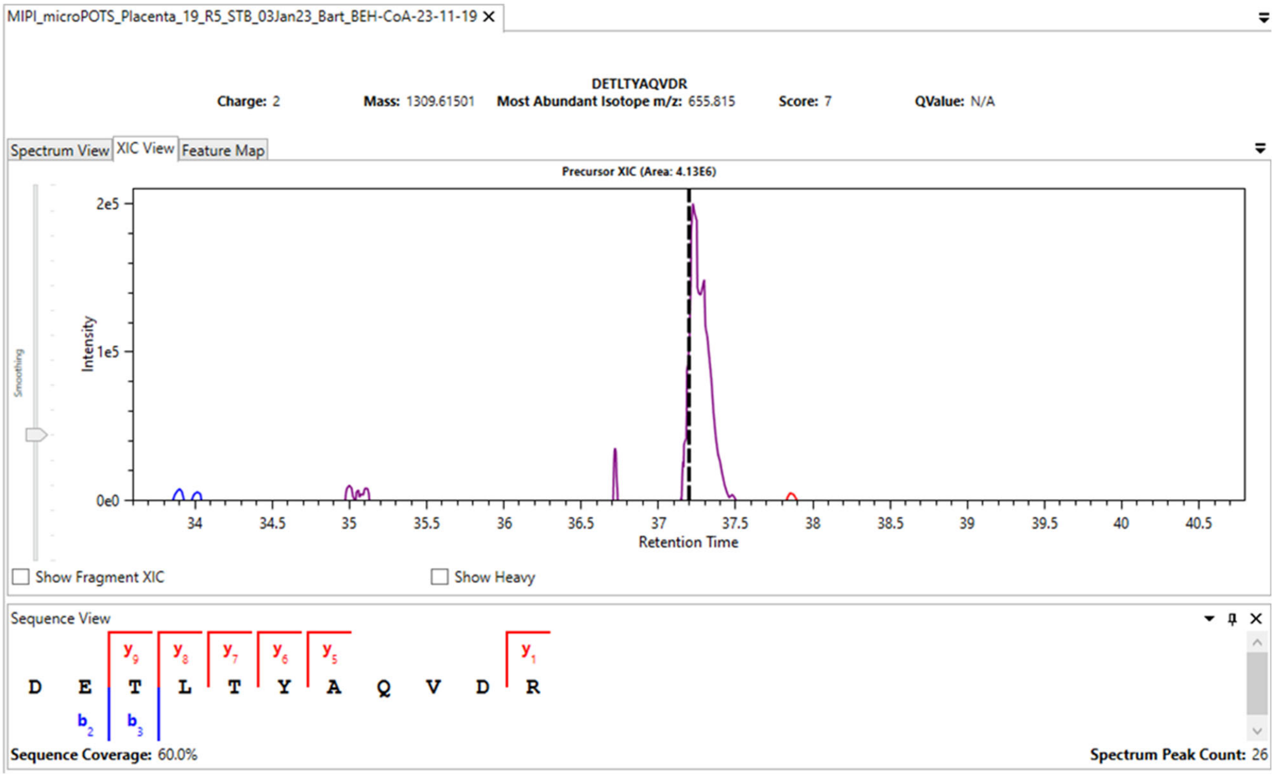

iv) Dataset: MIPI_microPOTS_Placenta_19_R5_STB_03Jan23_Bart_BEH-CoA-23-11-19; Scan Number for MS/MS: 26866

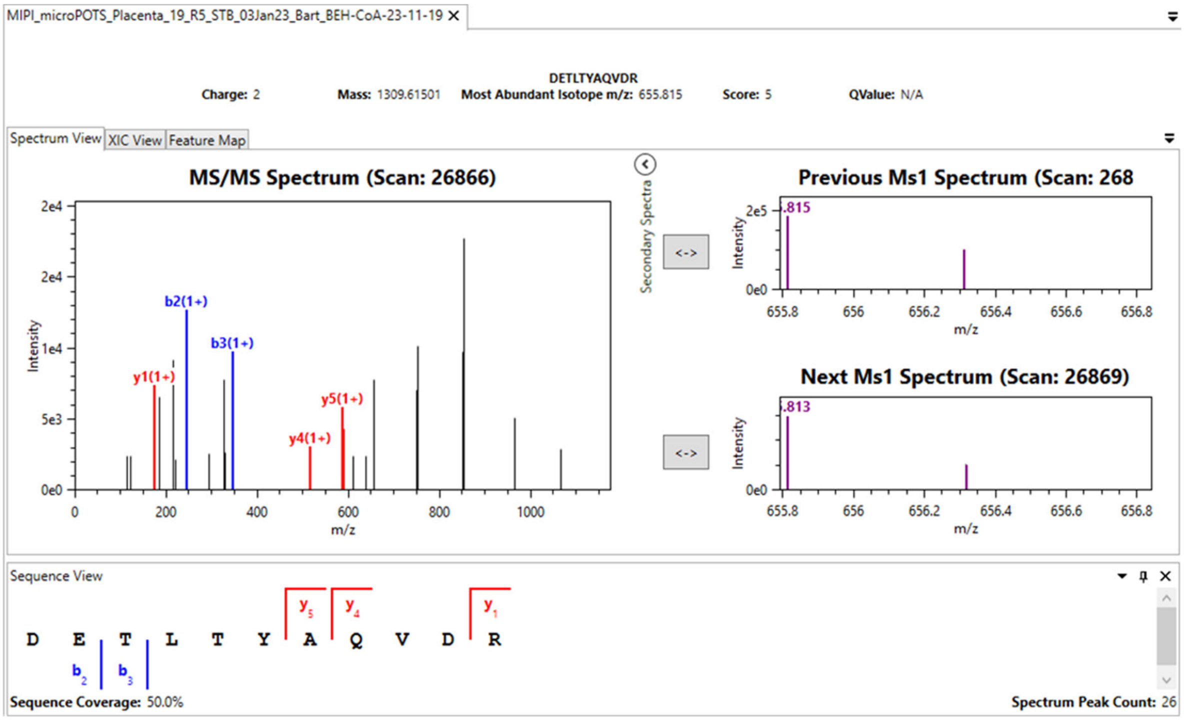

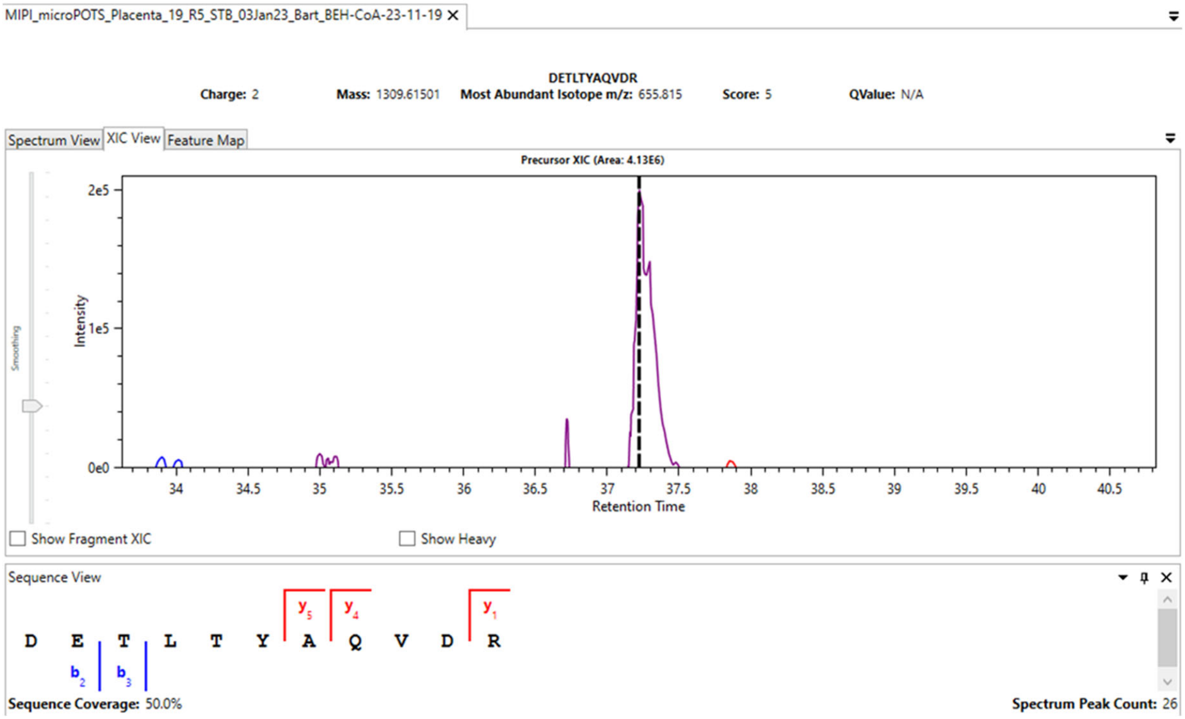

v) Dataset: MIPI_microPOTS_Placenta_26_R2_STB_03Jan23_Bart_BEH-CoA-23-11-19; Scan Number for MS/MS: 27027

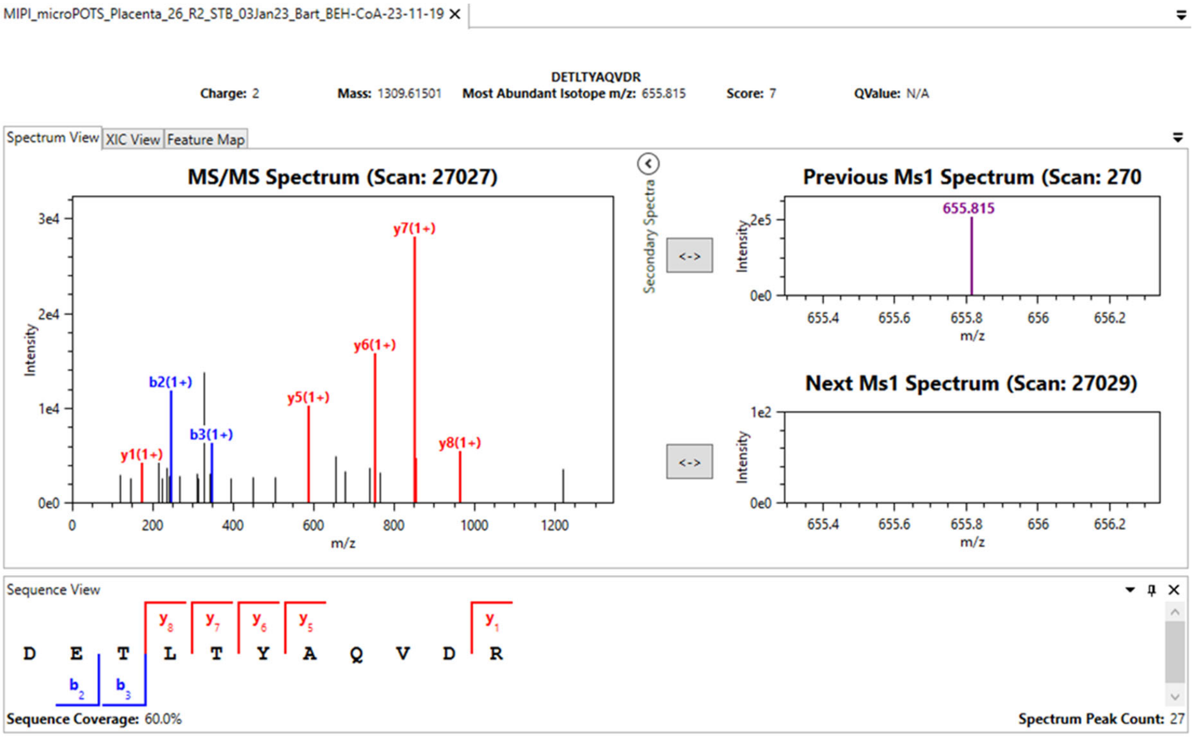

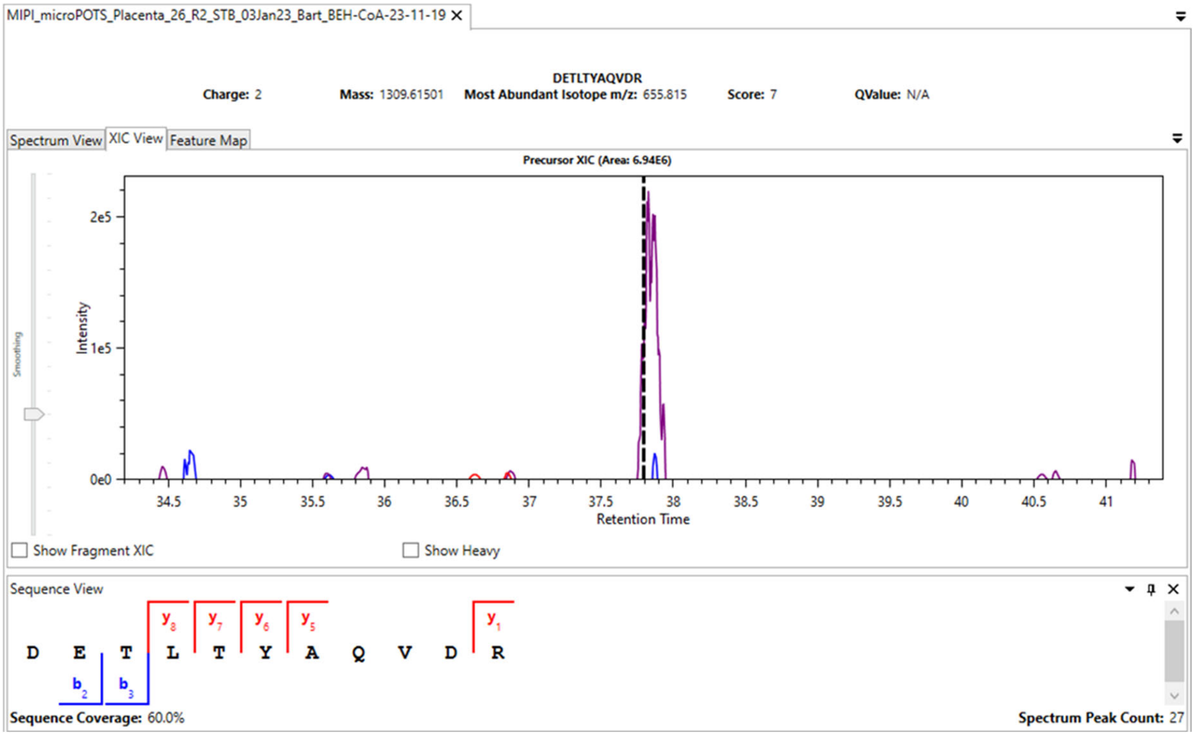

c) Protein: sp|O15269|SPTC1_HUMAN; Peptide: R.VVVTVEQTEEELER.A

i) Dataset: MIPI_microPOTS_Placenta_26_R1_STB_03Jan23_Bart_BEH-CoA-23-11-19; Scan Number for MS/MS: 47200.

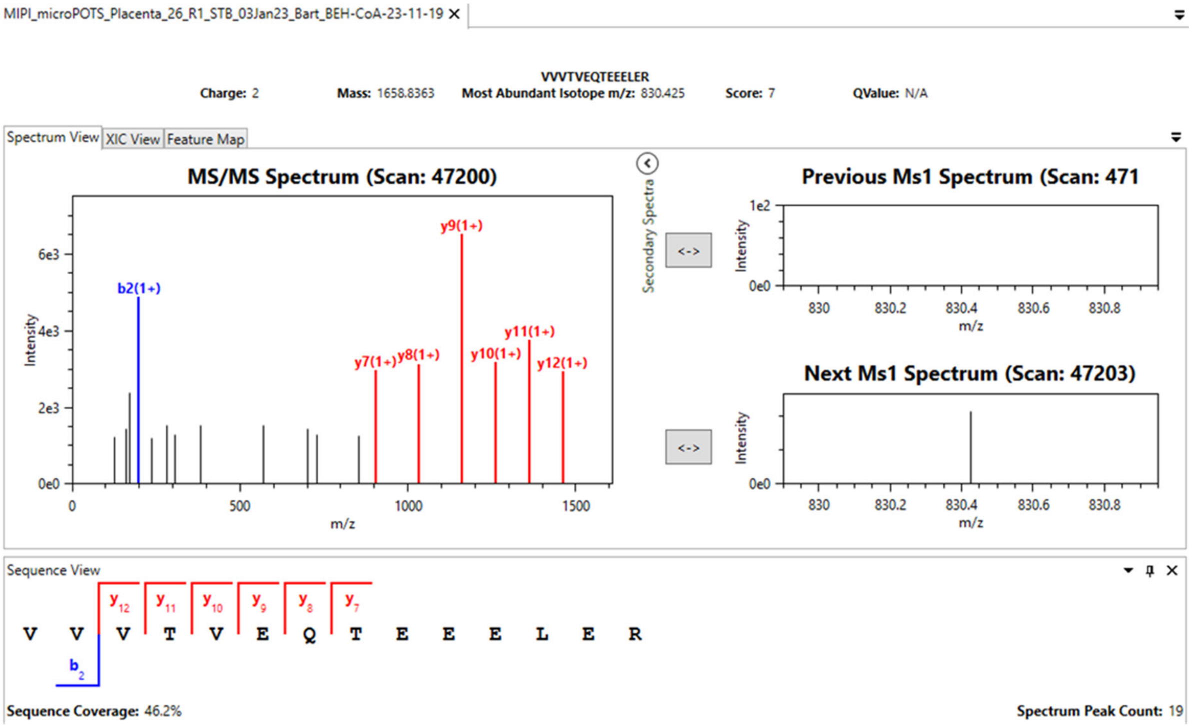

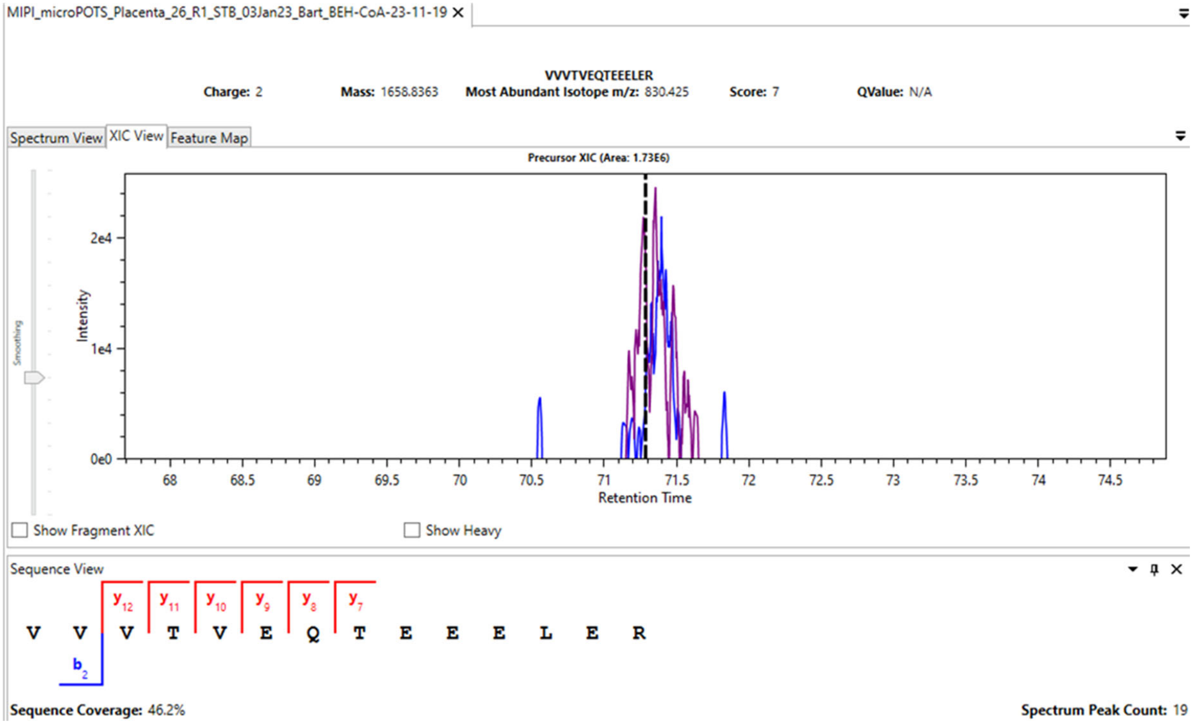

ii) Dataset: MIPI_microPOTS_Placenta_19_R3_STB_03Jan23_Bart_BEH-CoA-23-11-19; Scan Number for MS/MS: 47227

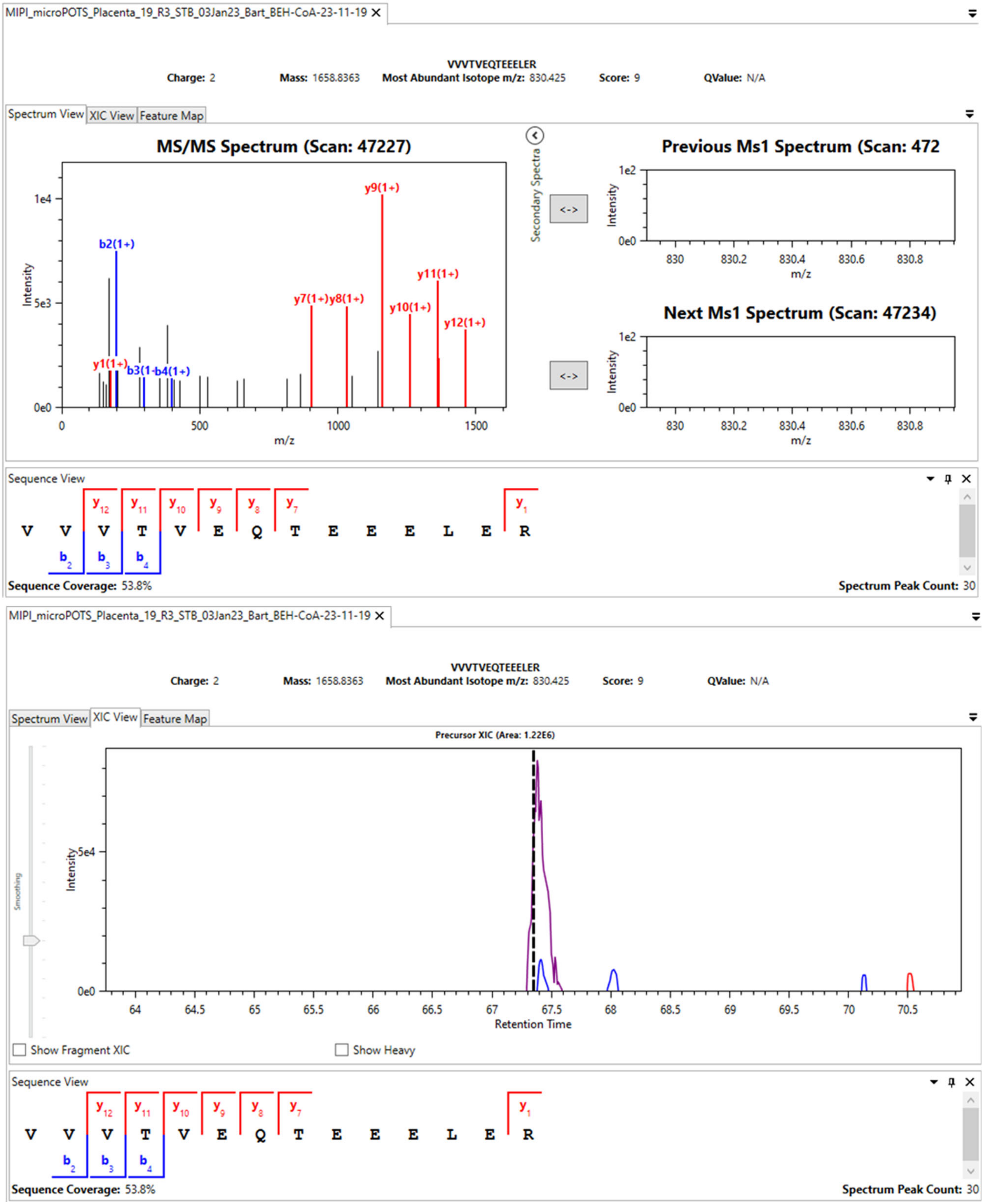

iii) Dataset: MIPI_microPOTS_Placenta_23_R5_STB_03Jan23_Bart_BEH-CoA-23-11-19; Scan Number for MS/MS: 48414.

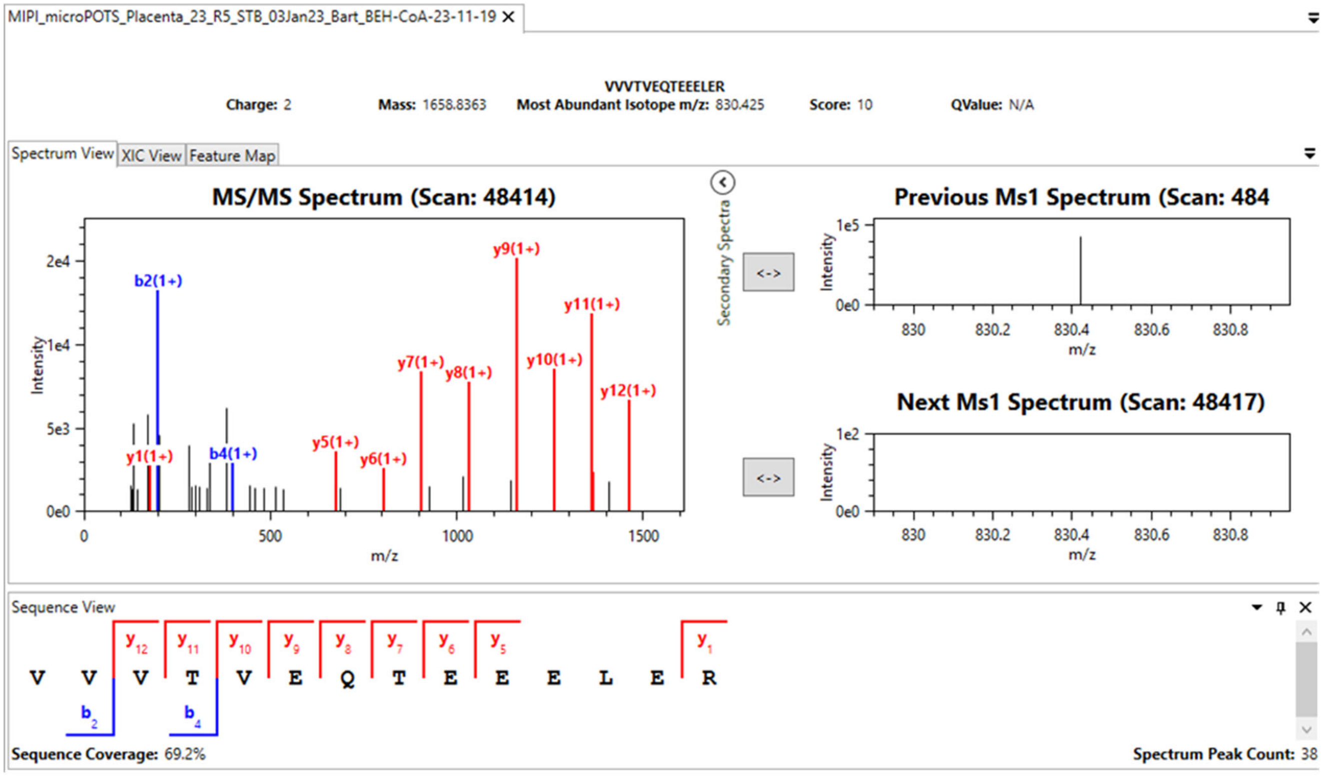

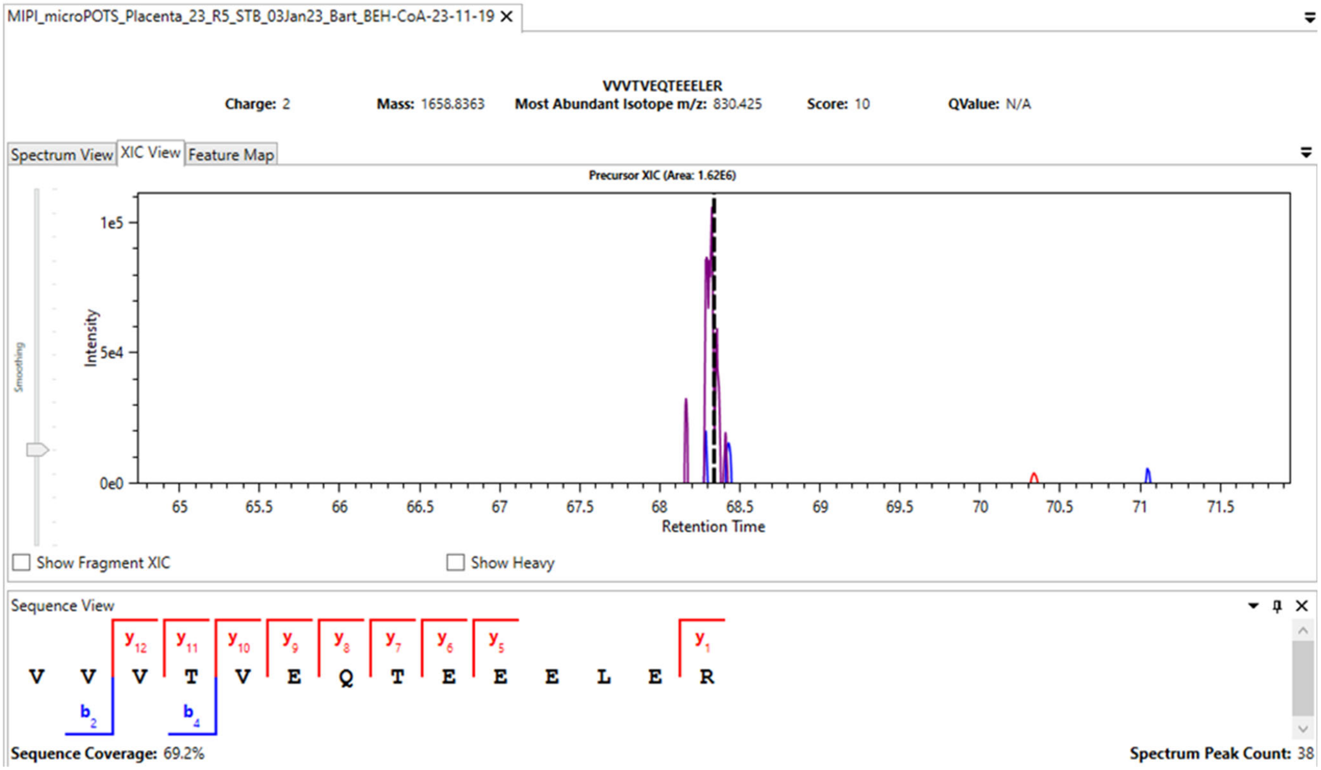

iv) Dataset: MIPI_microPOTS_Placenta_19_R5_STB_03Jan23_Bart_BEH-CoA-23-11-19; Scan Number for MS/MS: 49240.

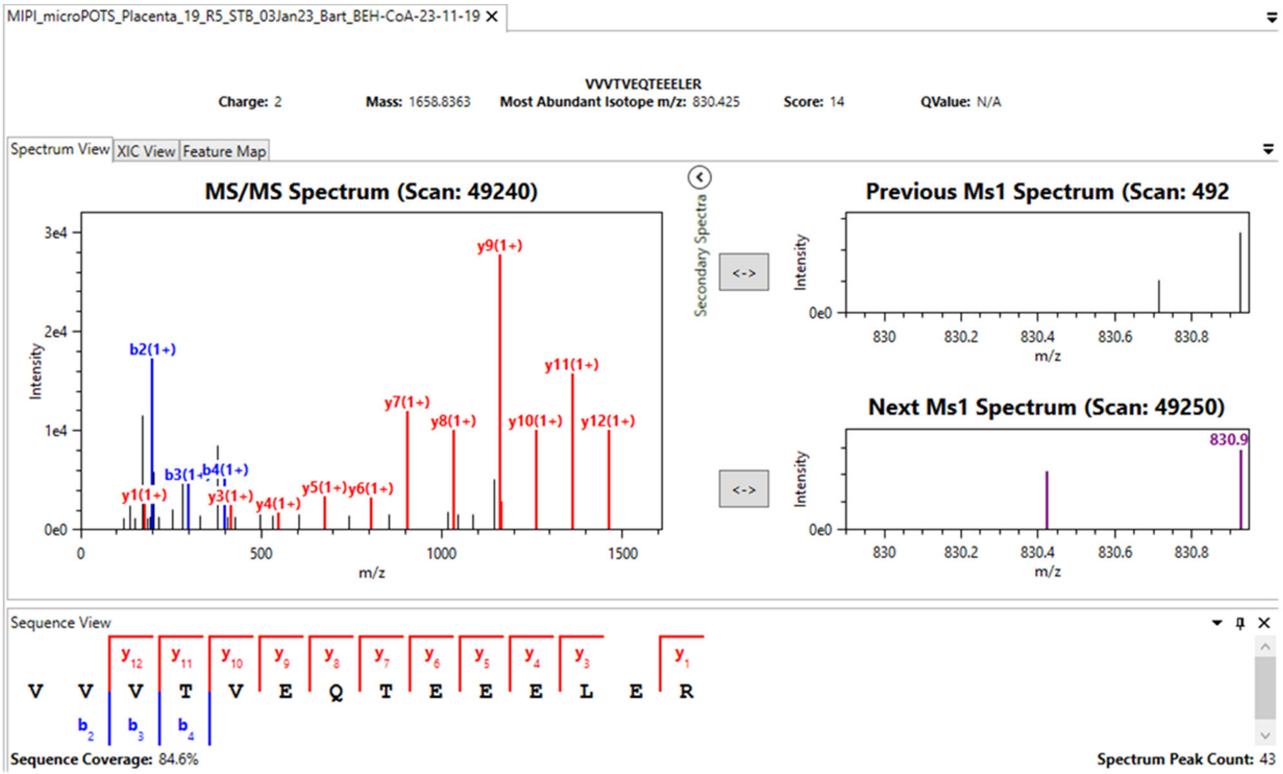

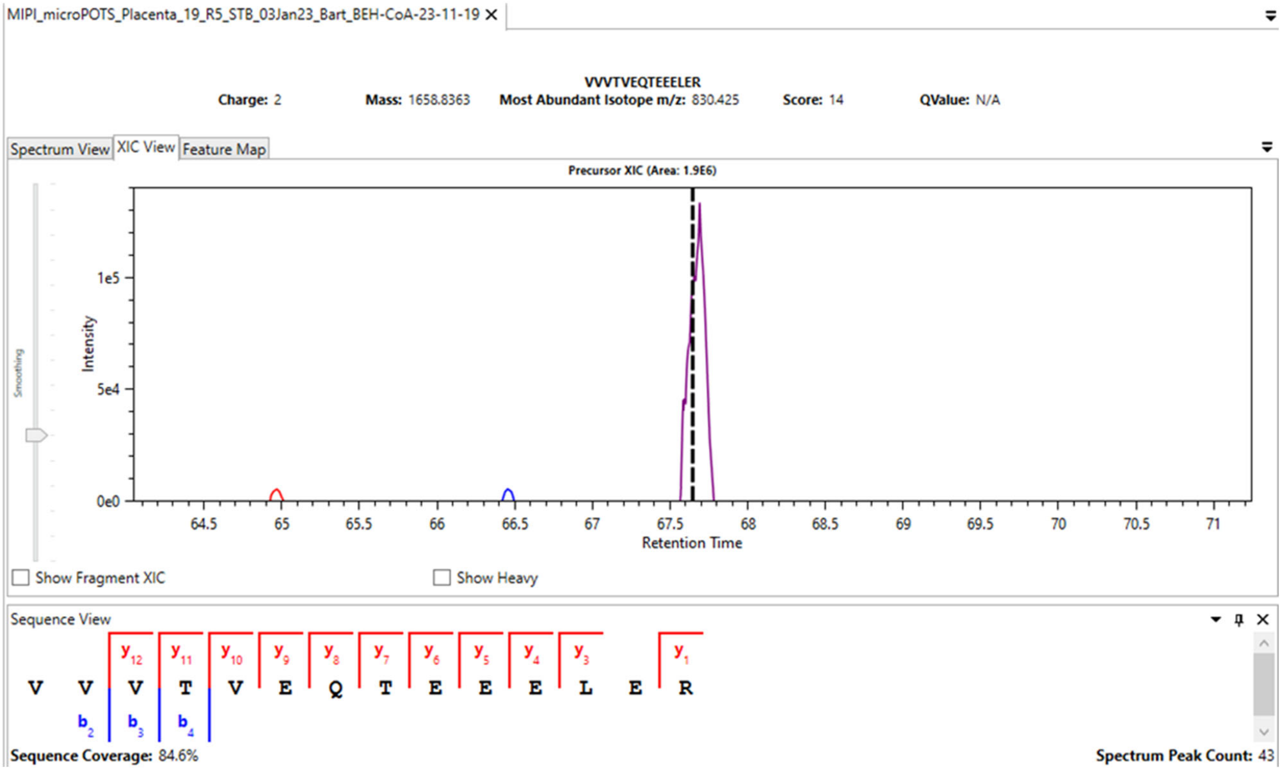

d) Protein: sp|P20674|COX5A_HUMAN; Peptide: R.LNDFASTVR.I

i) Dataset: MIPI_microPOTS_Placenta_19_R3_STB_03Jan23_Bart_BEH-CoA-23-11-19; Scan Number for MS/MS: 24217.

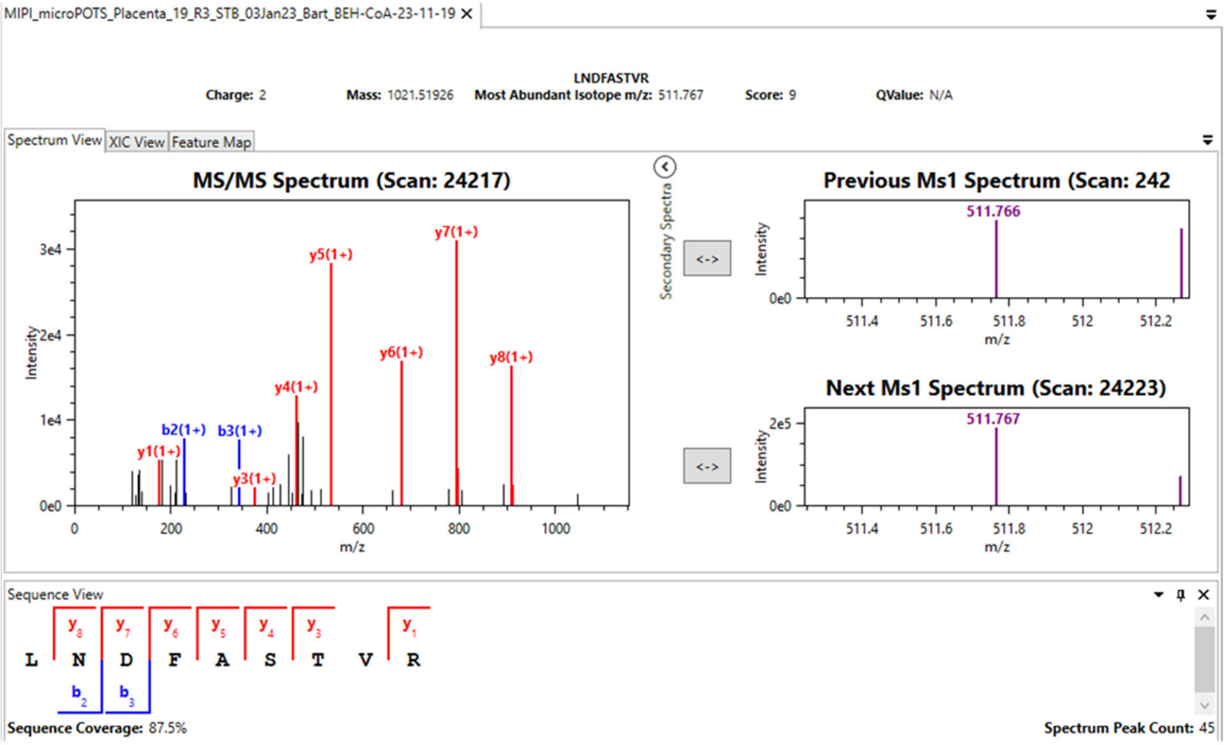

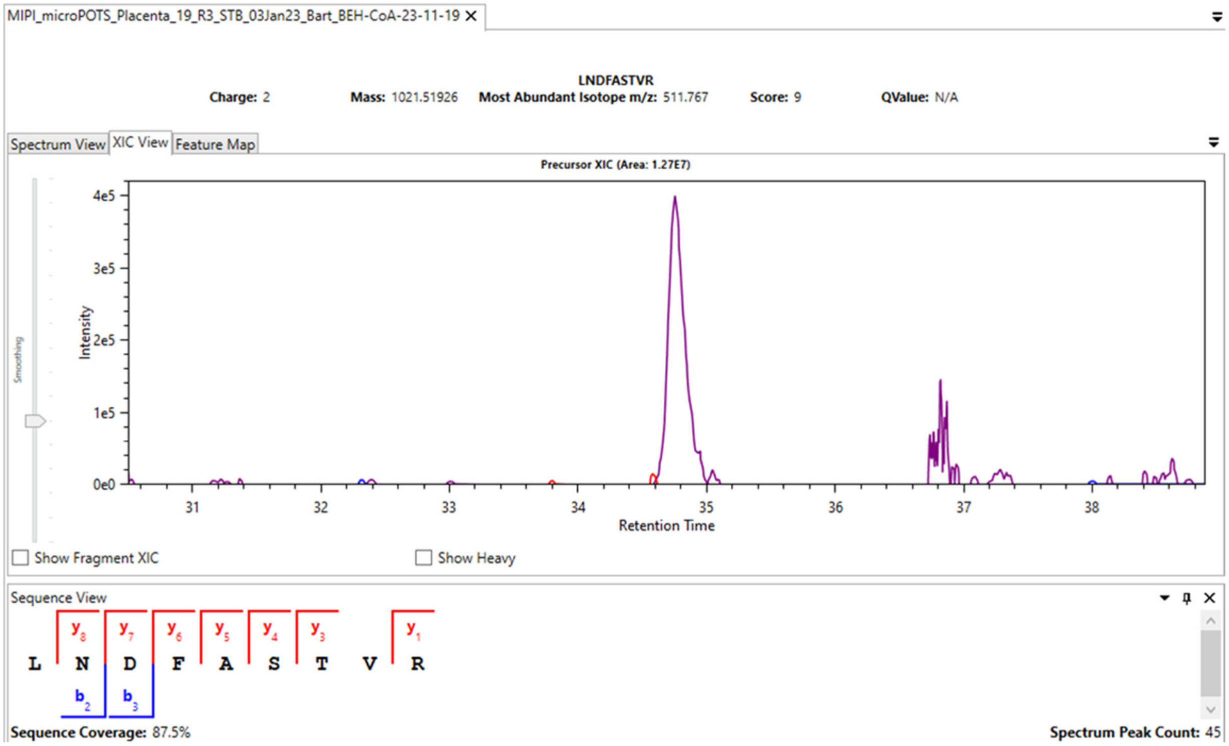

ii) Dataset: MIPI_microPOTS_Placenta_26_R1_STB_03Jan23_Bart_BEH-CoA-23-11-19; Scan Number for MS/MS: 24239.

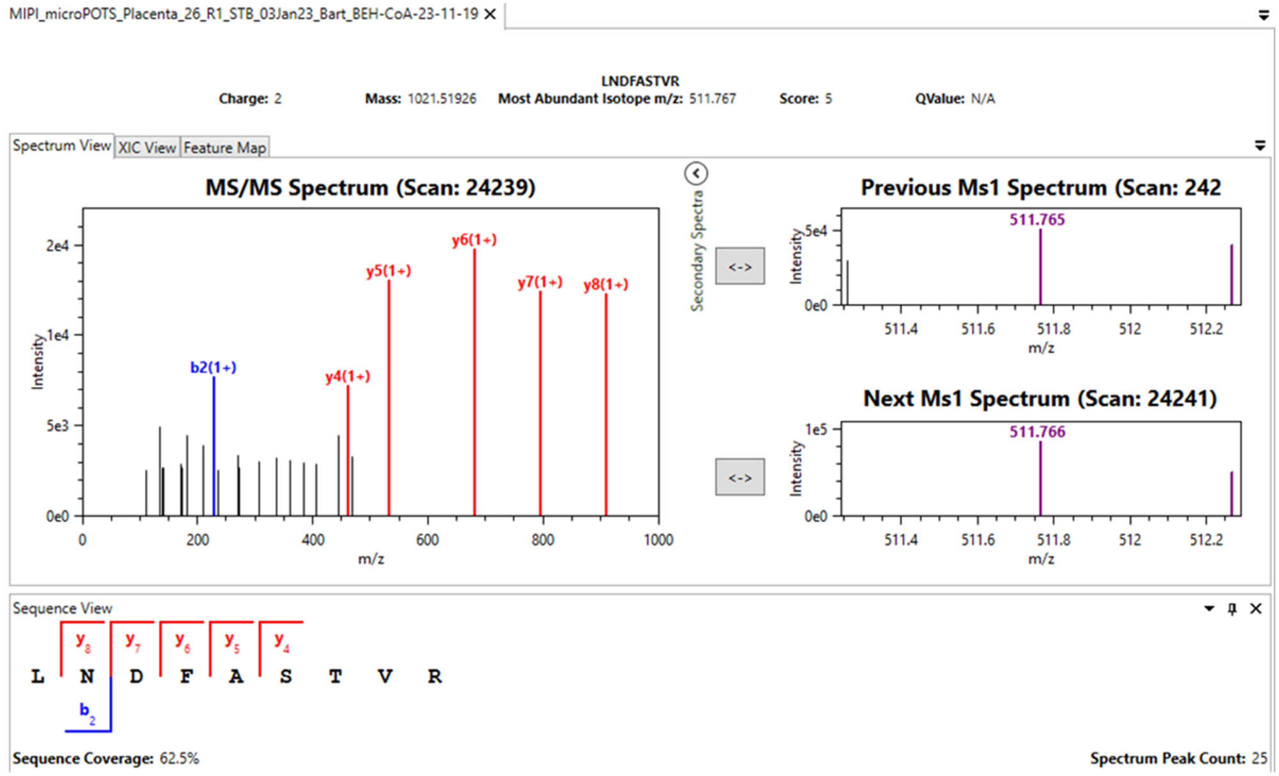

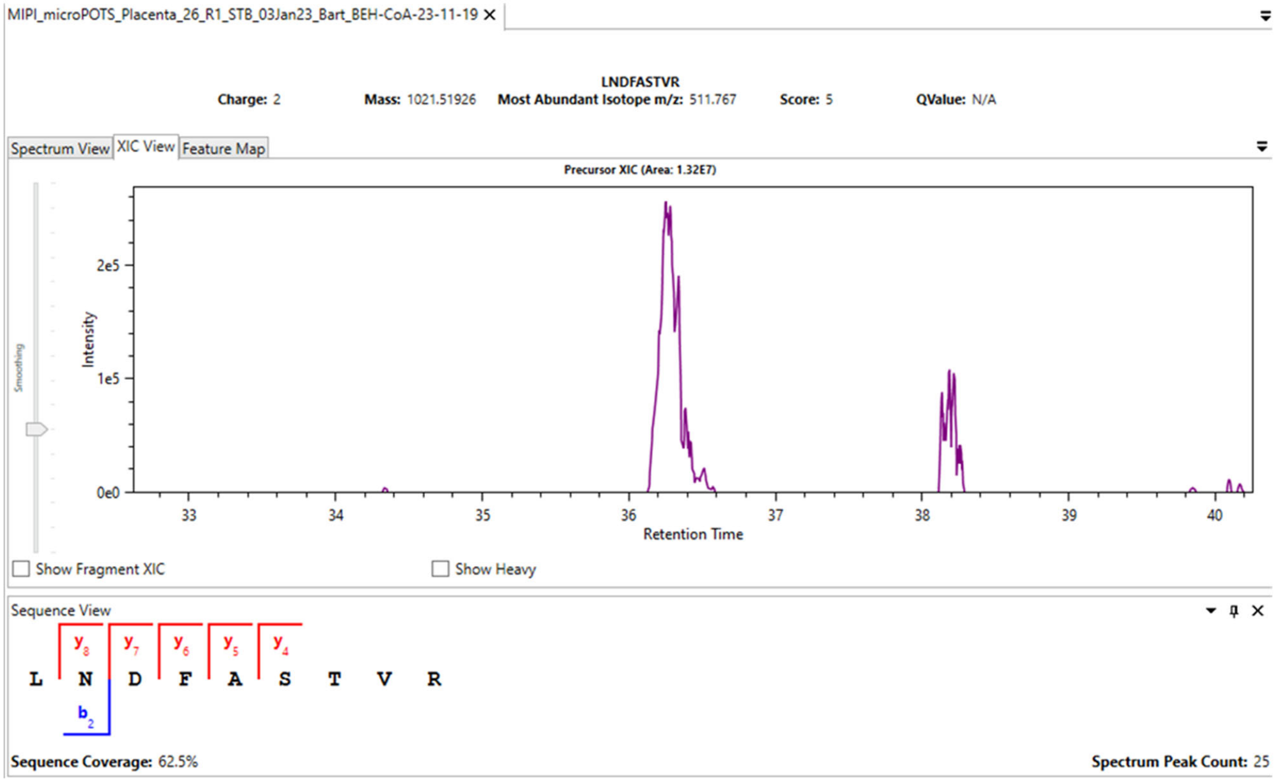

iii) Dataset: MIPI_microPOTS_Placenta_23_R1_STB_03Jan23_Bart_BEH-CoA-23-11-19; Scan Number for MS/MS: 24833

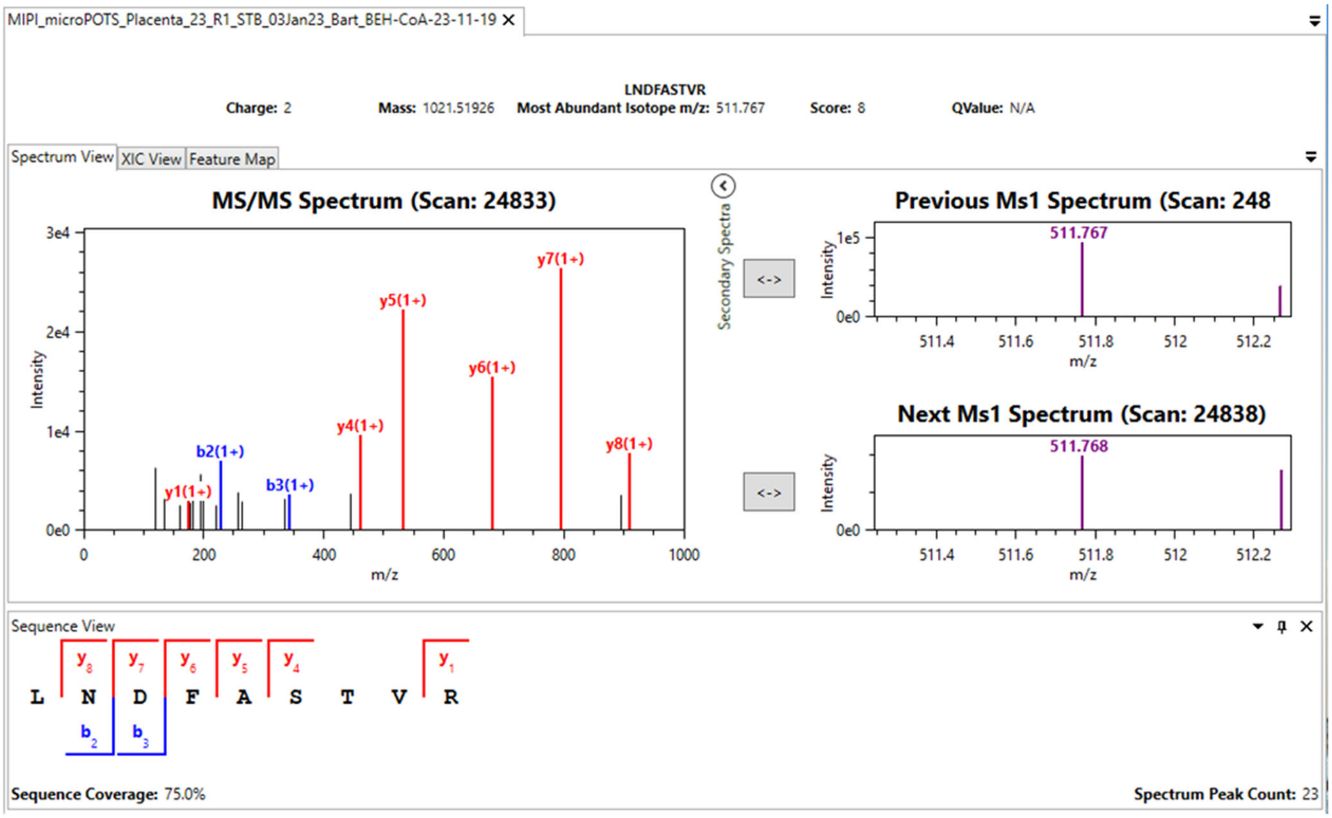

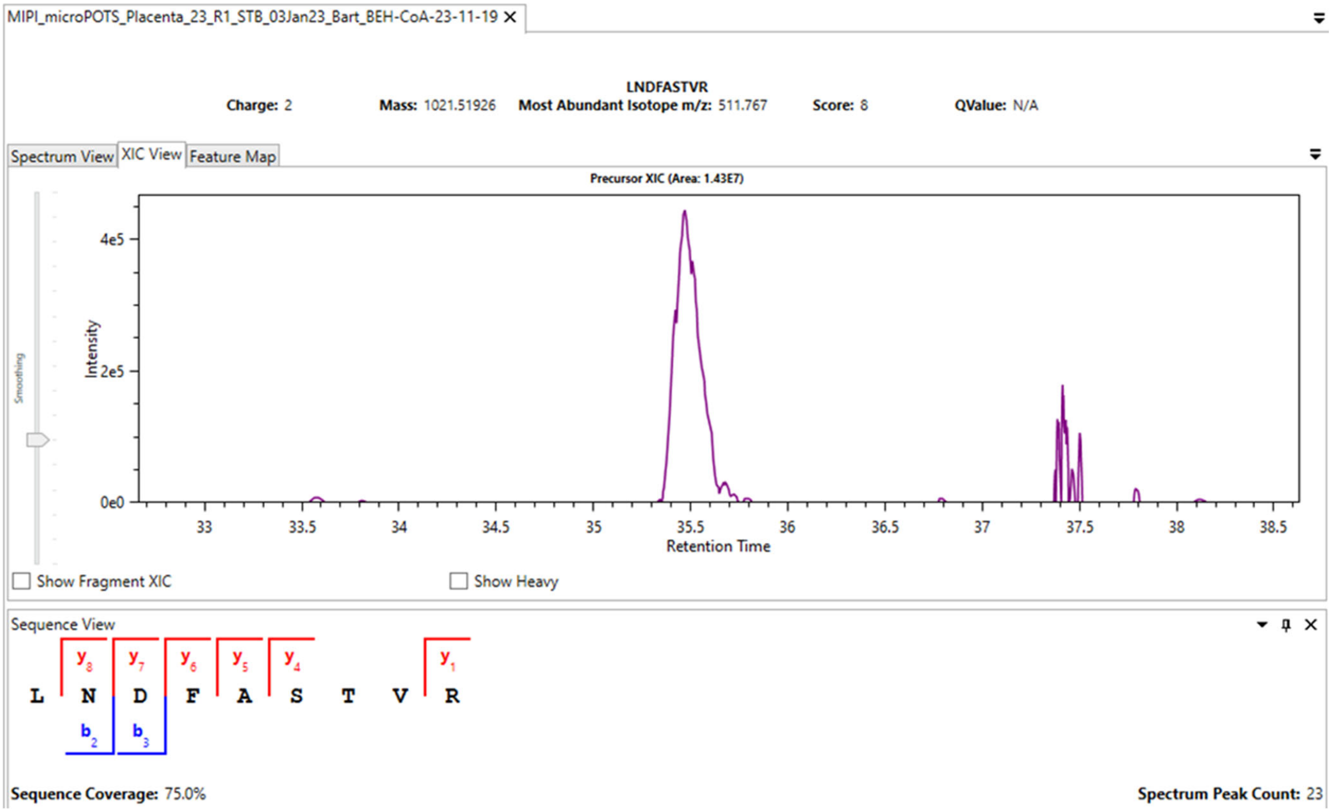

iv) Dataset: MIPI_microPOTS_Placenta_23_R2_STB_03Jan23_Bart_BEH-CoA-23-11-19; Scan Number for MS/MS: 24833.

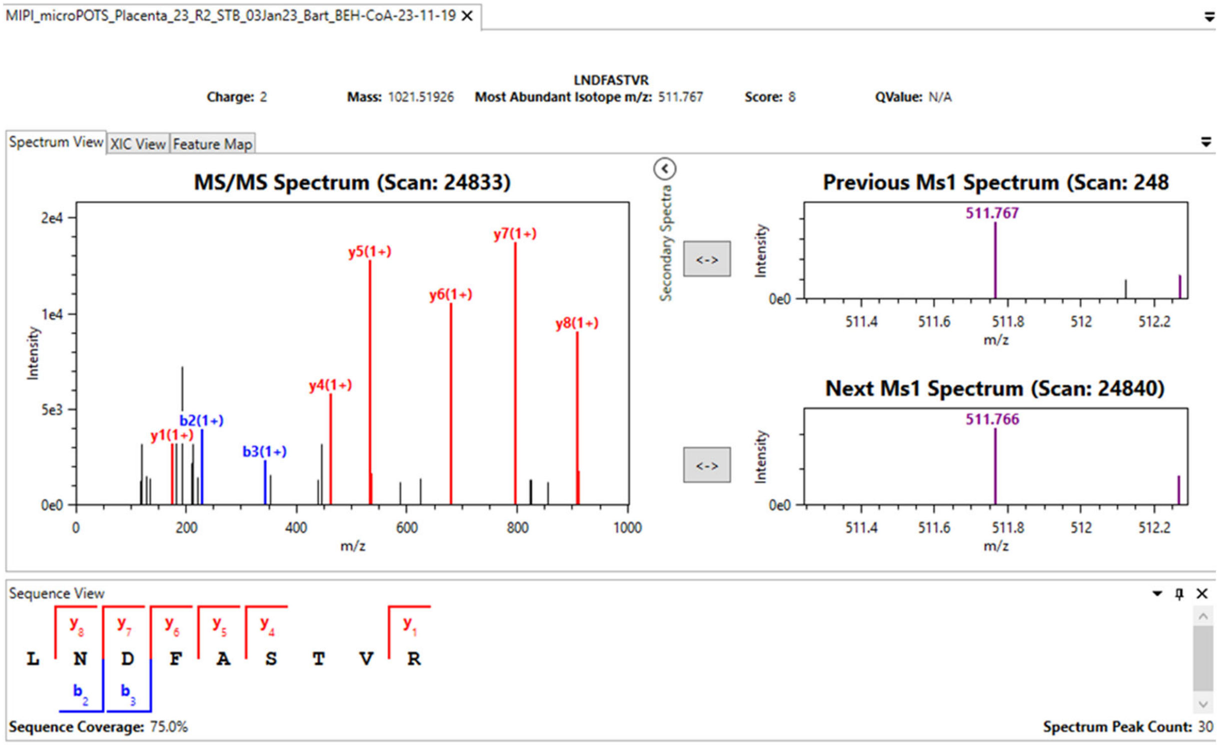

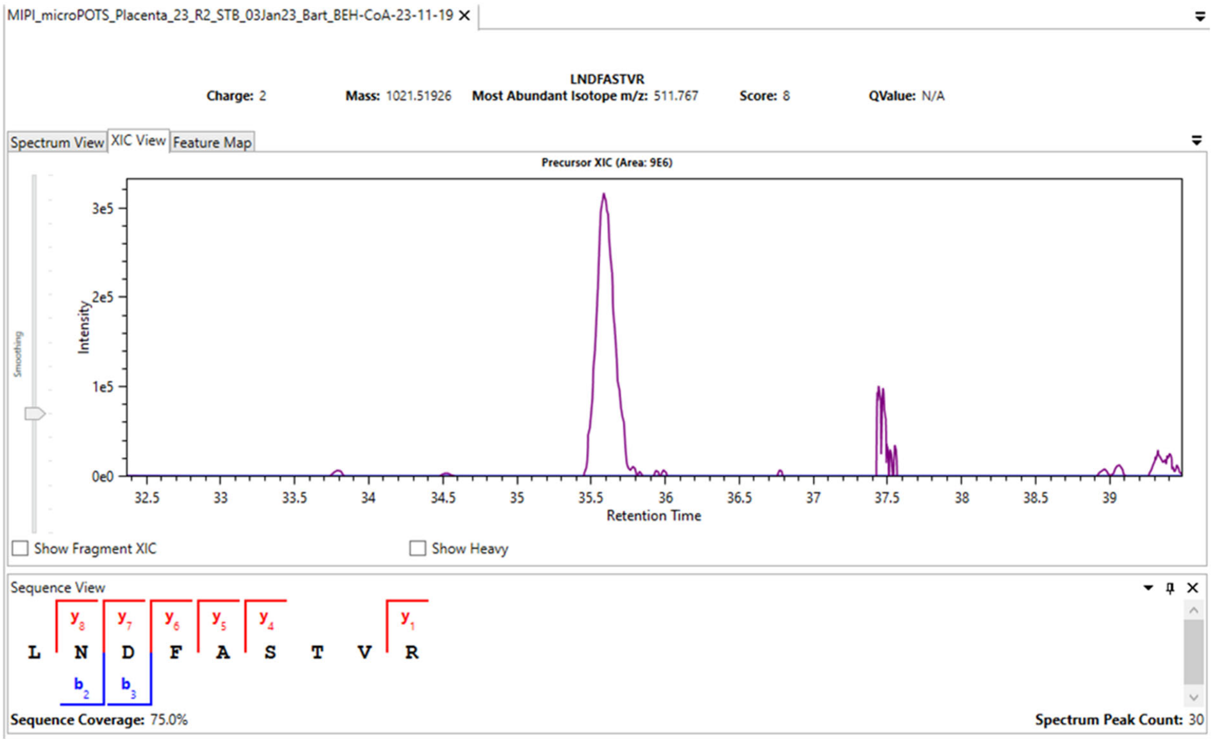

v) Dataset: MIPI_microPOTS_Placenta_26_R3_Core_03Jan23_Bart_BEH-CoA-23-11-19; Scan Number for MS/MS: 24871

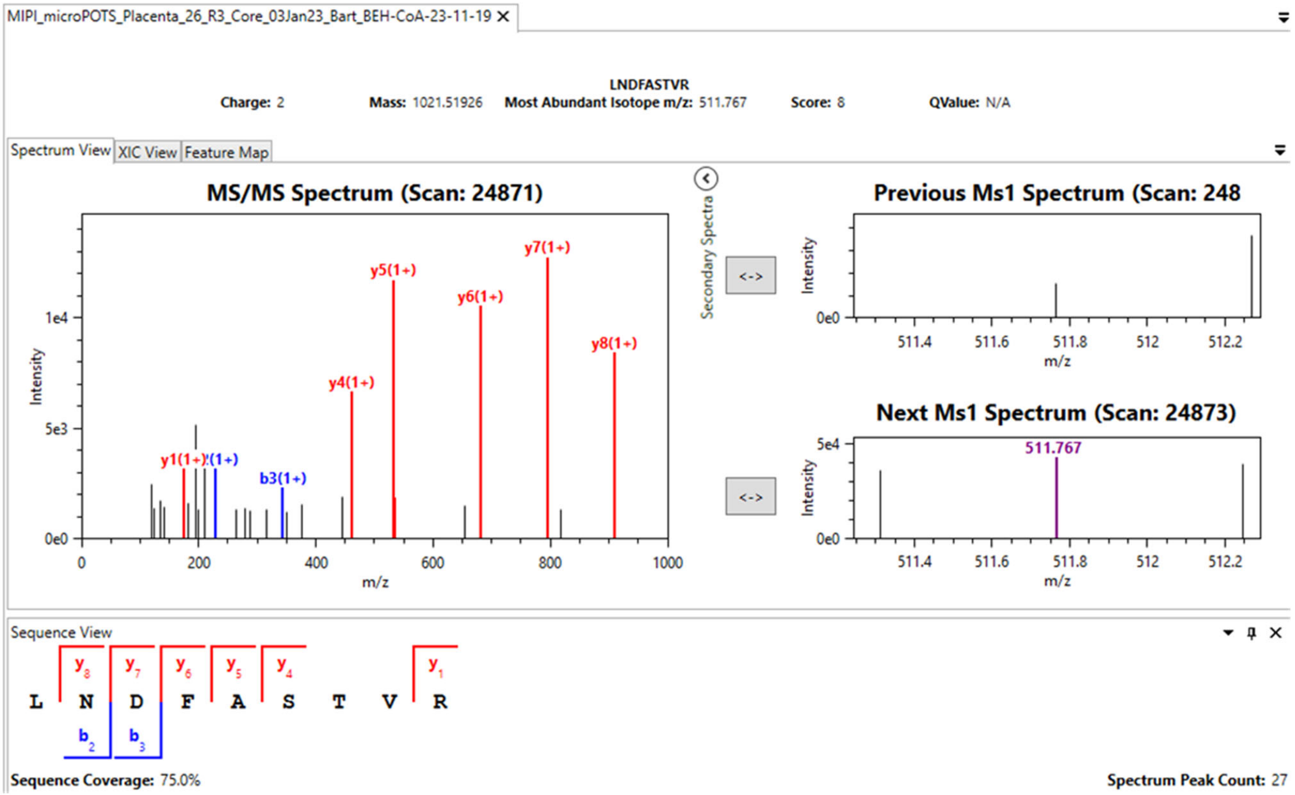

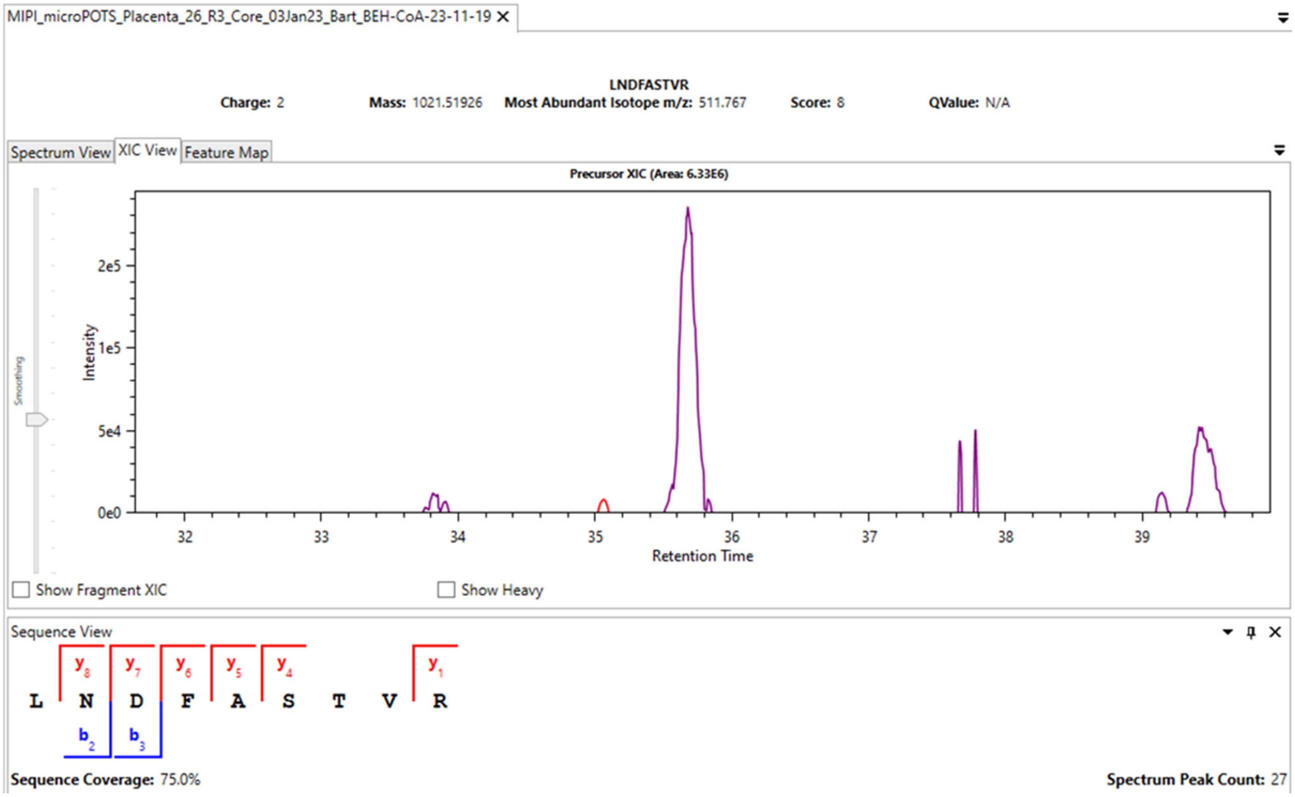

vi) Dataset: MIPI_microPOTS_Placenta_23_R3_Core_03Jan23_Bart_BEH-CoA-23-11-19; Scan Number for MS/MS: 24969.

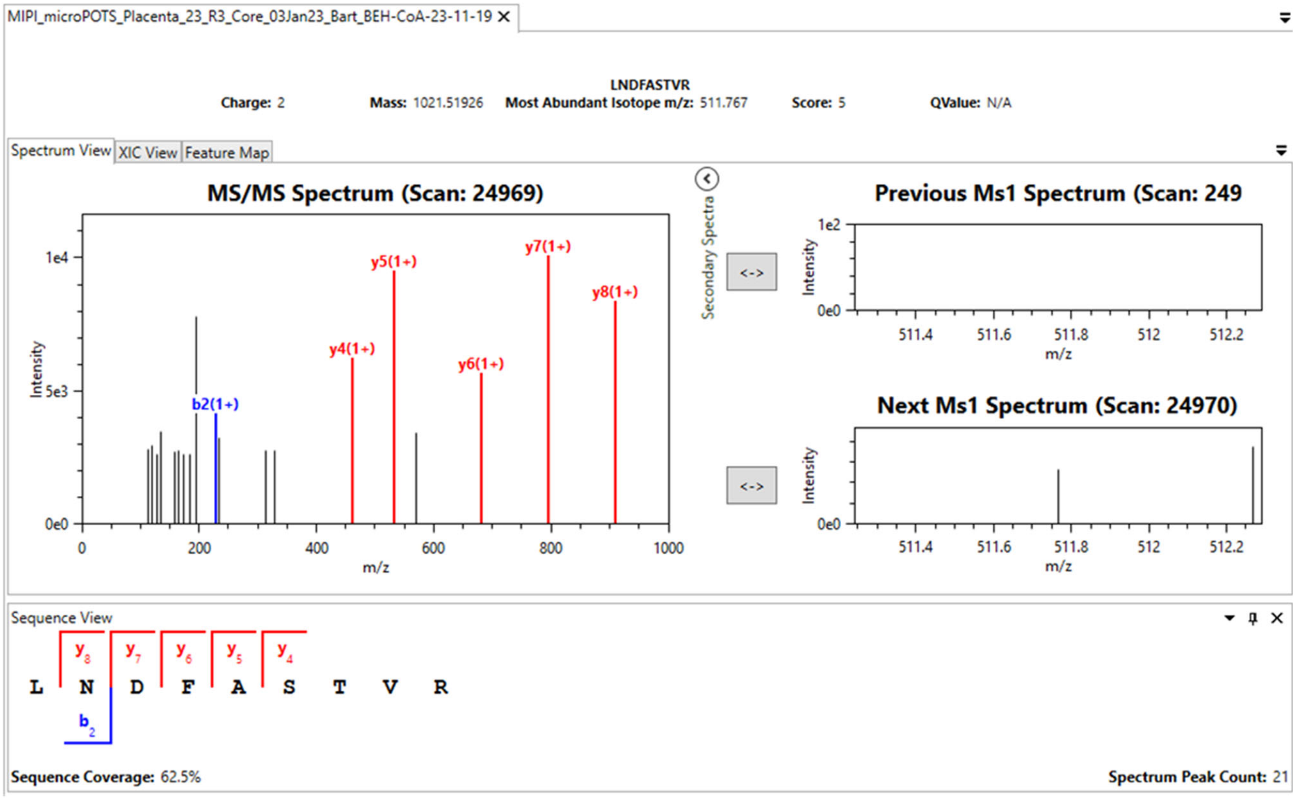

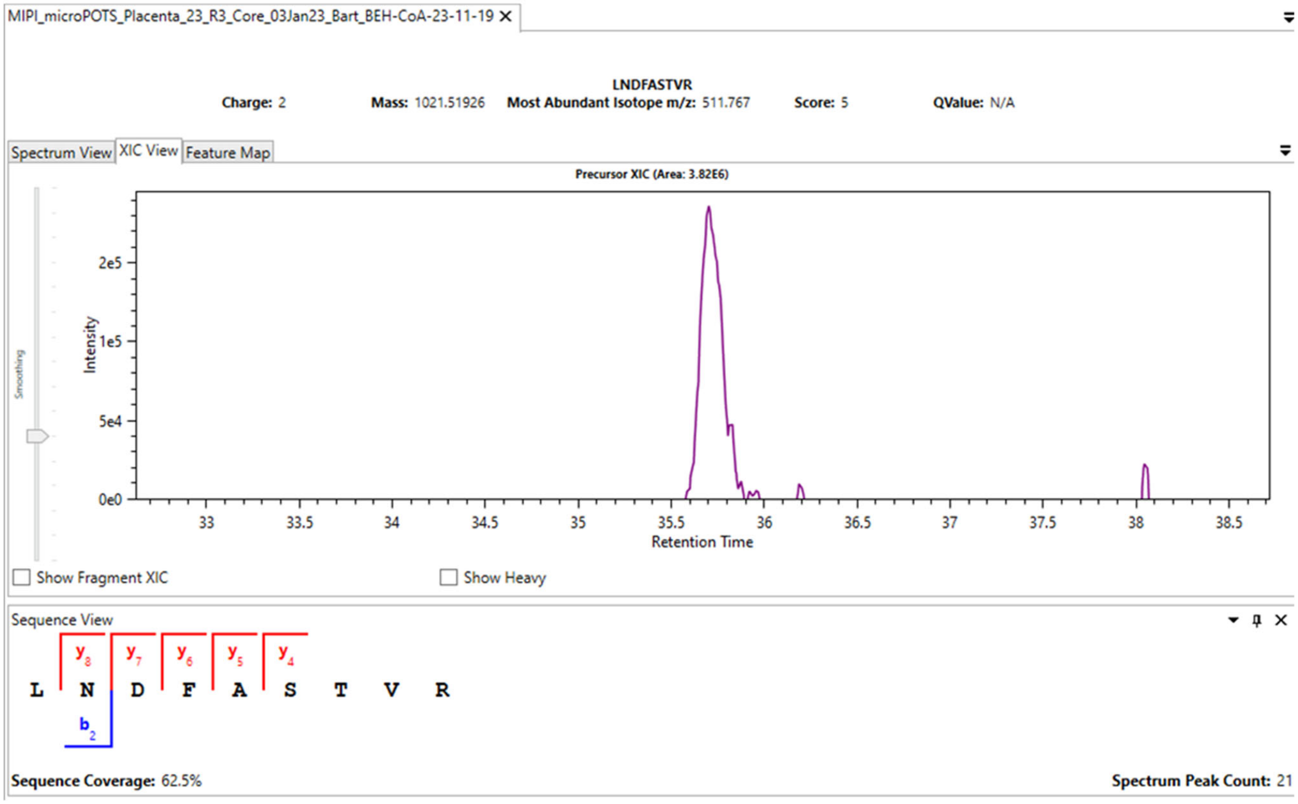

vii) Dataset: MIPI_microPOTS_Placenta_23_R5_Core_03Jan23_Bart_BEH-CoA-23-11-19; Scan Number for MS/MS: 25068.

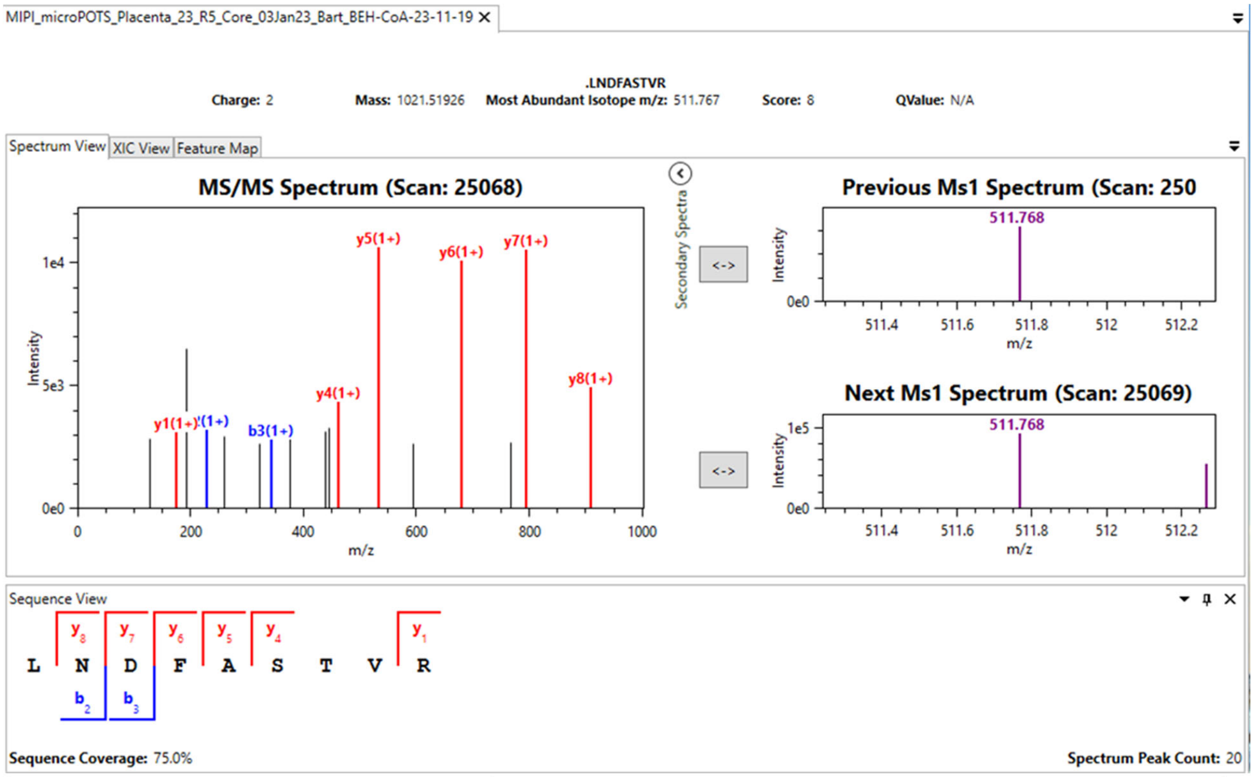

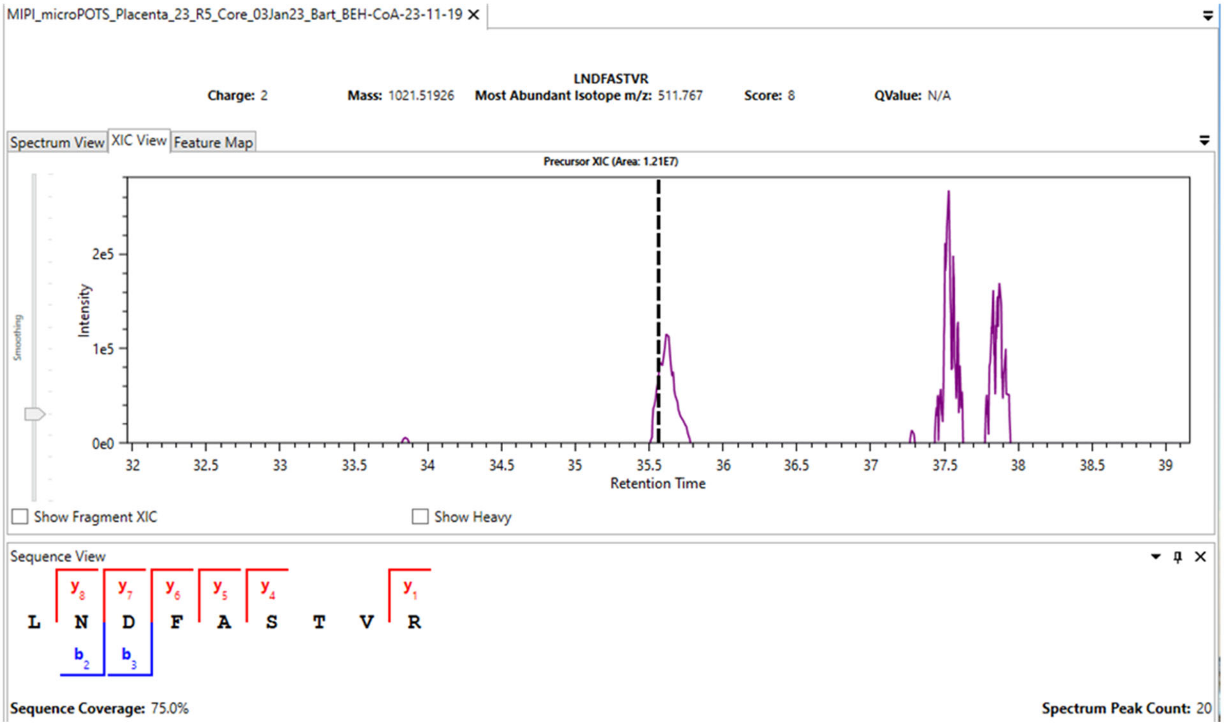

viii) Dataset: MIPI_microPOTS_Placenta_23_R5_STB_03Jan23_Bart_BEH-CoA-23-11-19; Scan Number for MS/MS: 25267

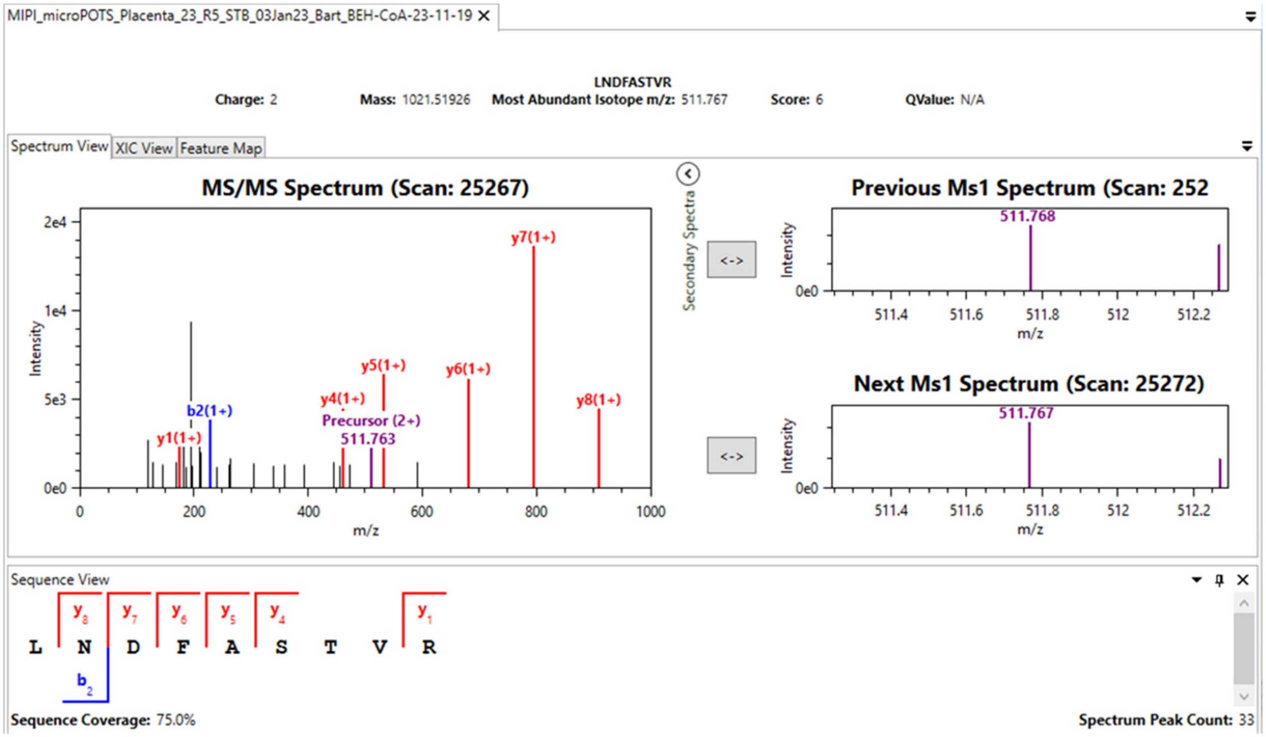

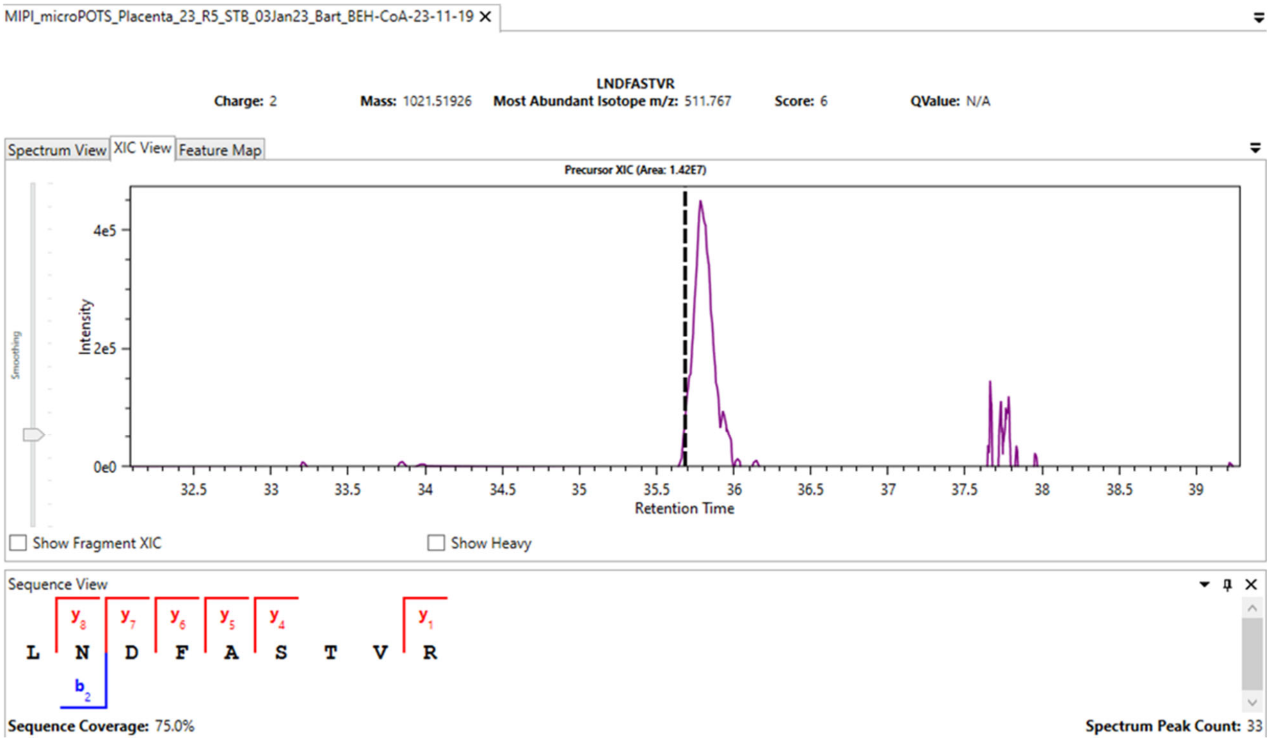

ix) Dataset: MIPI_microPOTS_Placenta_19_R5_STB_03Jan23_Bart_BEH-CoA-23-11-19; Scan Number for MS/MS: 25319

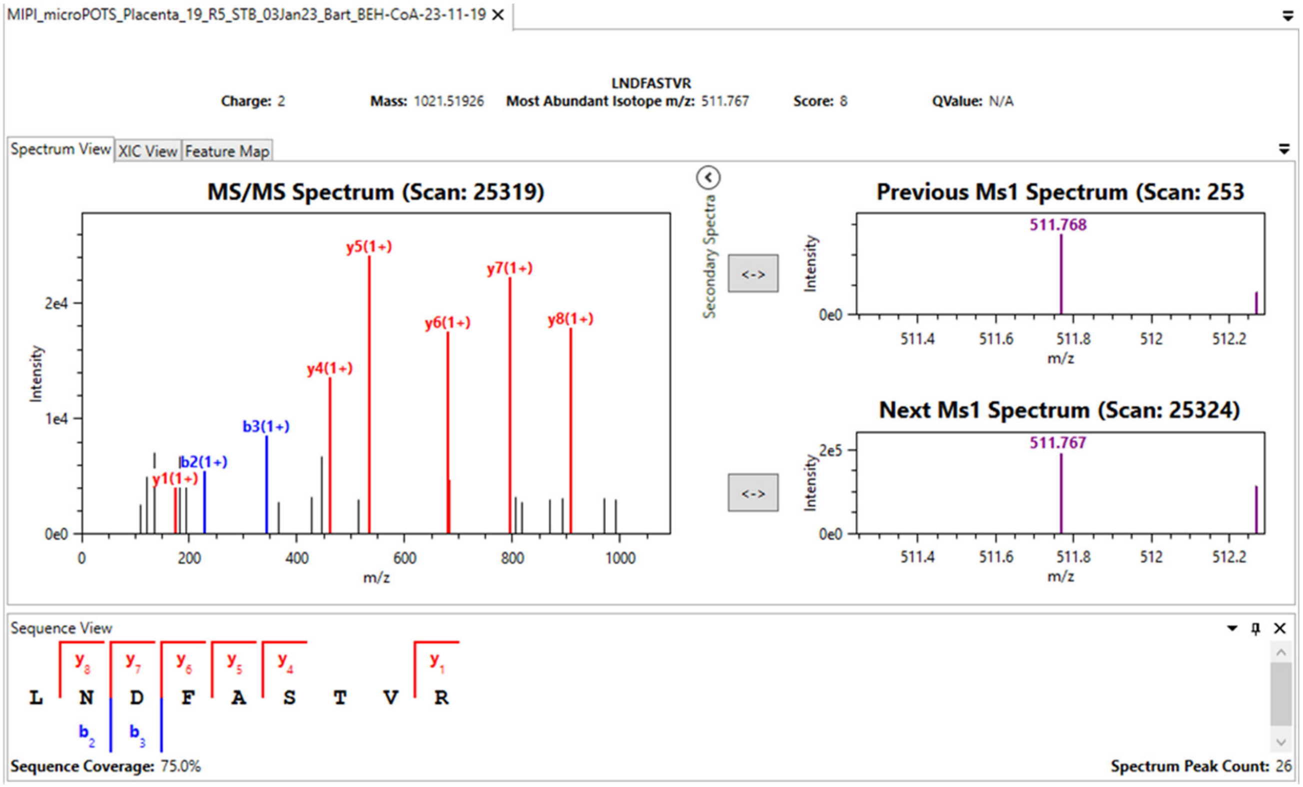

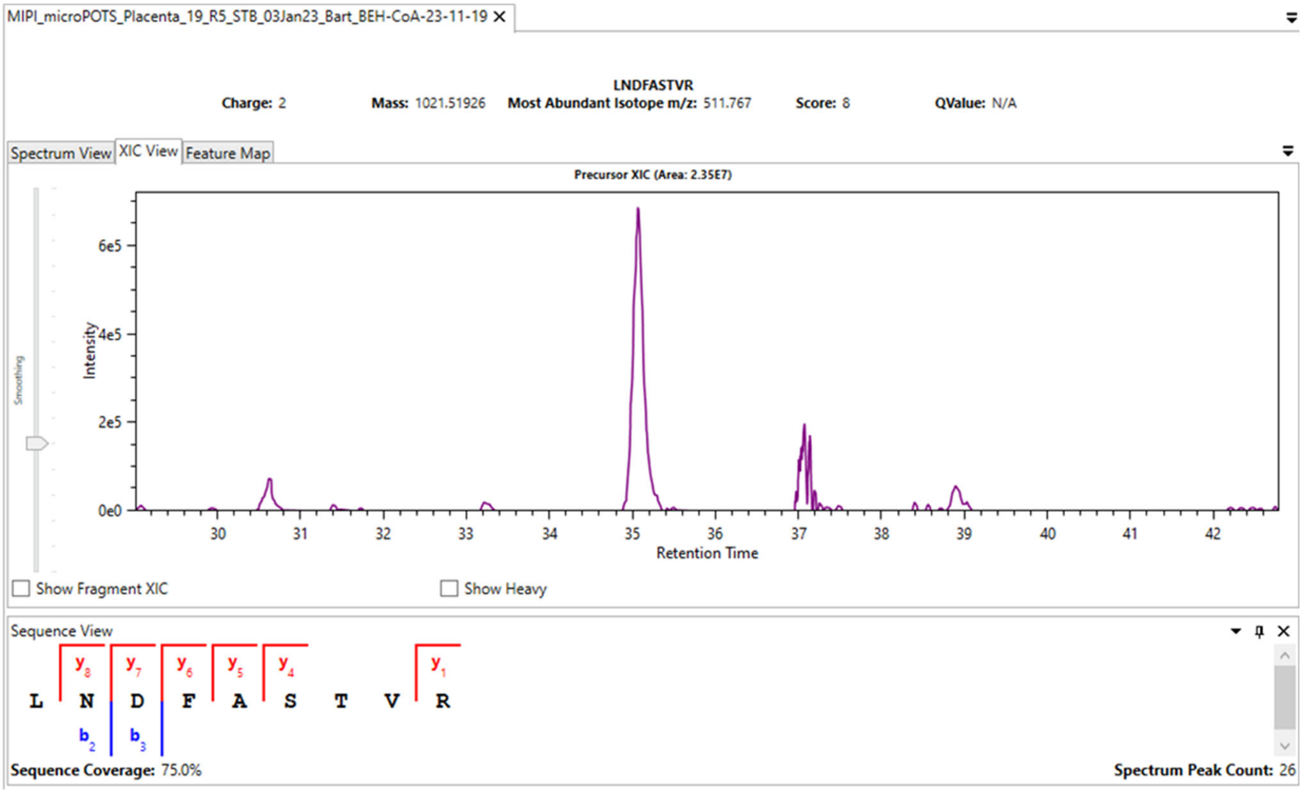

x) Dataset: MIPI_microPOTS_Placenta_26_R4_STB_03Jan23_Bart_BEH-CoA-23-11-19; Scan Number for MS/MS: 25322

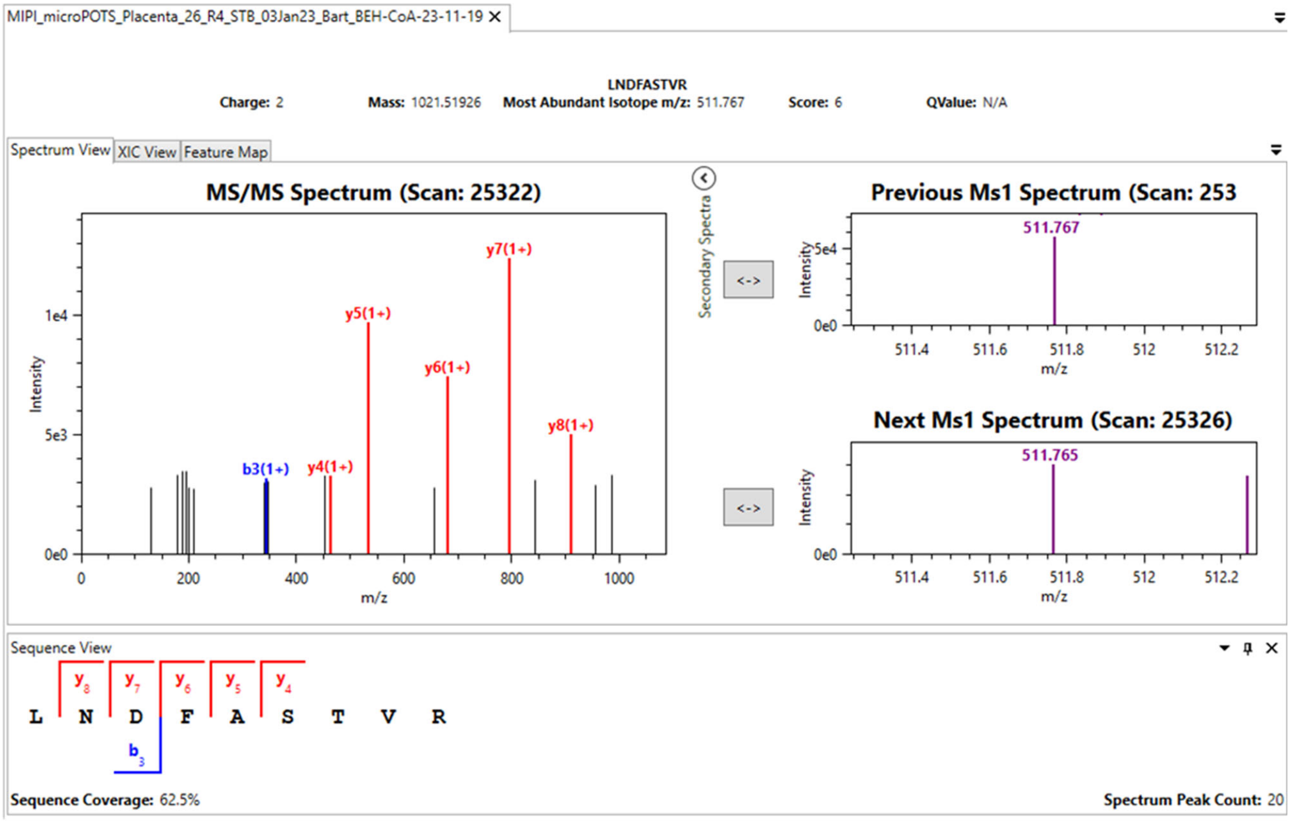

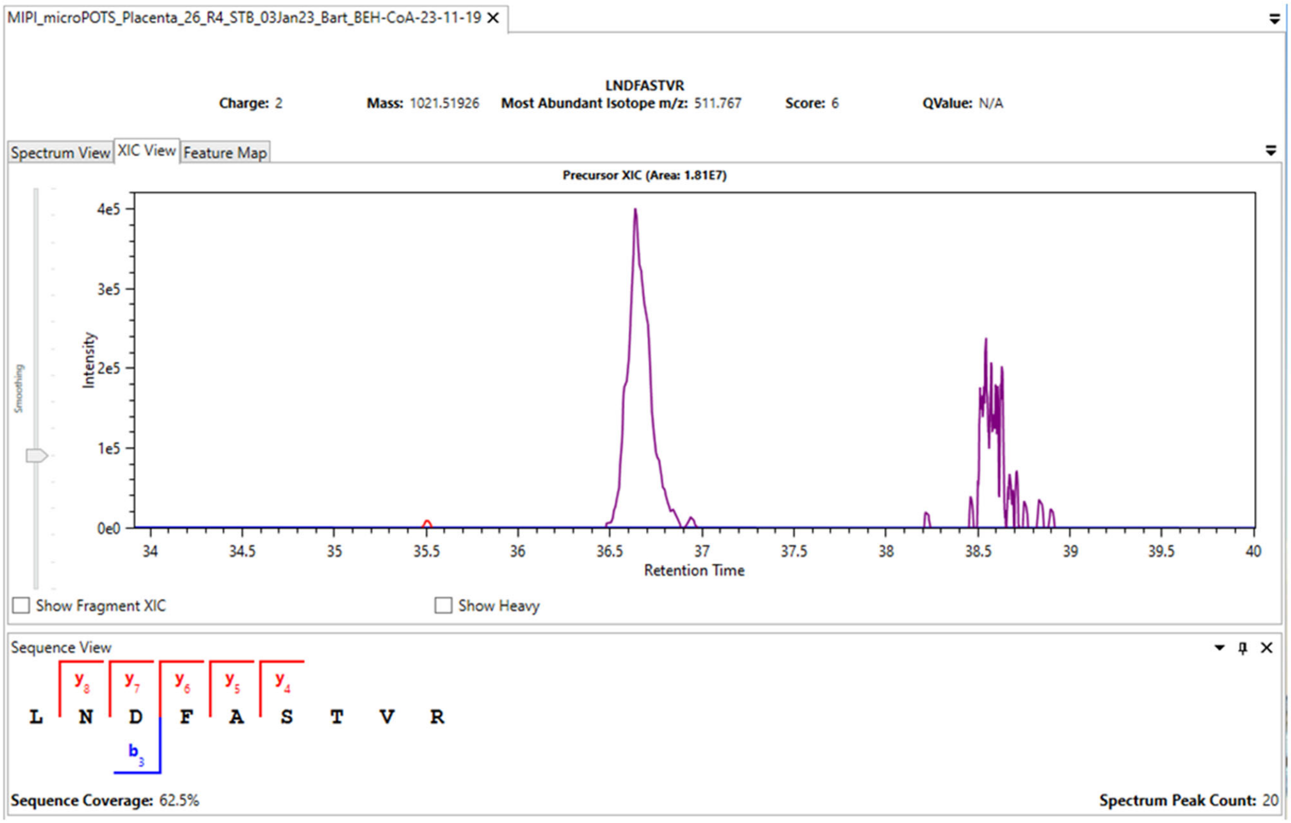

xi) Dataset: MIPI_microPOTS_Placenta_23_R1_Core_03Jan23_Bart_BEH-CoA-23-11-19; Scan Number for MS/MS: 25545

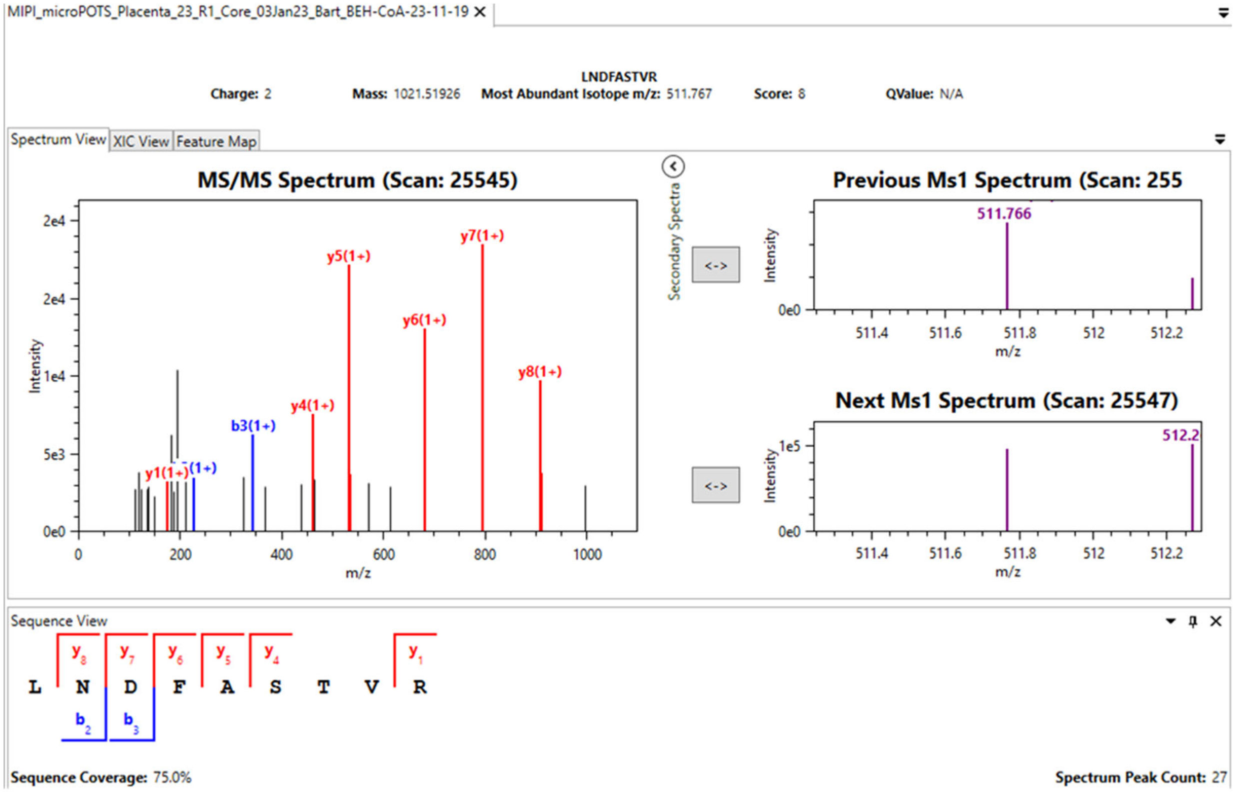

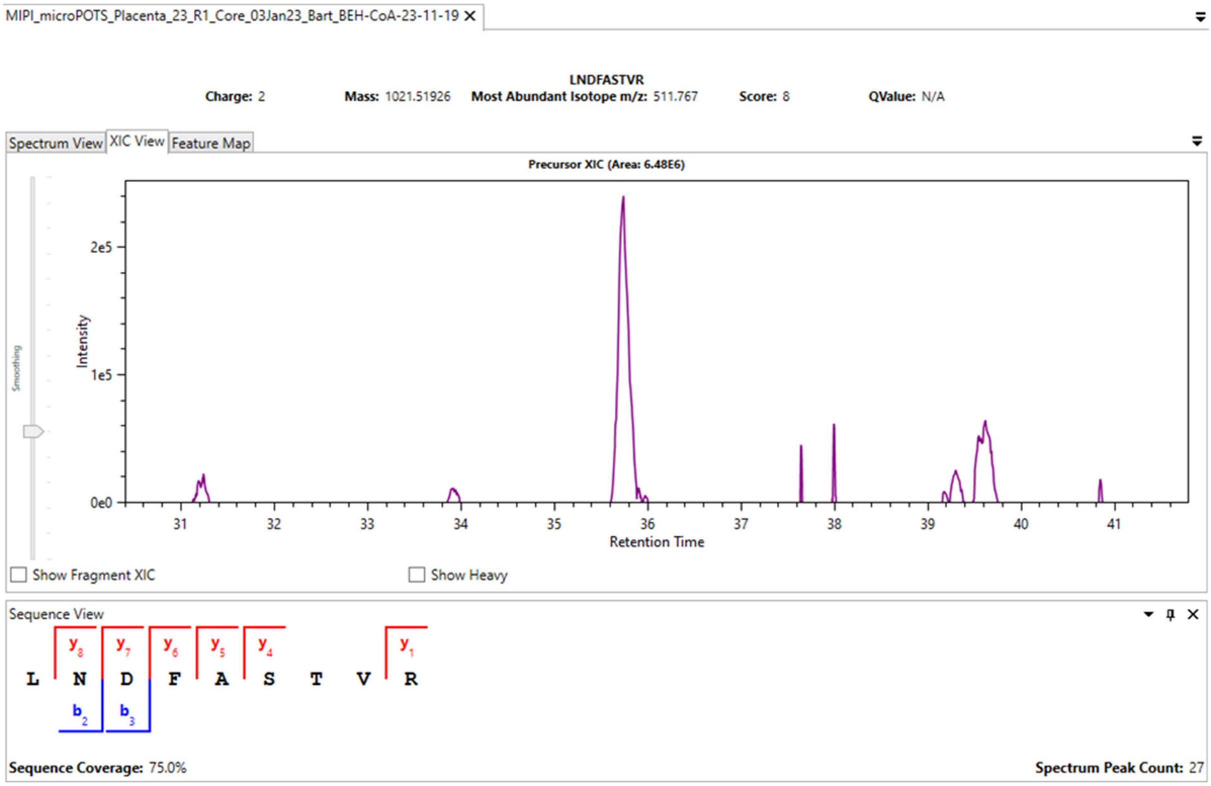

xii) Dataset: MIPI_microPOTS_Placenta_26_R3_STB_03Jan23_Bart_BEH-CoA-23-11-19; Scan Number for MS/MS: 25553

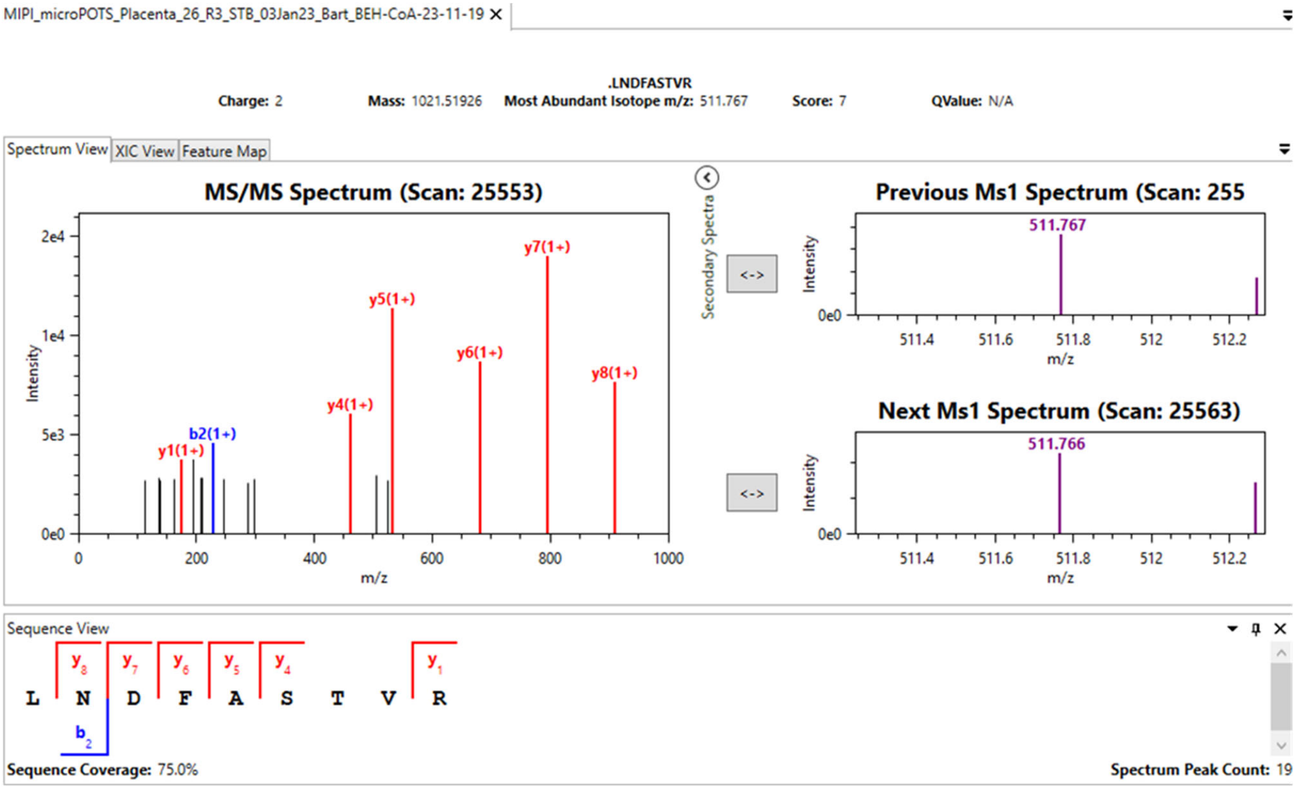

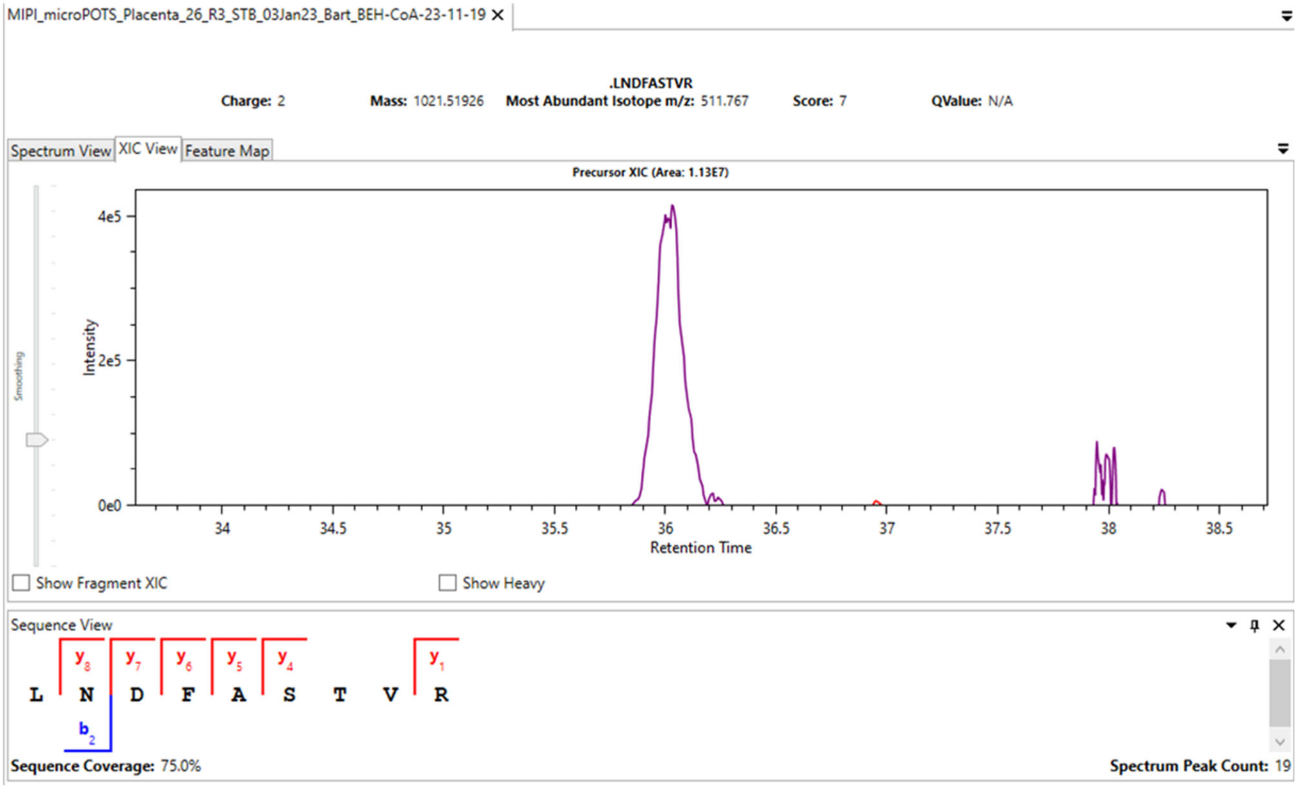

xiii) Dataset: MIPI_microPOTS_Placenta_26_R5_Core_03Jan23_Bart_BEH-CoA-23-11-19; Scan Number for MS/MS: 25606

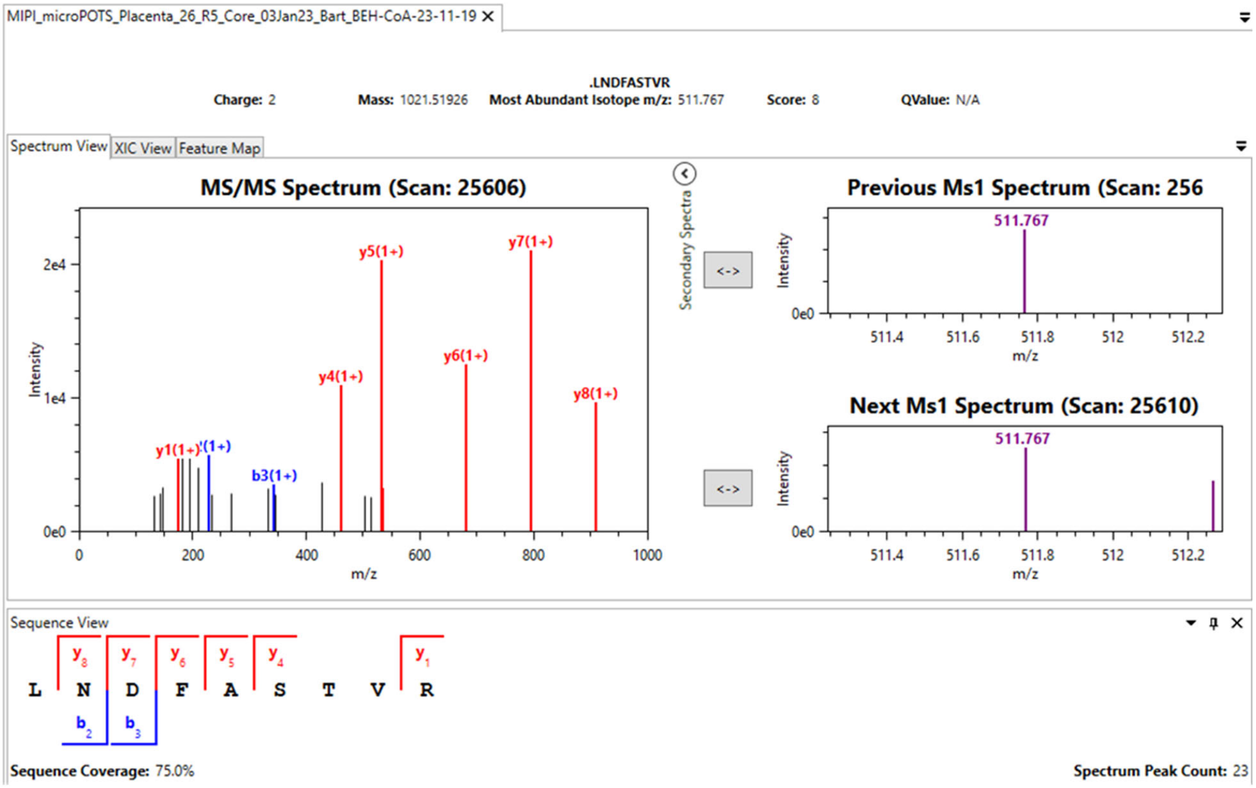

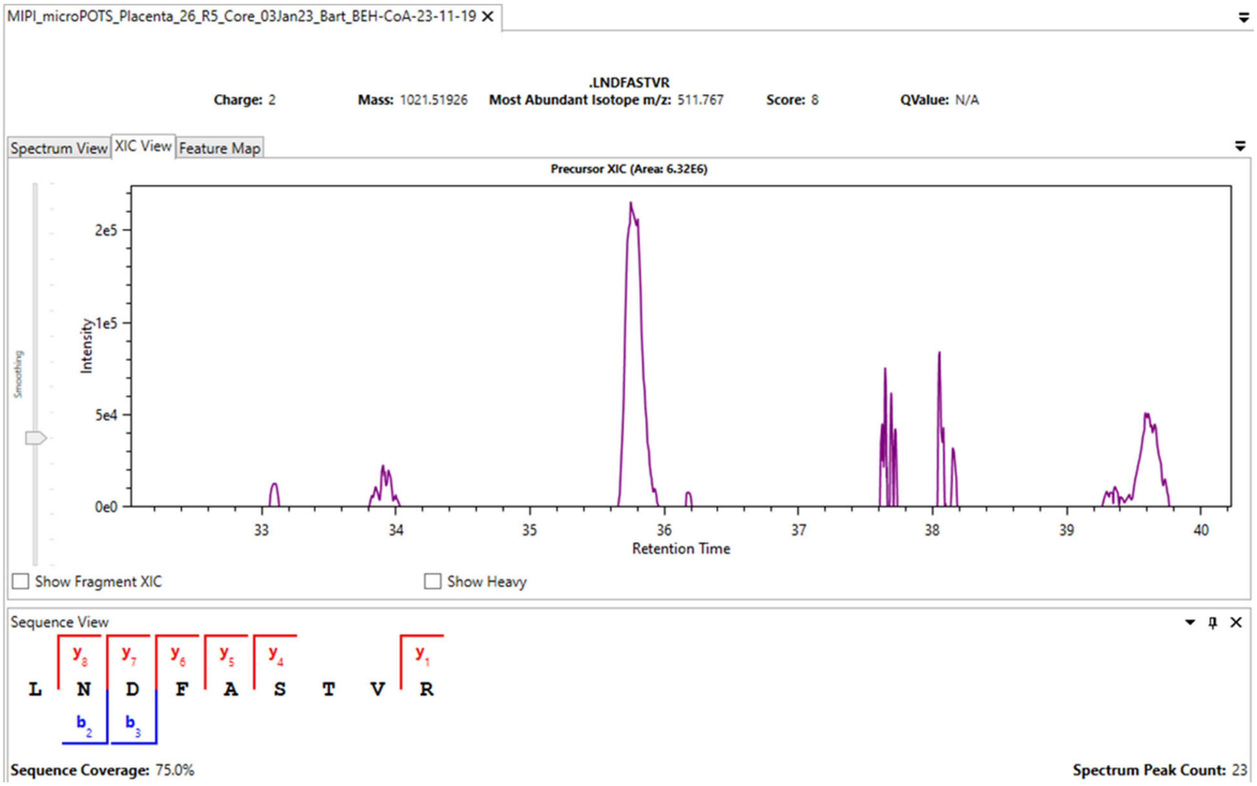

xiv) Dataset: MIPI_microPOTS_Placenta_26_R2_STB_03Jan23_Bart_BEH-CoA-23-11-19; Scan Number for MS/MS: 25674

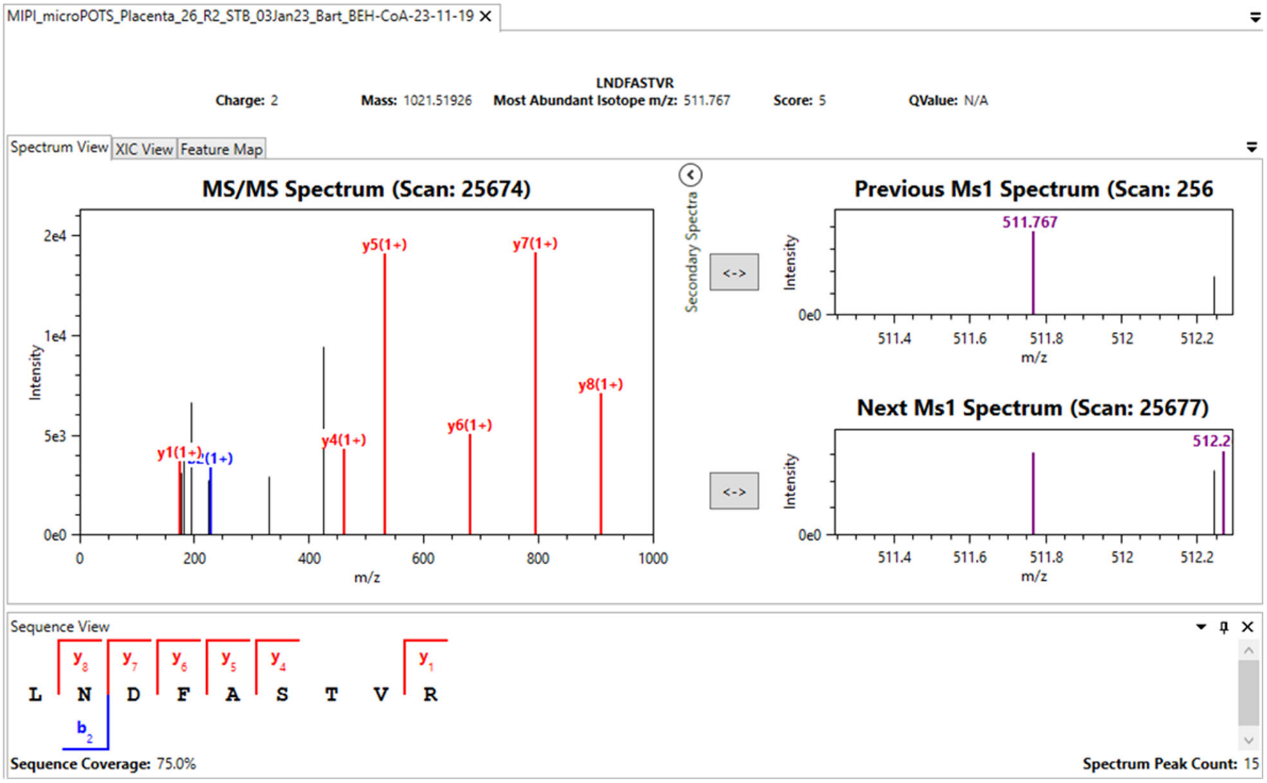

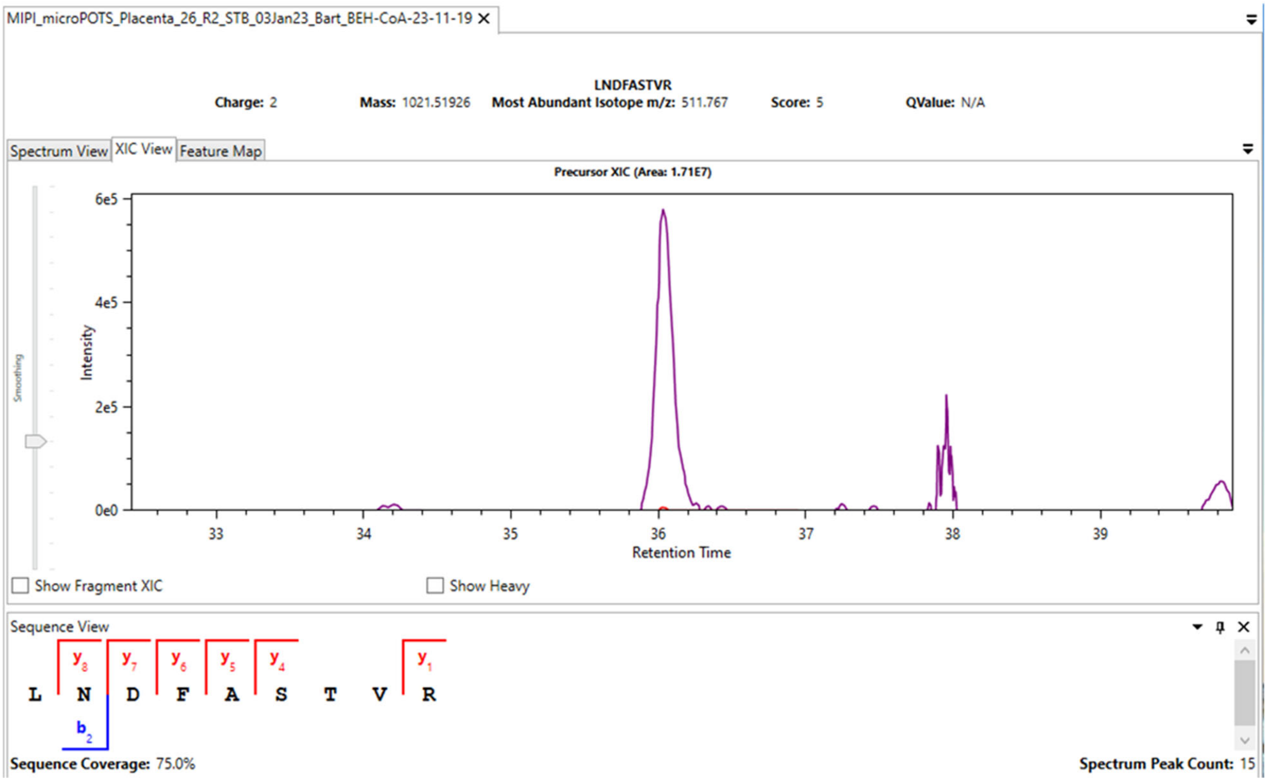

xv) Dataset: MIPI_microPOTS_Placenta_26_R1_Core_03Jan23_Bart_BEH-CoA-23-11-19; Scan Number for MS/MS: 25750

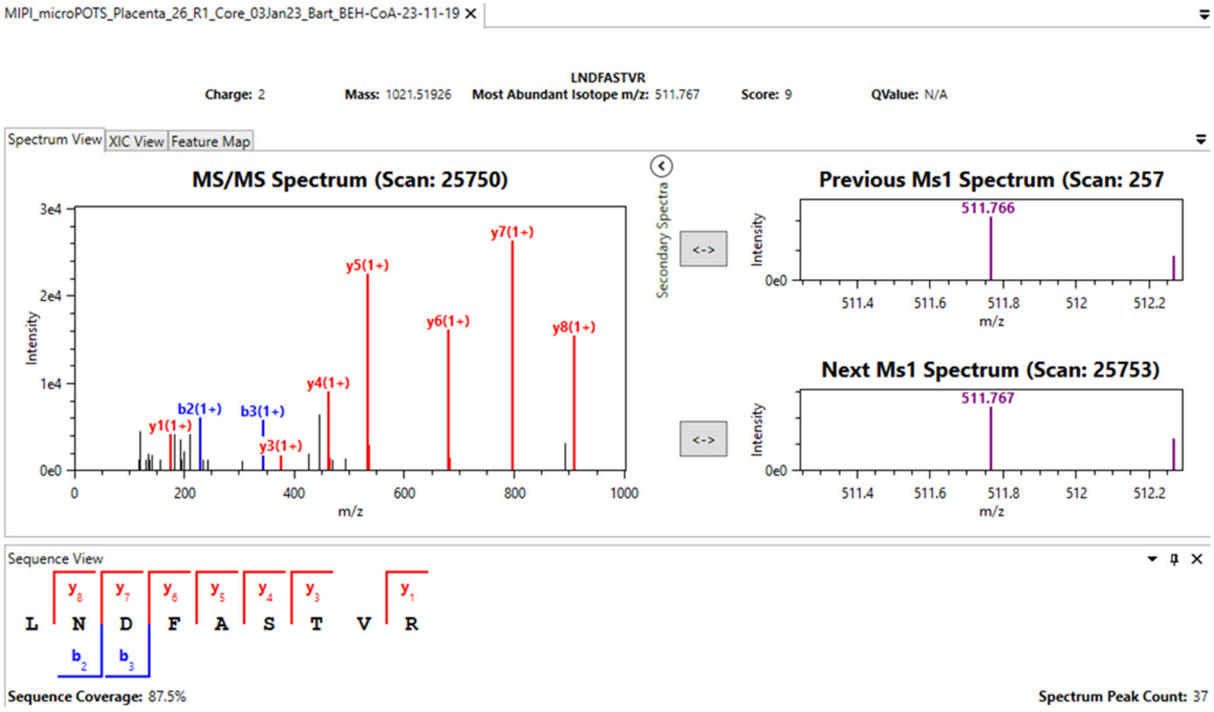

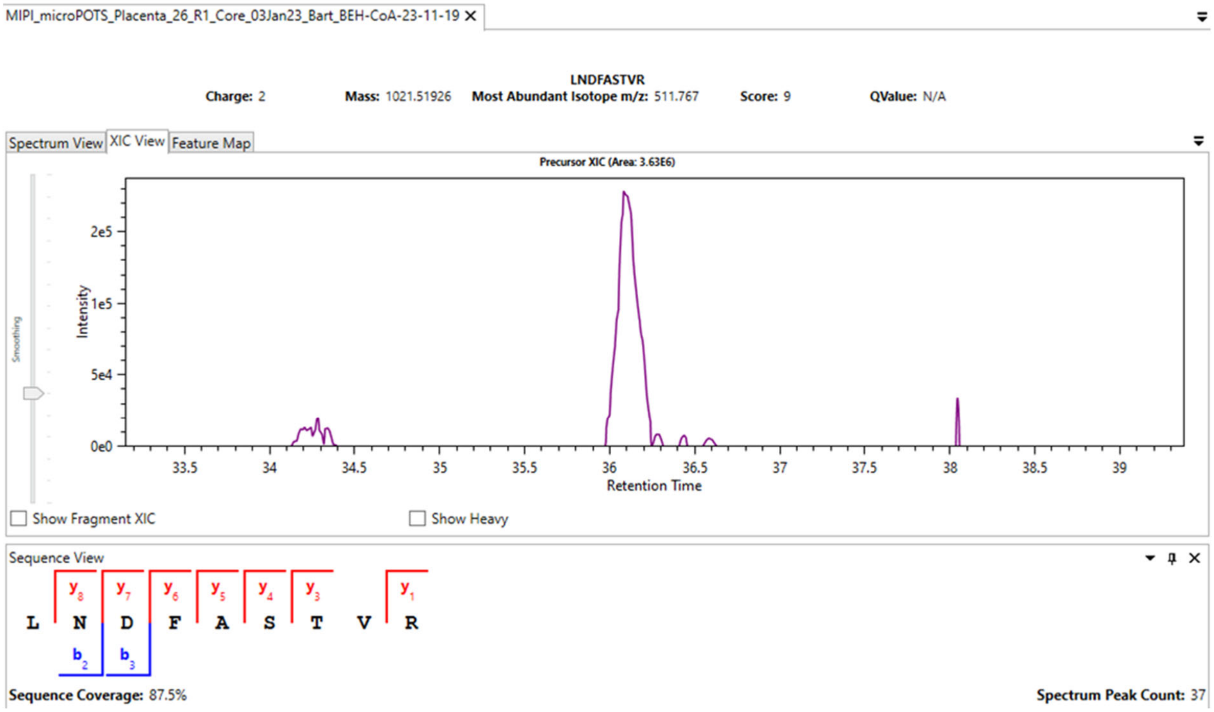

xvi) Dataset: MIPI_microPOTS_Placenta_26_R4_Core_03Jan23_Bart_BEH-CoA-23-11-19; Scan Number for MS/MS: 25864

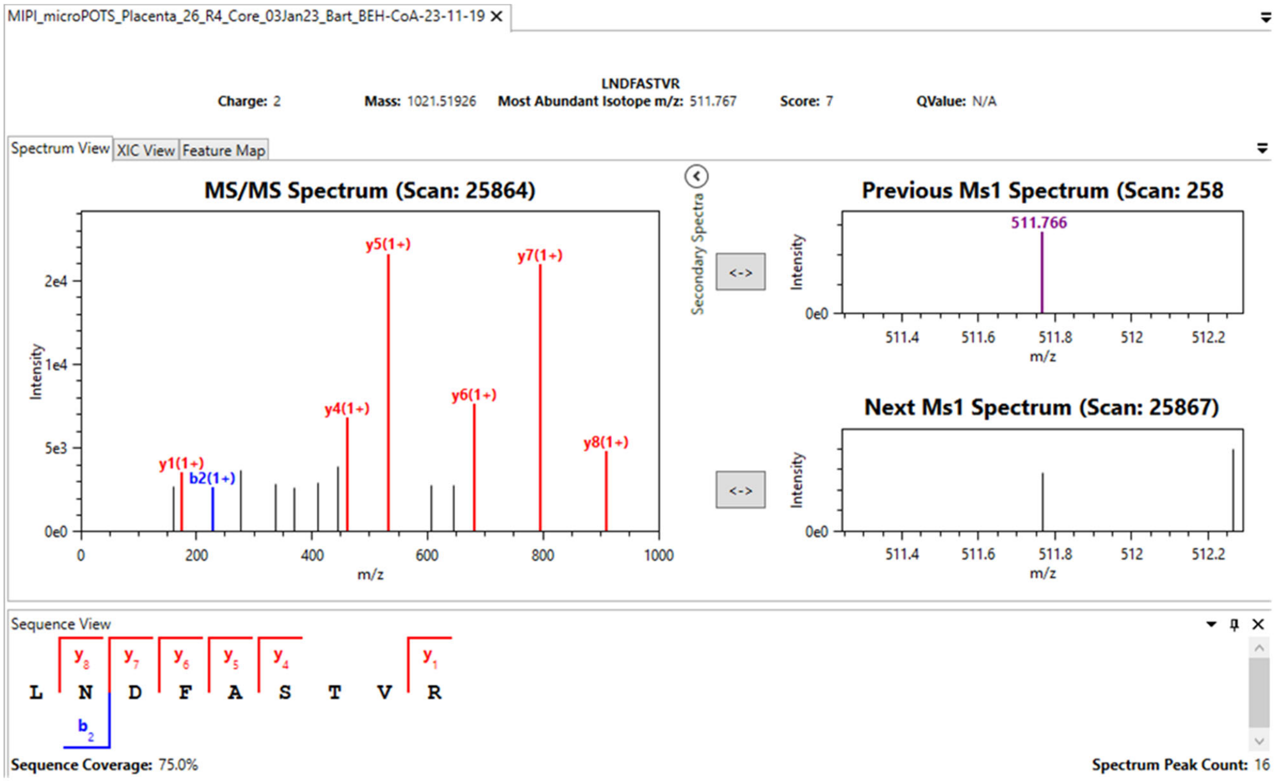

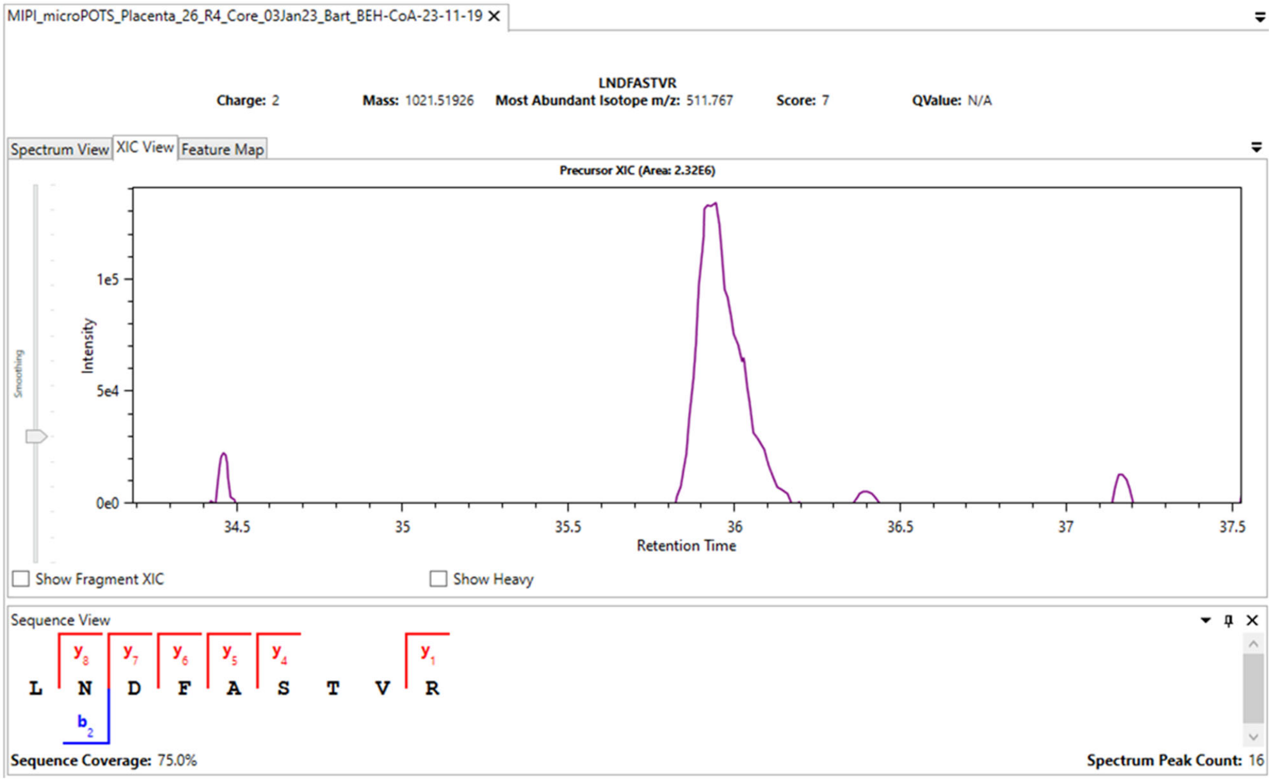

xvii) Dataset: MIPI_microPOTS_Placenta_23_R4_STB_03Jan23_Bart_BEH-CoA-23-11-19; Scan Number for MS/MS: 25998

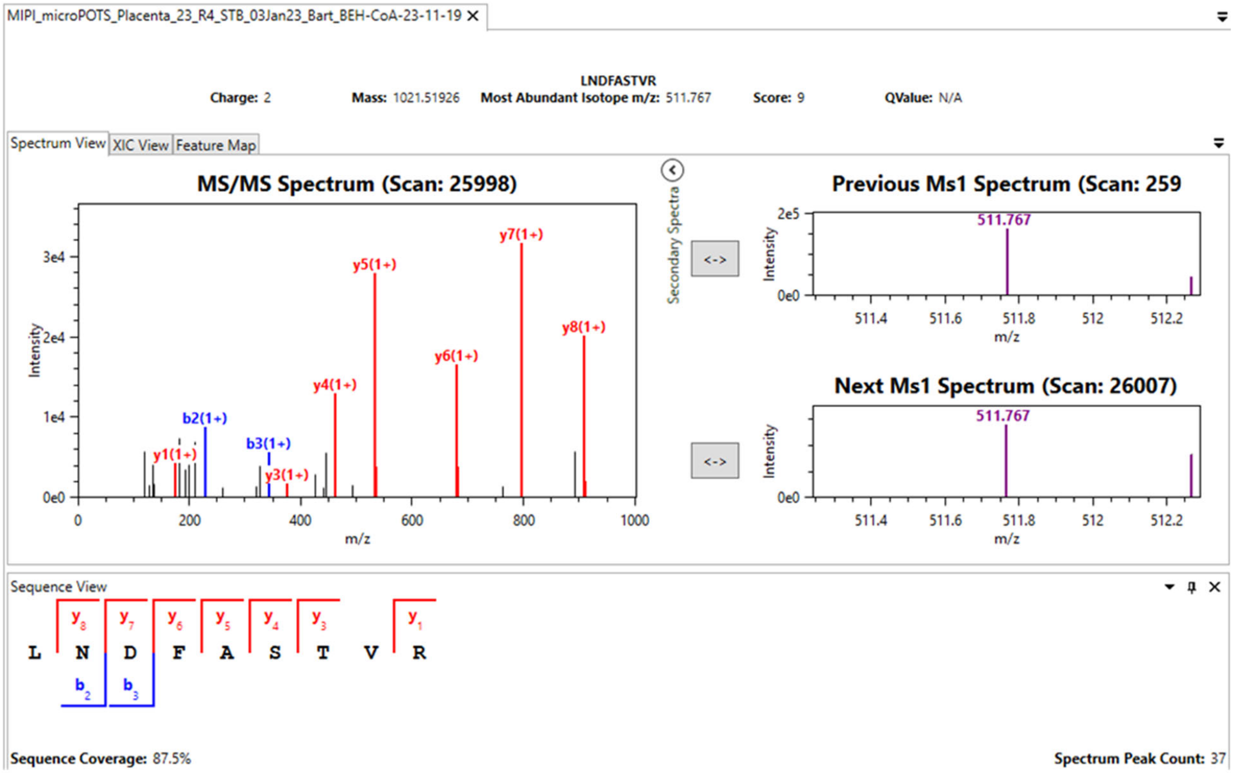

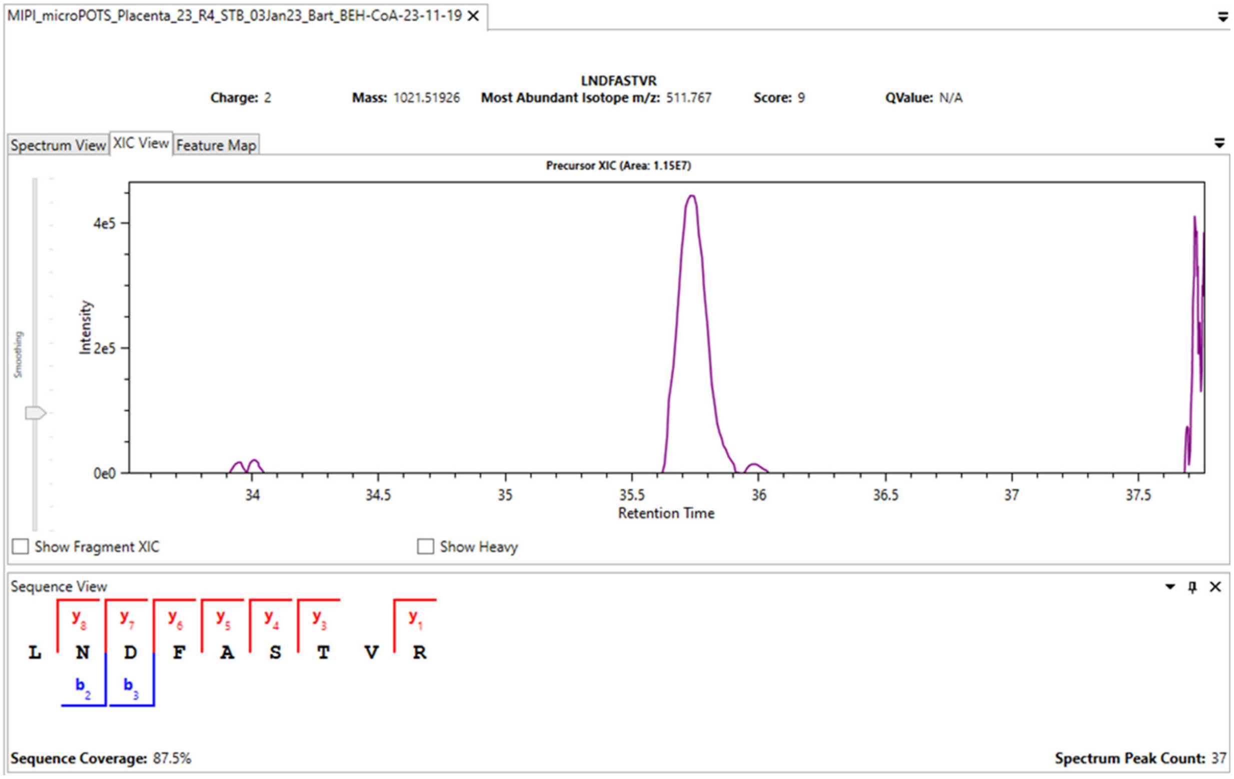

xviii) Dataset: MIPI_microPOTS_Placenta_26_R5_STB_03Jan23_Bart_BEH-CoA-23-11-19; Scan Number for MS/MS: 26019

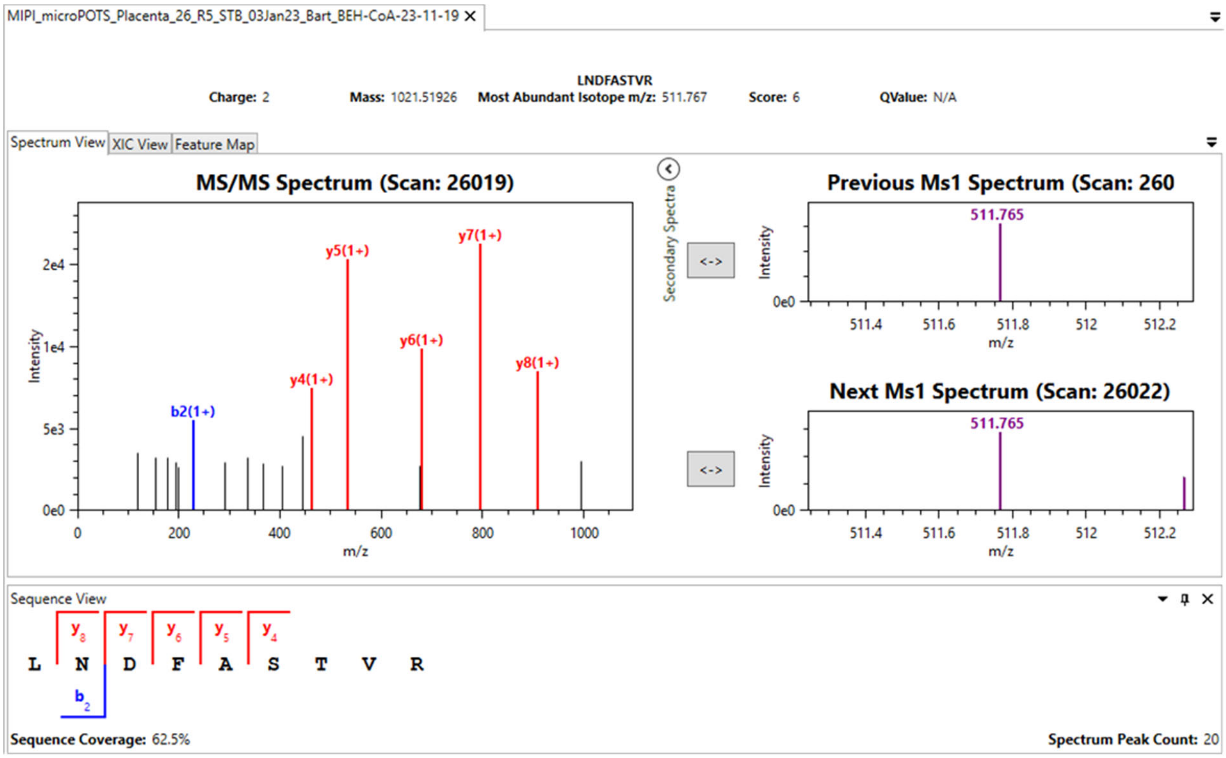

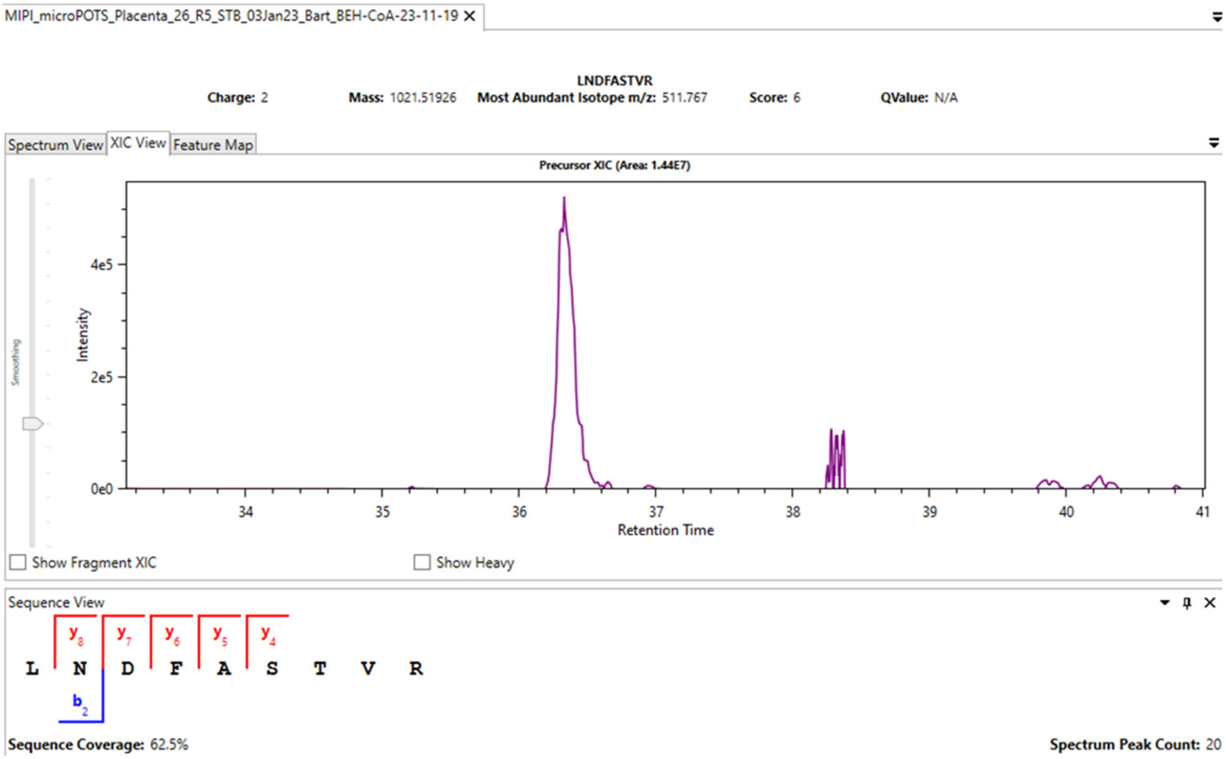

xix) Dataset: MIPI_microPOTS_Placenta_19_R5_Core_03Jan23_Bart_BEH-CoA-23-11-19; Scan Number for MS/MS: 26073

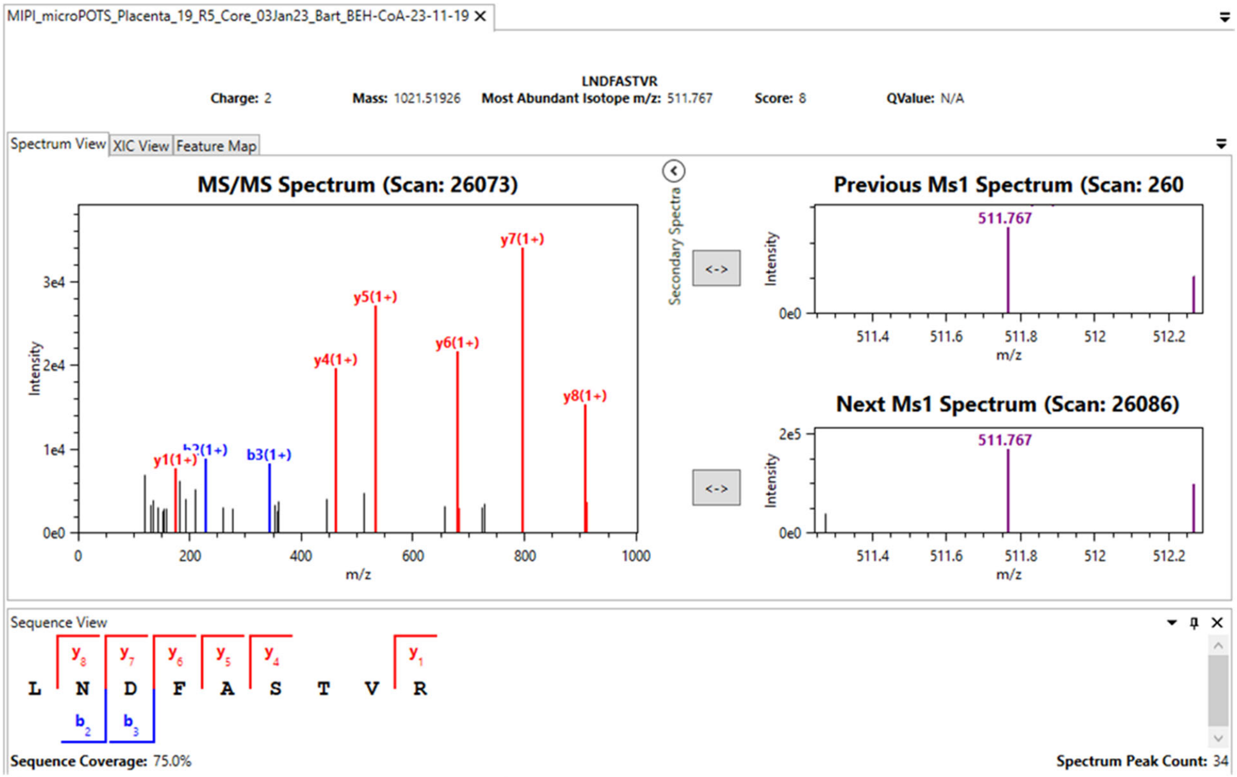

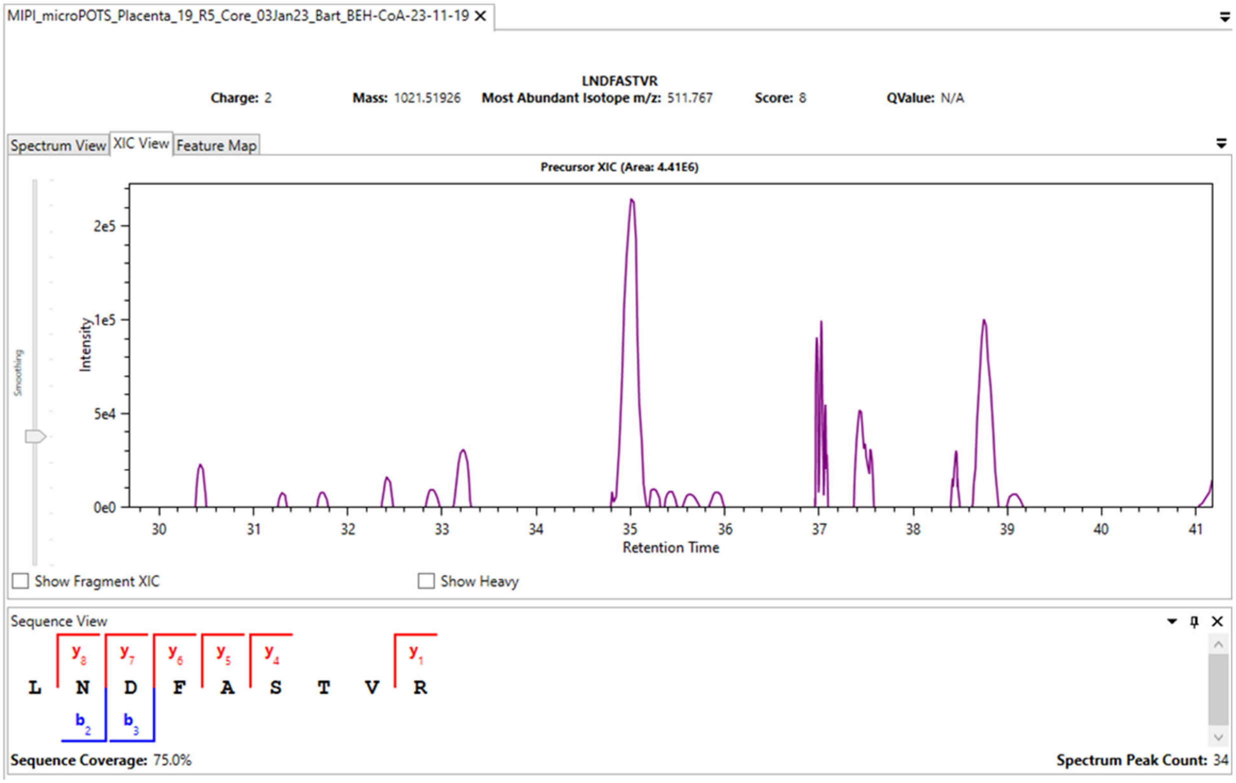

xx) Dataset: MIPI_microPOTS_Placenta_26_R2_Core_03Jan23_Bart_BEH-CoA-23-11-19; Scan Number for MS/MS: 26413

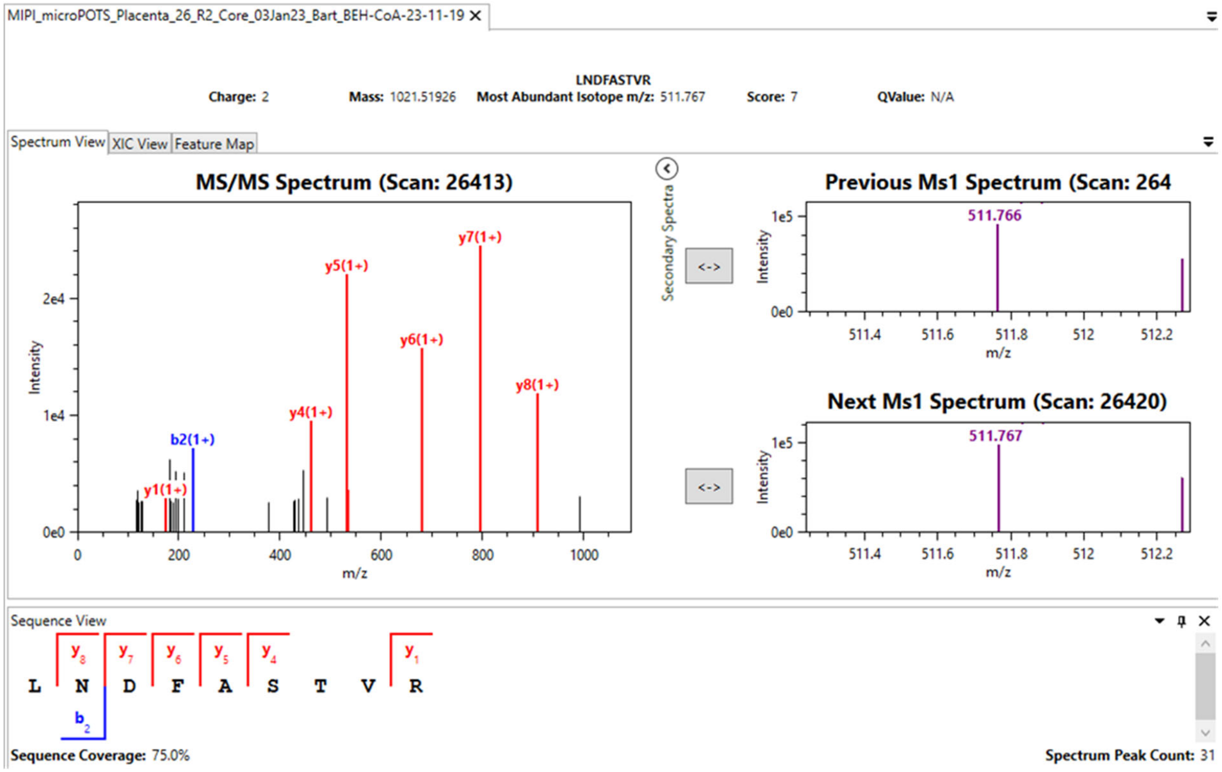

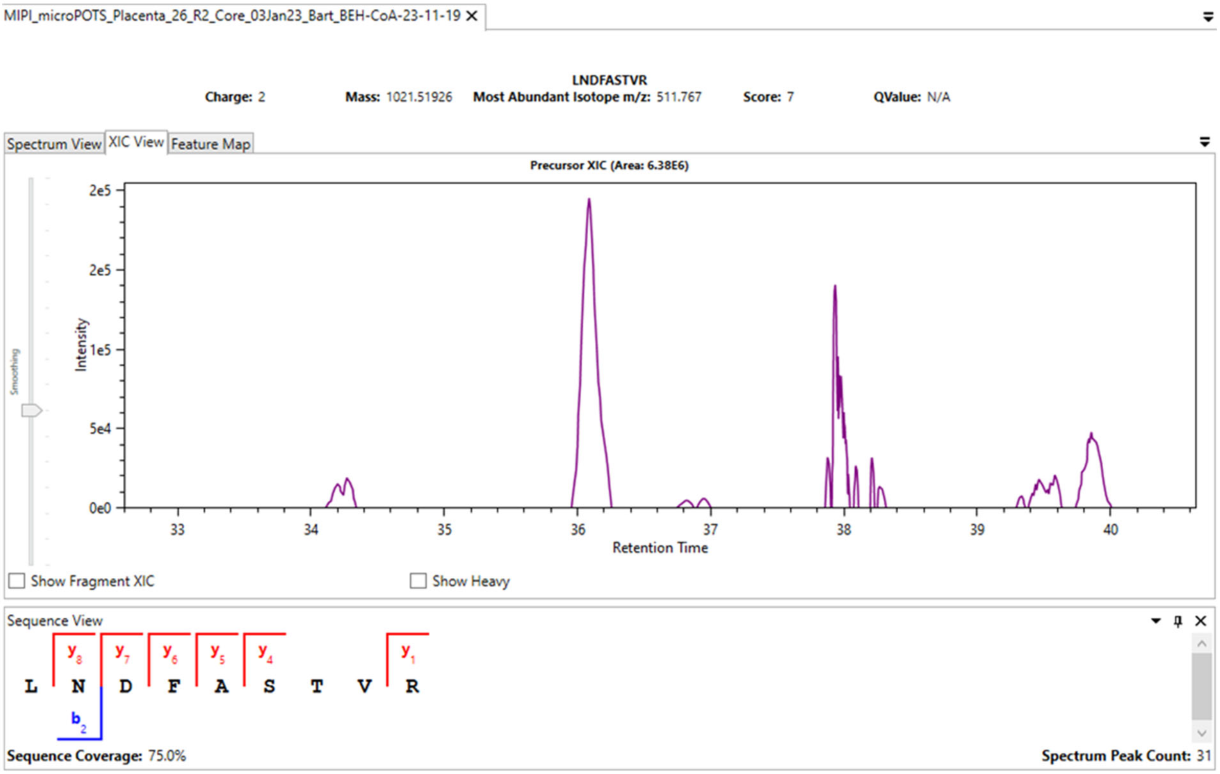

e) Protein: sp|Q9BUT1|DHRS6_HUMAN; Peptide: K.VIILTAAAQGIGQAAALAFAR.E

i) Dataset: MIPI_microPOTS_Placenta_26_R3_Core_03Jan23_Bart_BEH-CoA-23-11-19; Scan Number for MS/MS: 79761

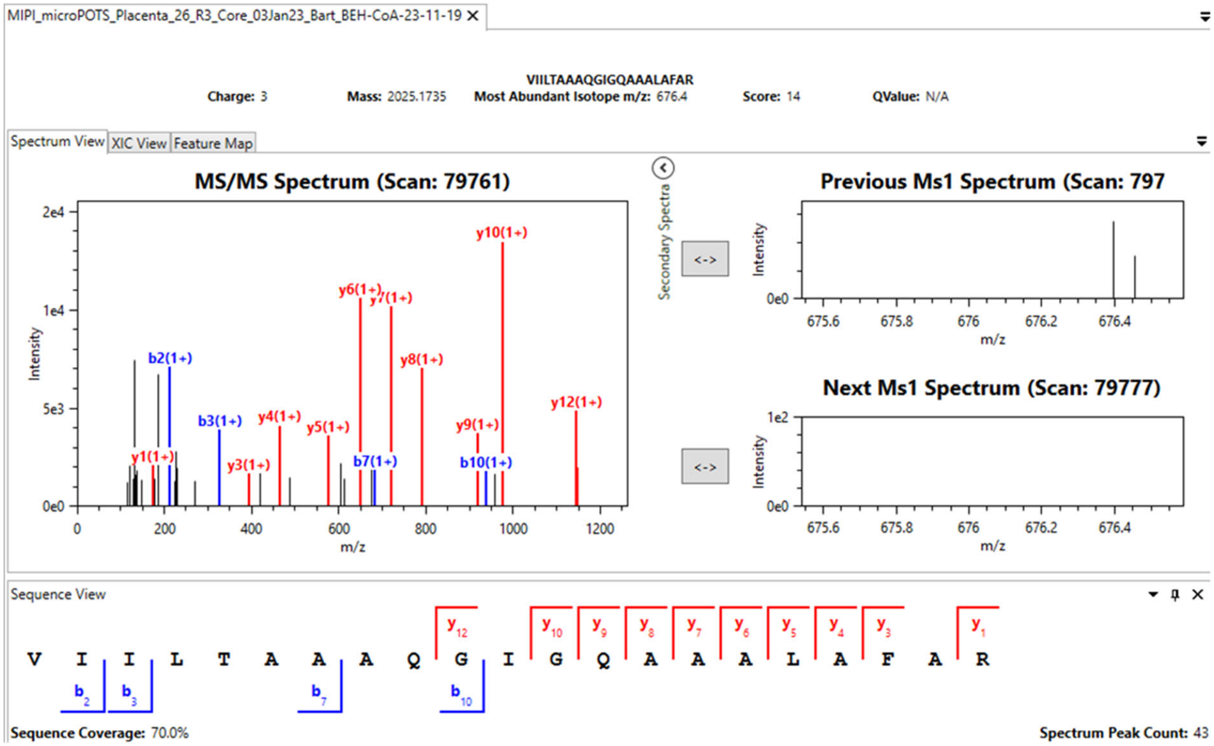

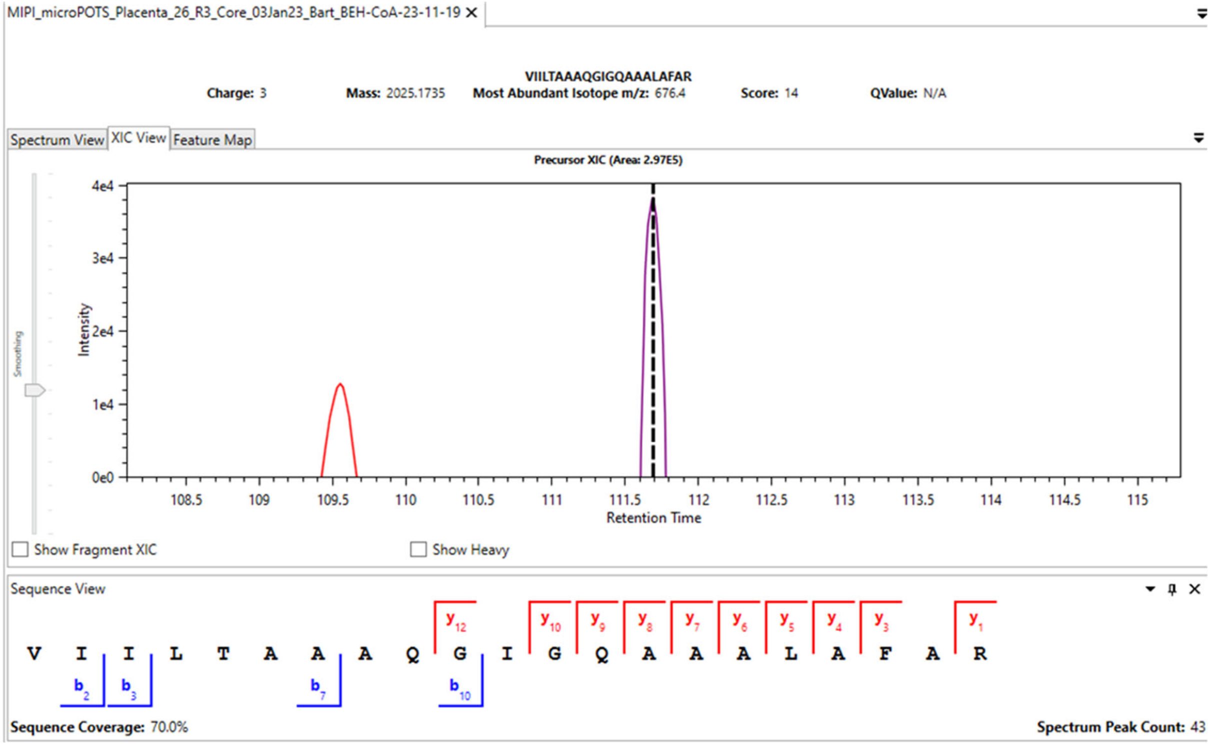

ii) Dataset: MIPI_microPOTS_Placenta_26_R5_Core_03Jan23_Bart_BEH-CoA-23-11-19; Scan Number for MS/MS: 82015

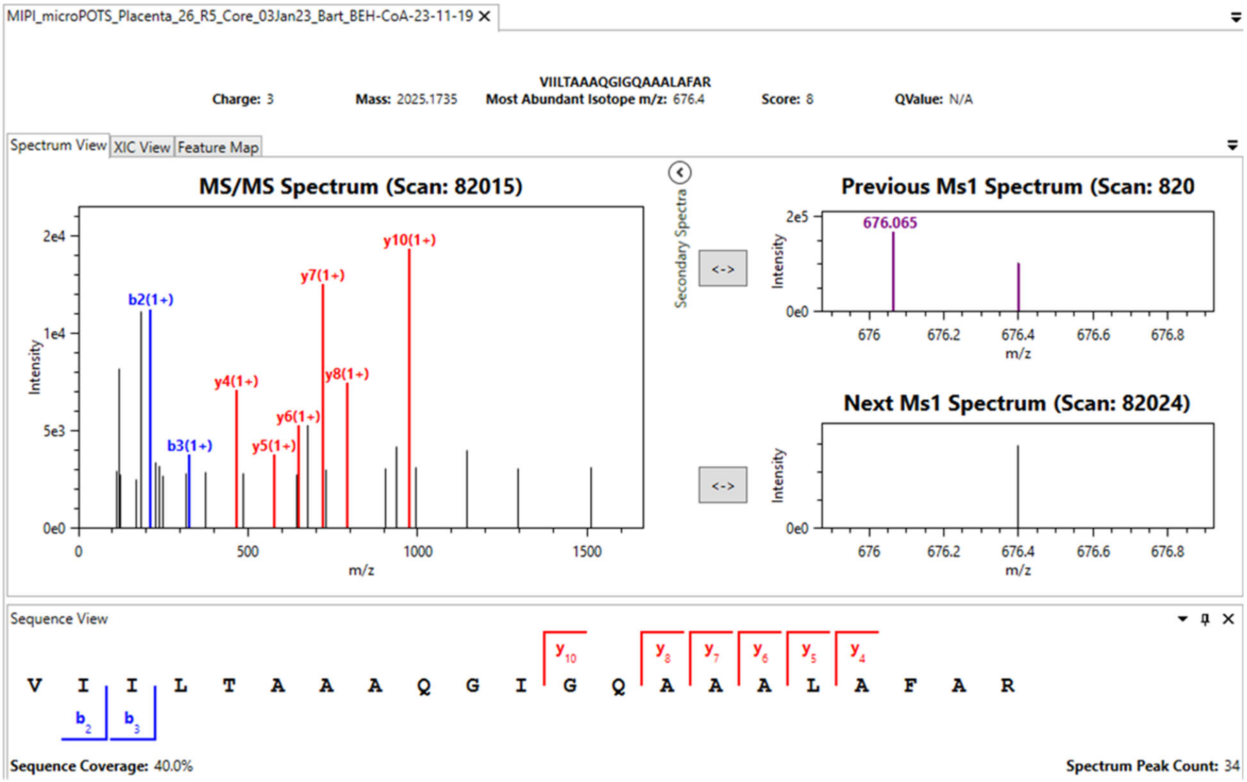

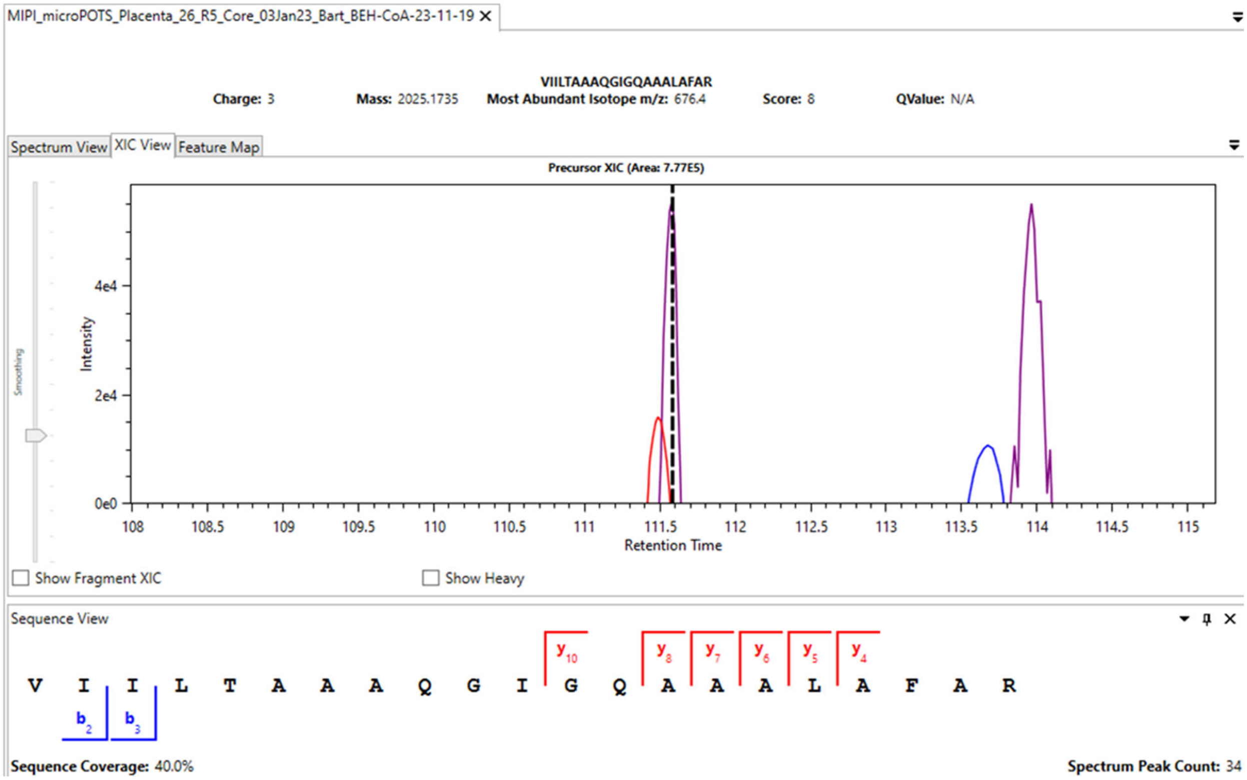

iii) Dataset: MIPI_microPOTS_Placenta_26_R2_Core_03Jan23_Bart_BEH-CoA-23-11-19; Scan Number for MS/MS: 83062

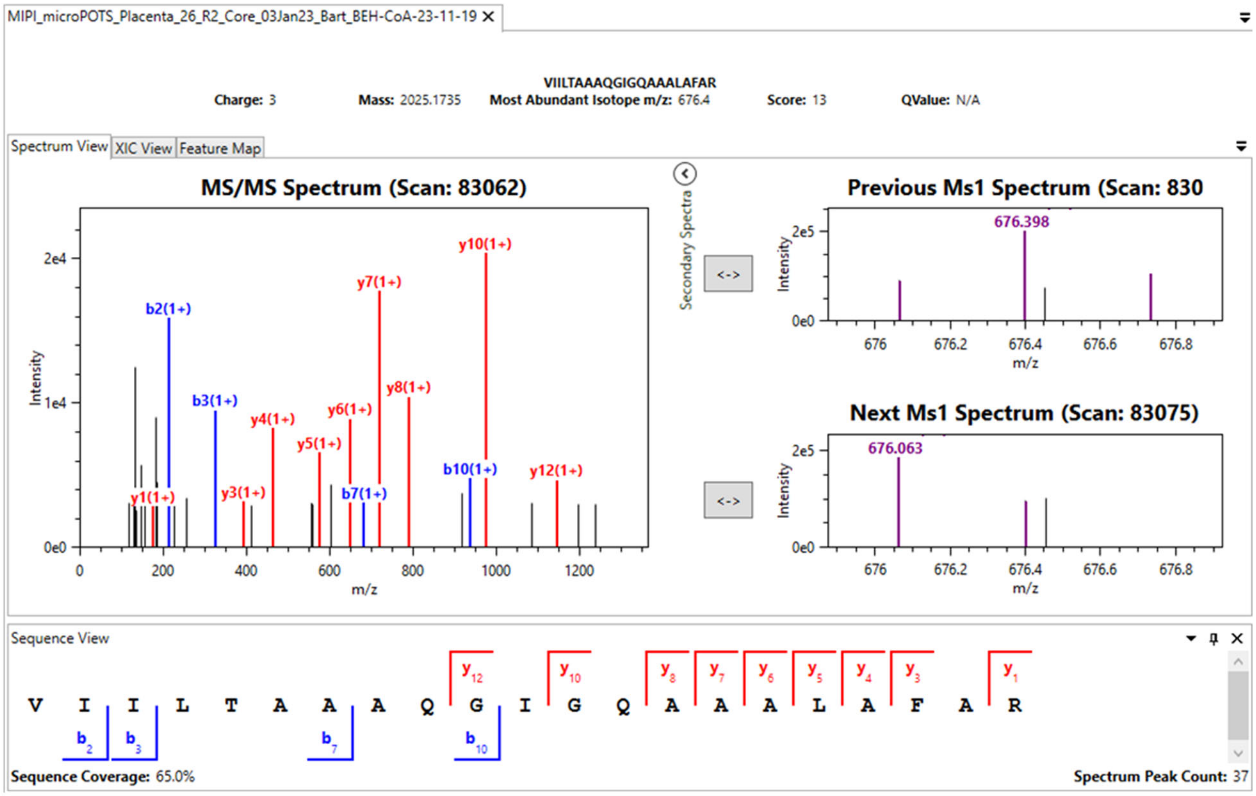

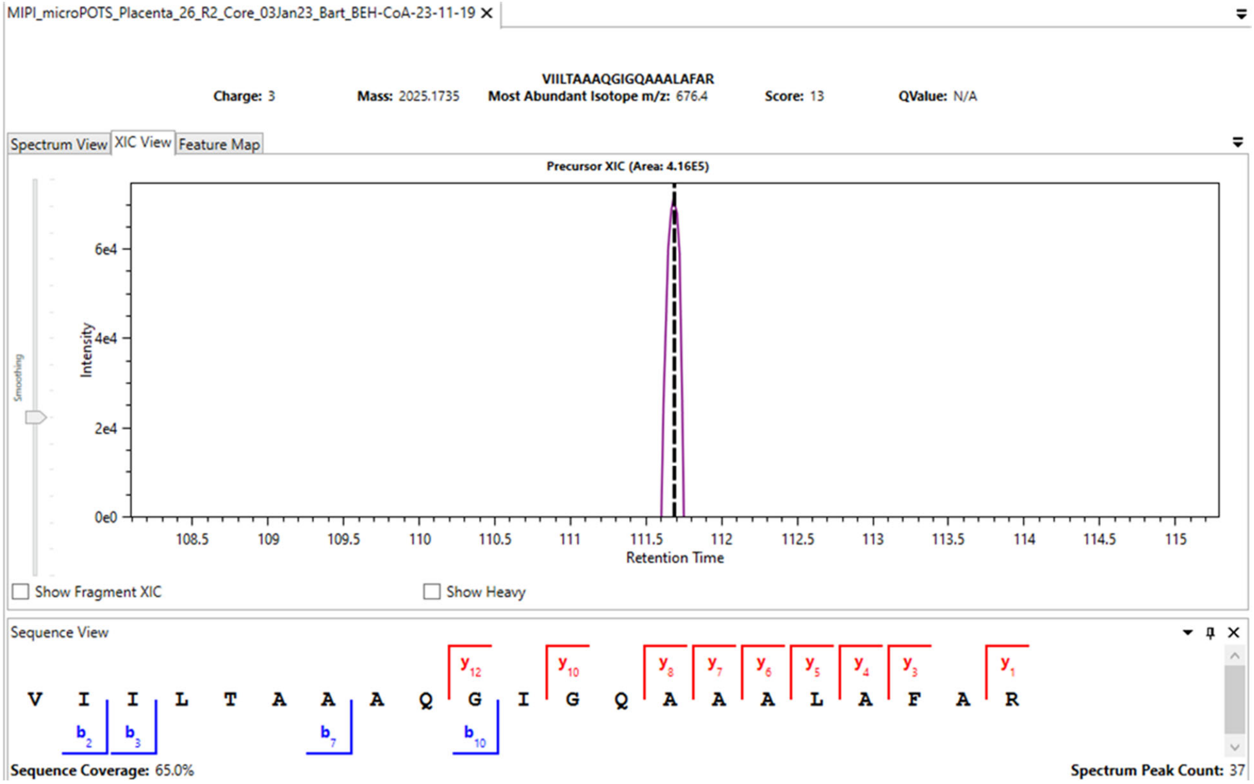

iv) Dataset: MIPI_microPOTS_Placenta_19_R5_Core_03Jan23_Bart_BEH-CoA-23-11-19; Scan Number for MS/MS: 84993

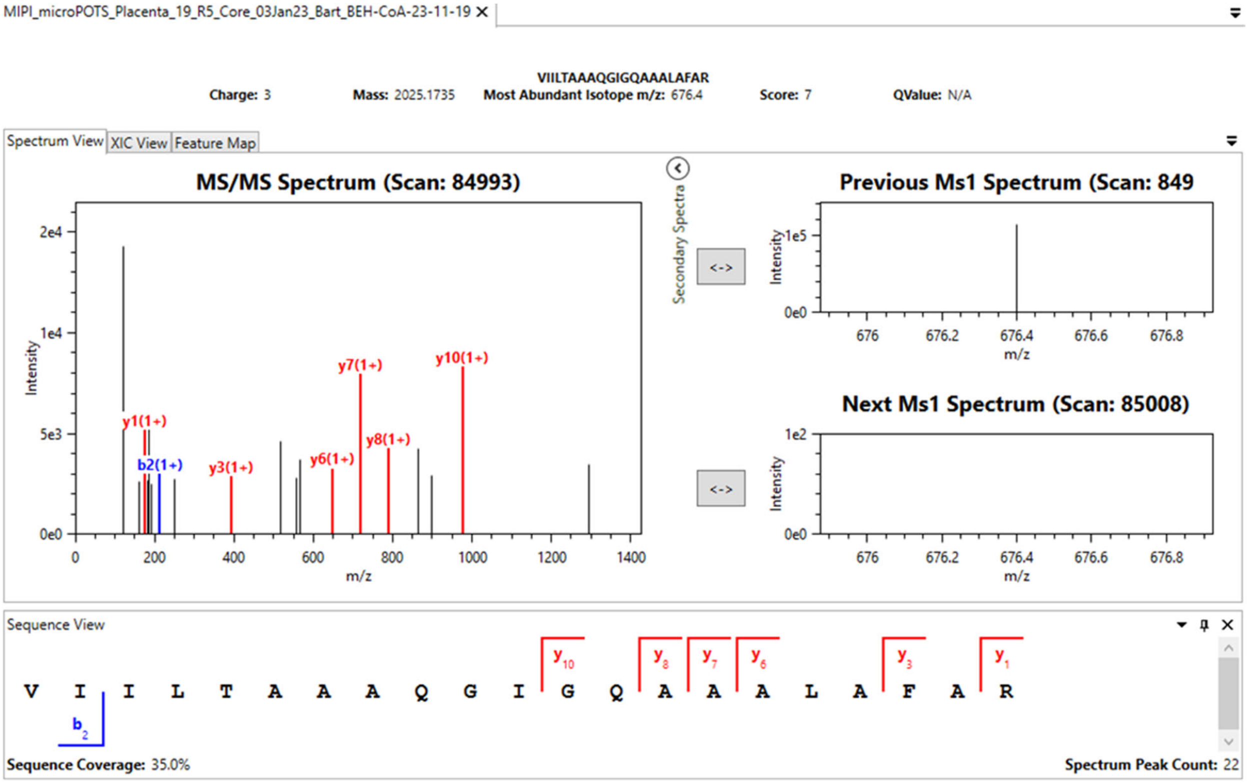

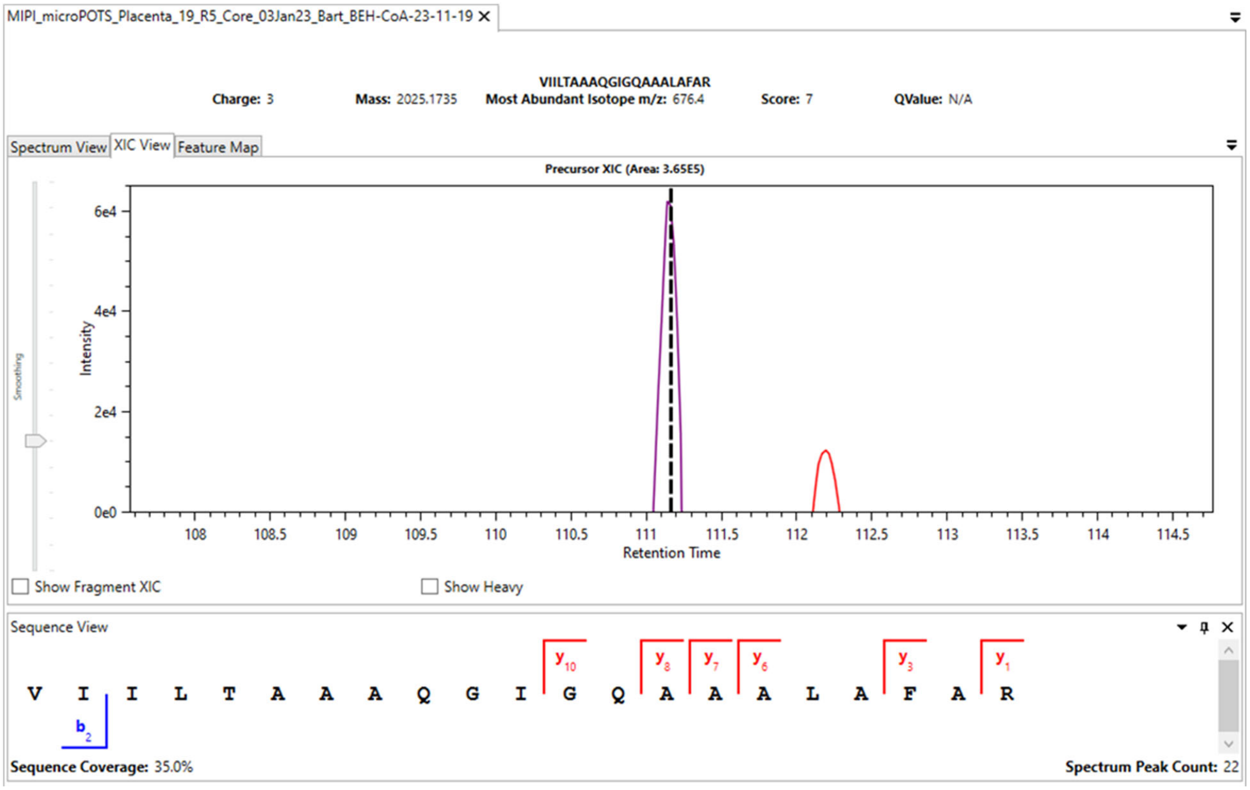

f) Protein: sp|P23786|CPT2_HUMAN; Peptide: K.SEYNDQLTR.A

i) Dataset: MIPI_microPOTS_Placenta_26_R3_STB_03Jan23_Bart_BEH-CoA-23-11-19; Scan Number for MS/MS: 12157

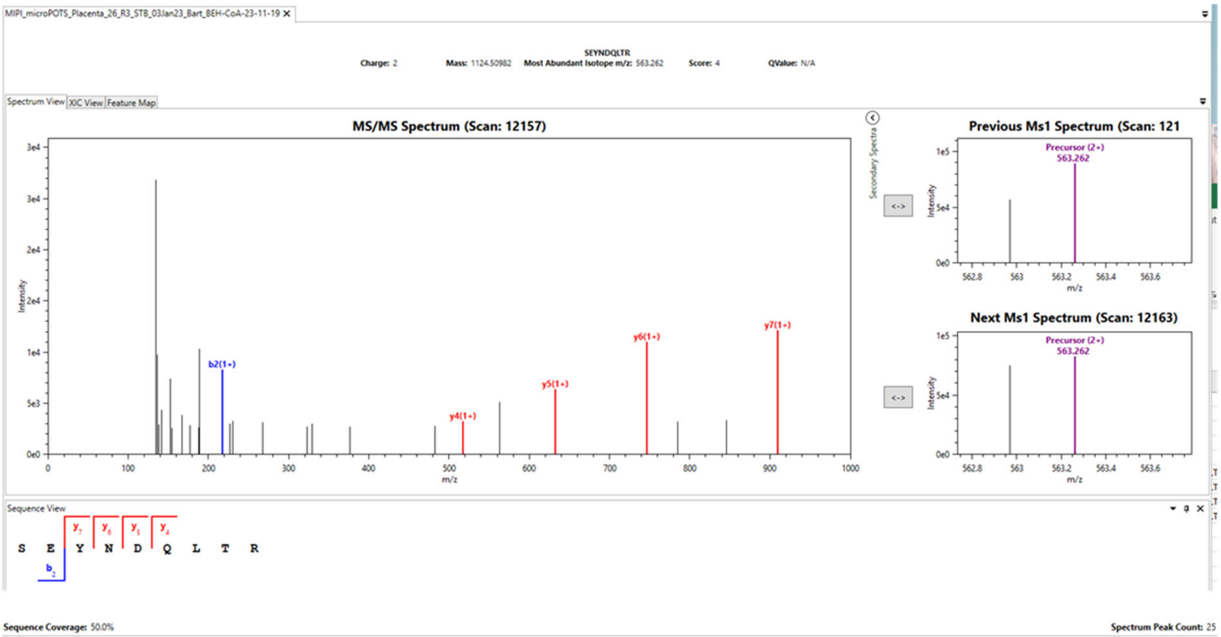

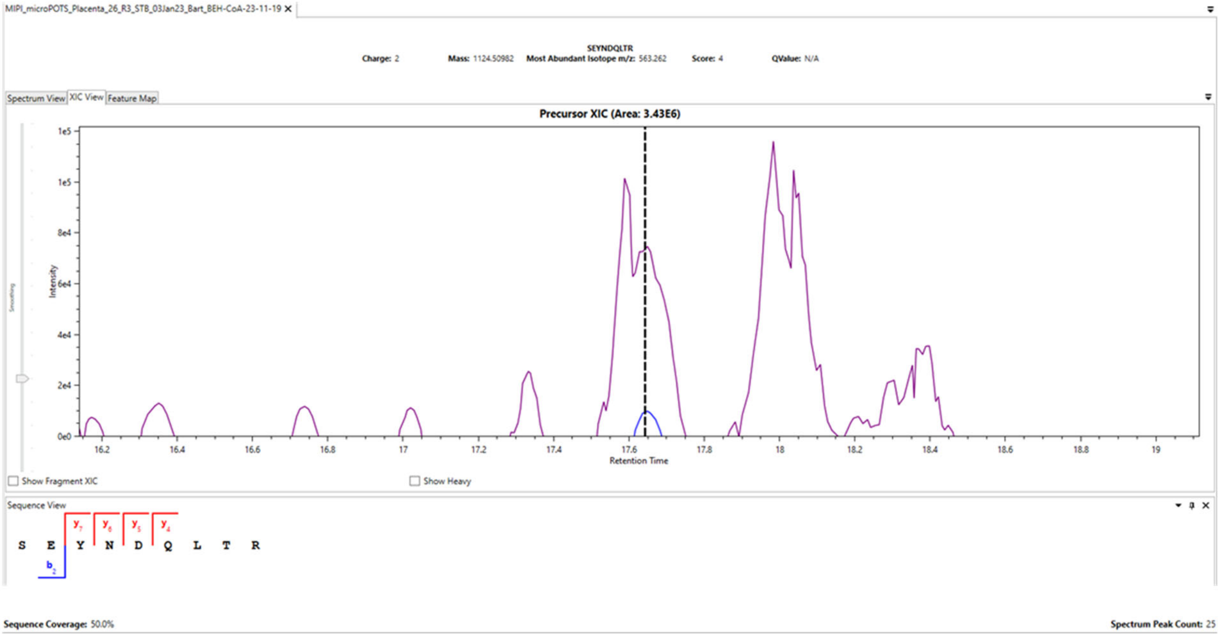

ii) Dataset: MIPI_microPOTS_Placenta_23_R1_STB_03Jan23_Bart_BEH-CoA-23-11-19; Scan Number for MS/MS: 11632

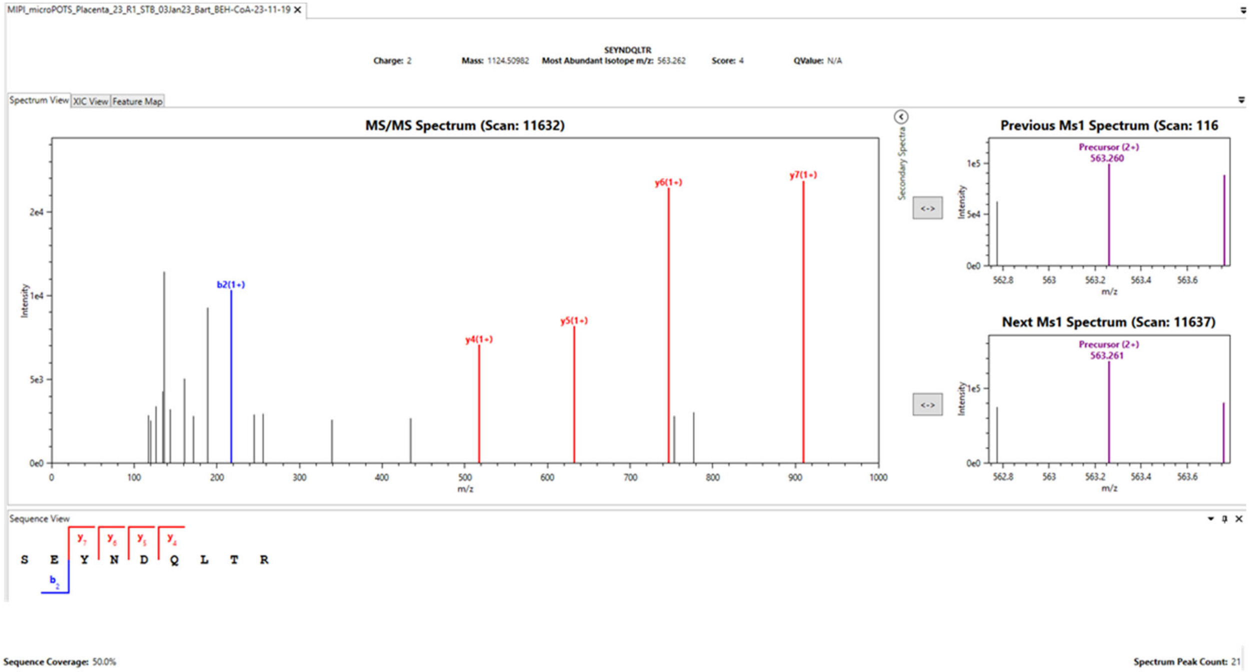

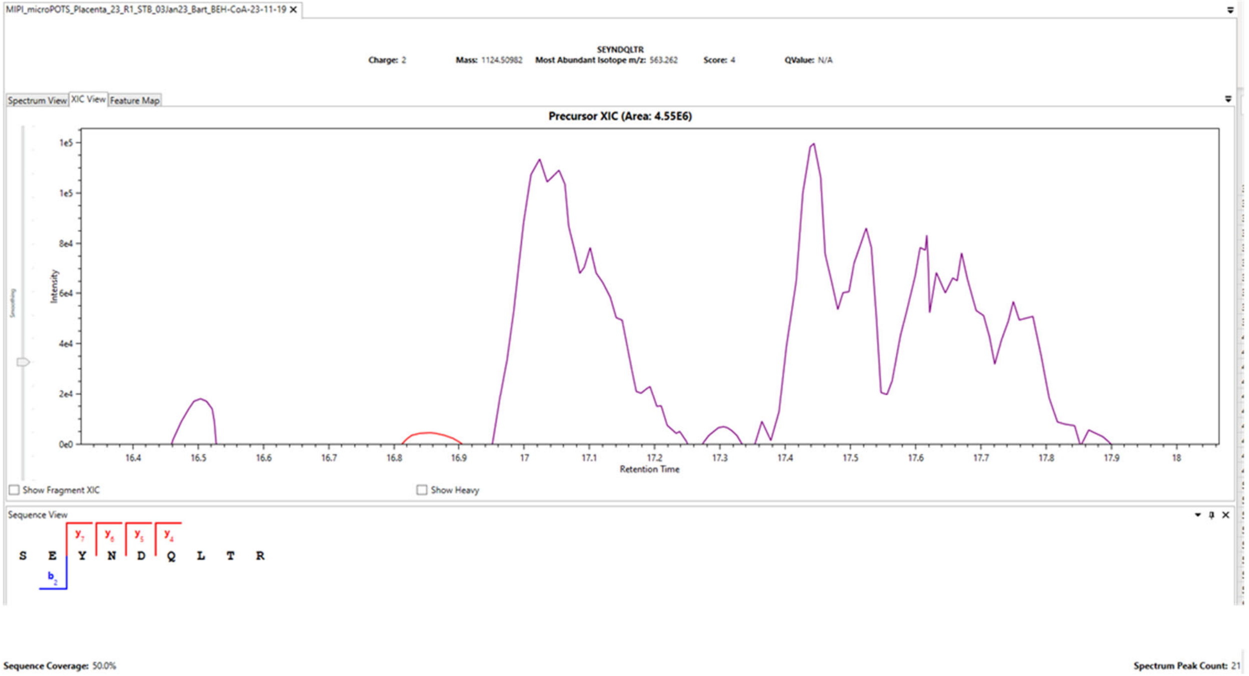

iii) Dataset: MIPI_microPOTS_Placenta_19_R1_STB_03Jan23_Bart_BEH-CoA-23-11-19; Scan Number for MS/MS: 11733

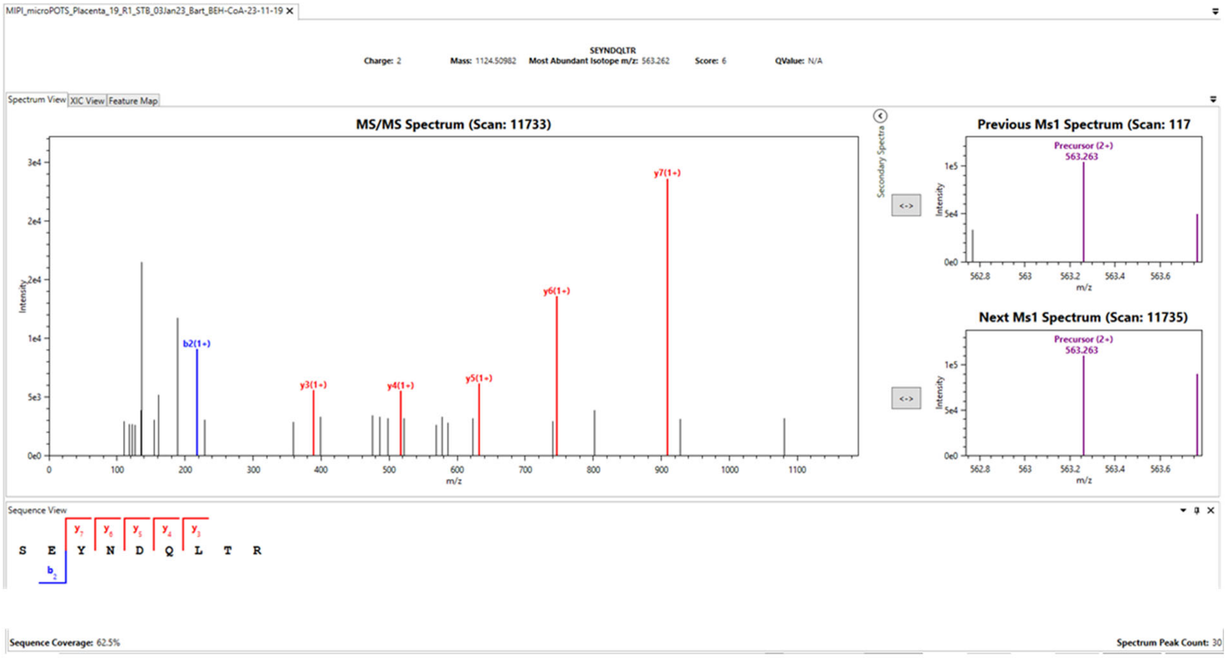

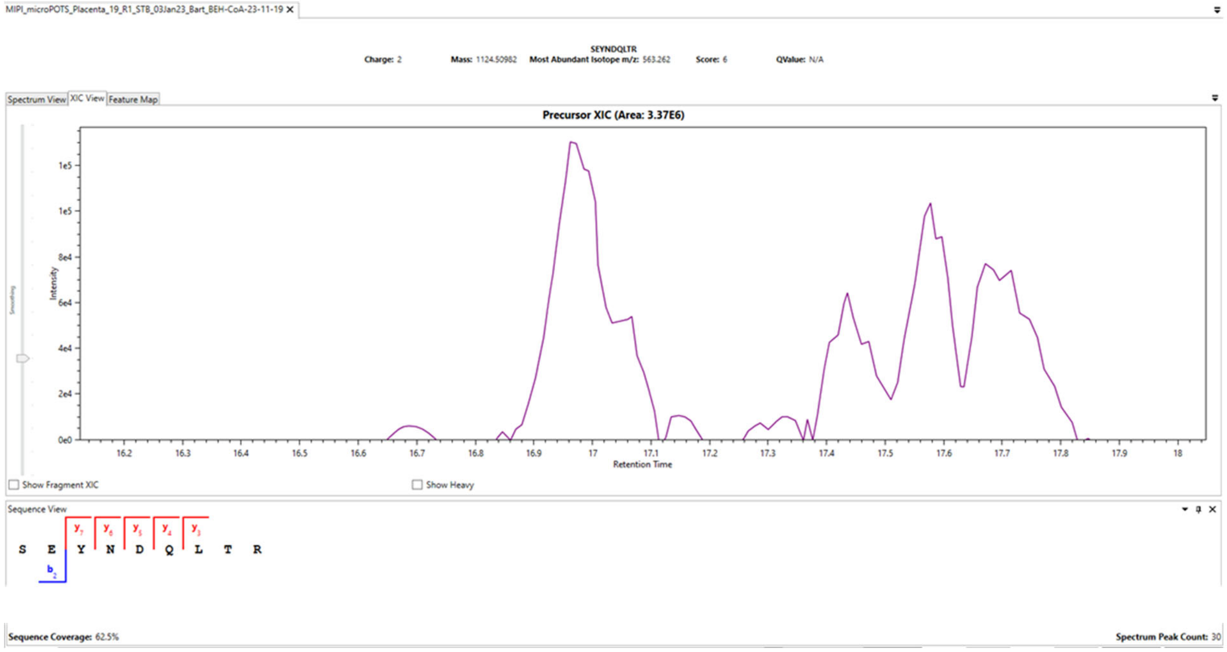

g) **Protein: sp|P26572|MGAT1_HUMAN; Peptide: R.LAQDAEVELER.Q**

i) Dataset: MIPI_microPOTS_Placenta_26_R5_STB_03Jan23_Bart_BEH-CoA-23-11-19; Scan Number for MS/MS: 27226

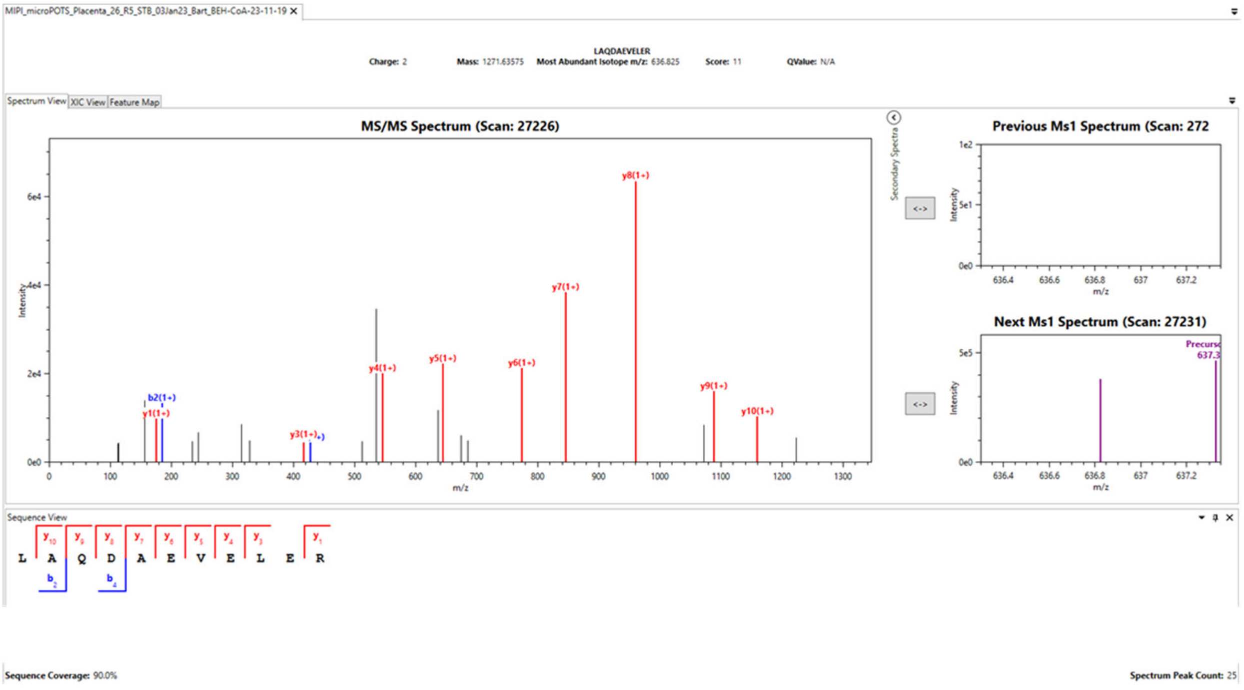

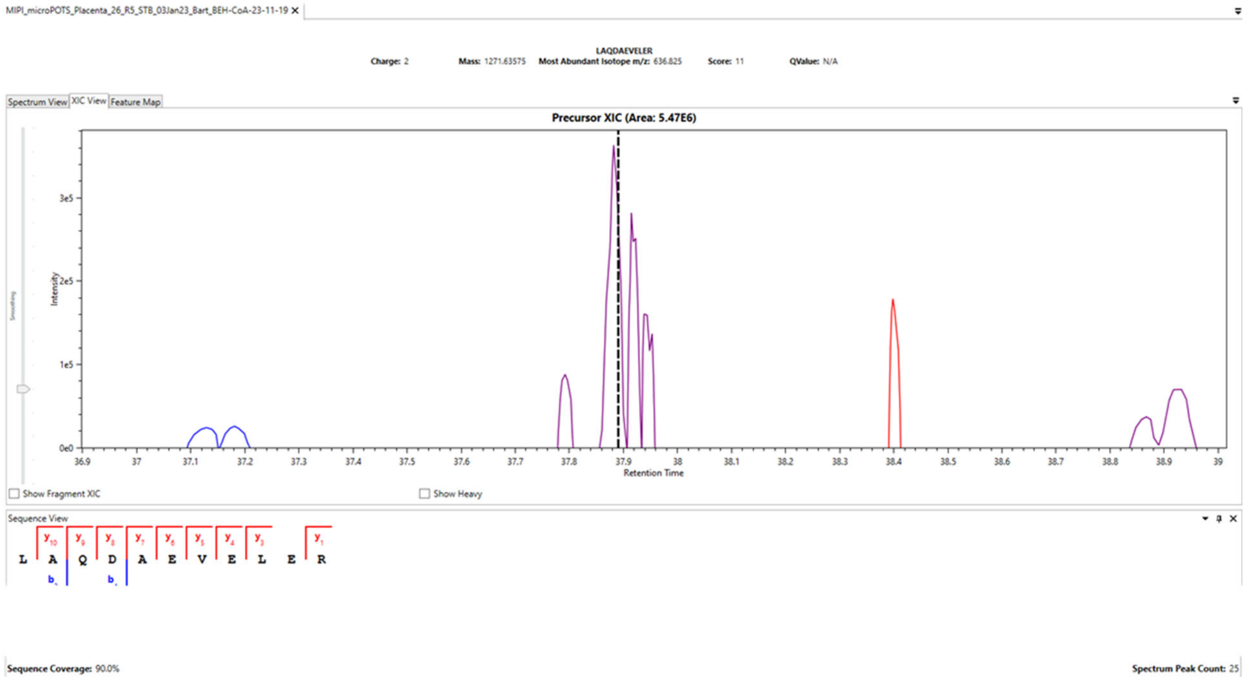

ii) Dataset: MIPI_microPOTS_Placenta_26_R1_STB_03Jan23_Bart_BEH-CoA-23-11-19; Scan Number for MS/MS: 25313

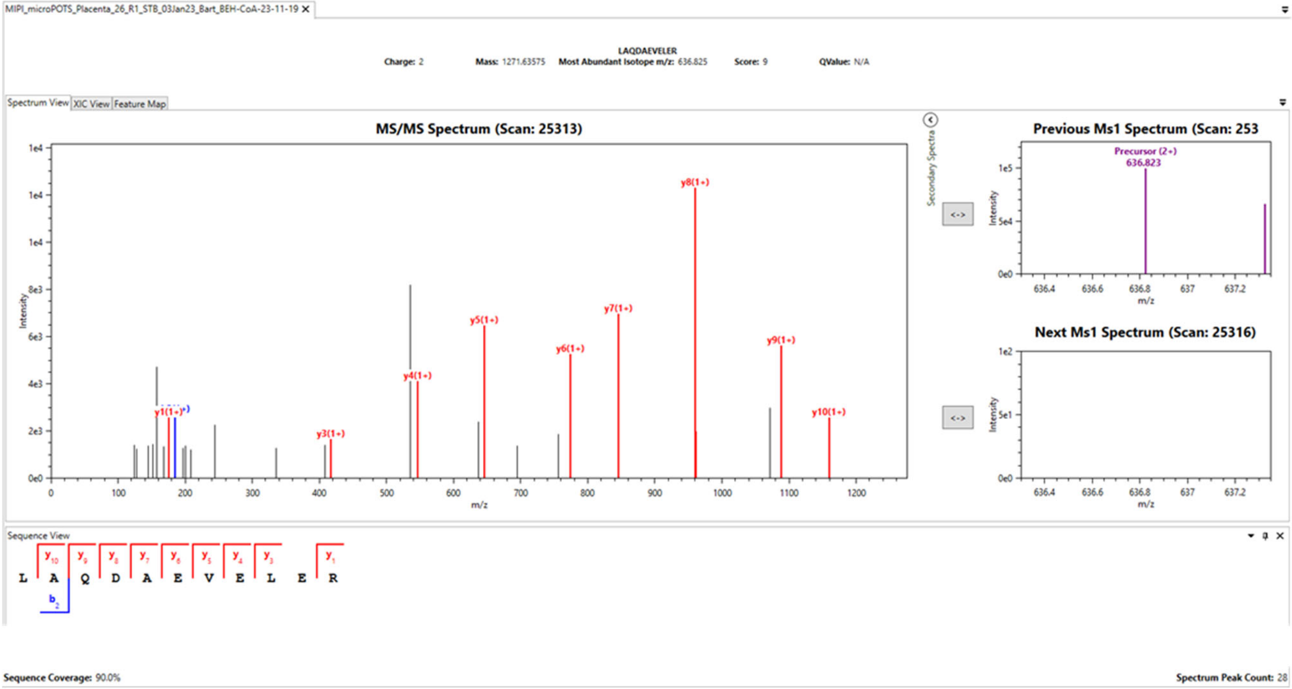

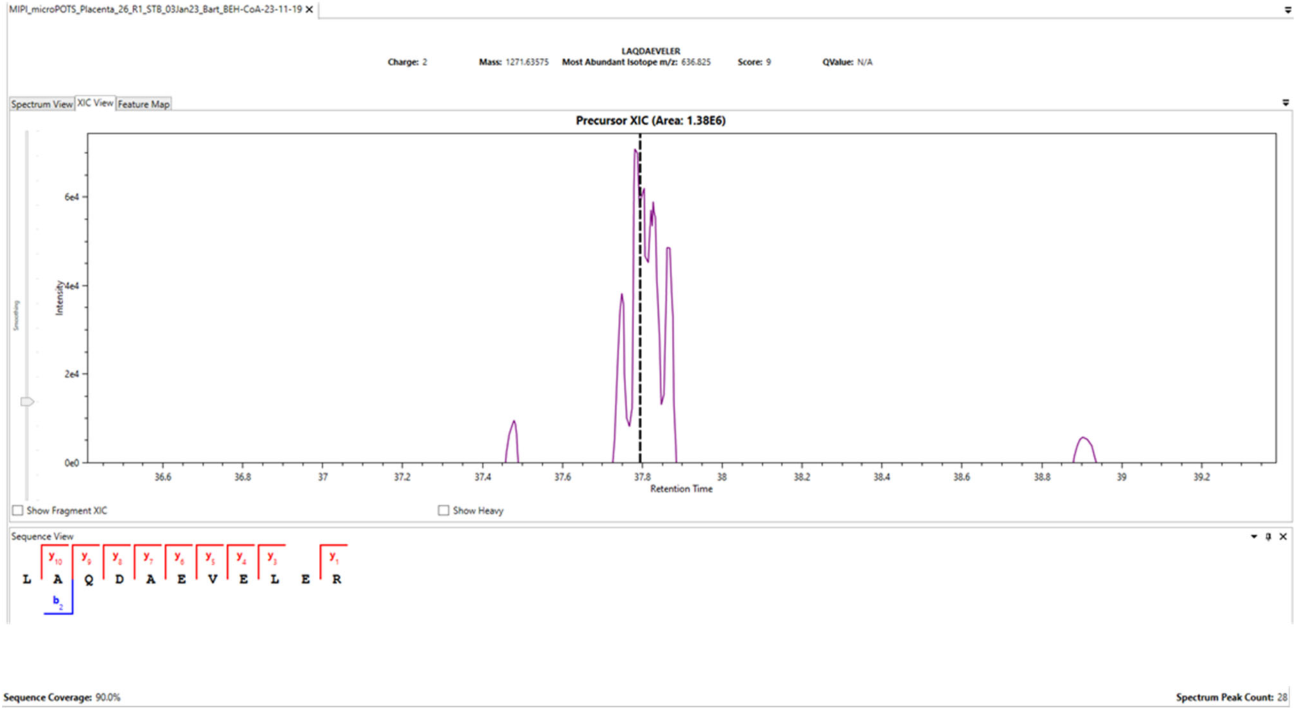

iii) Dataset: MIPI_microPOTS_Placenta_26_R2_STB_03Jan23_Bart_BEH-CoA-23-11-19; Scan Number for MS/MS: 26851

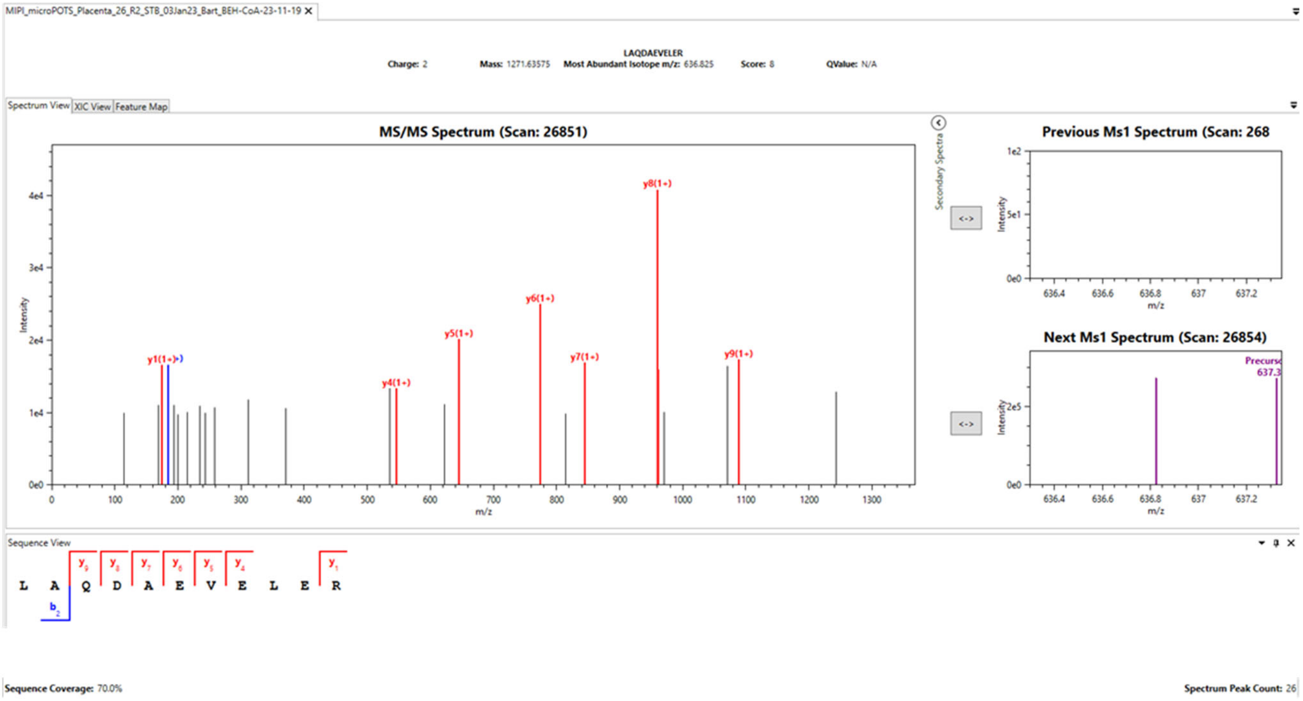

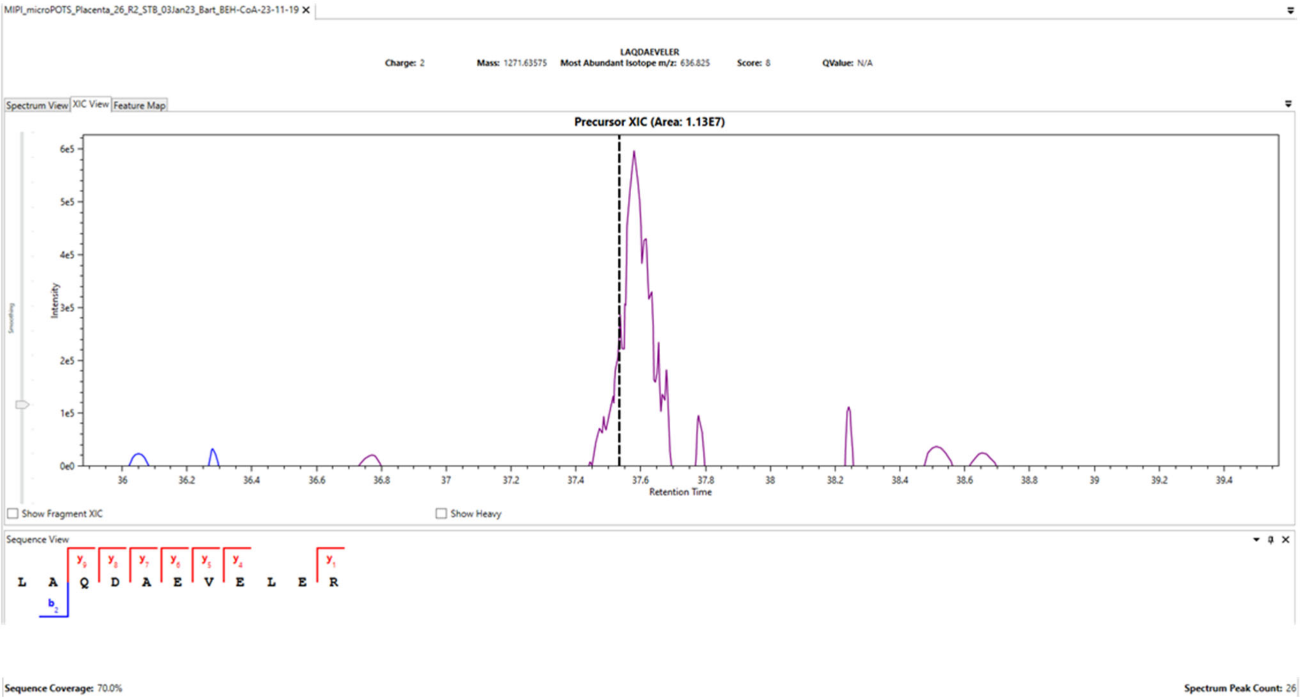

iv) Dataset: MIPI_microPOTS_Placenta_23_R5_STB_03Jan23_Bart_BEH-CoA-23-11-19; Scan Number for MS/MS: 26433

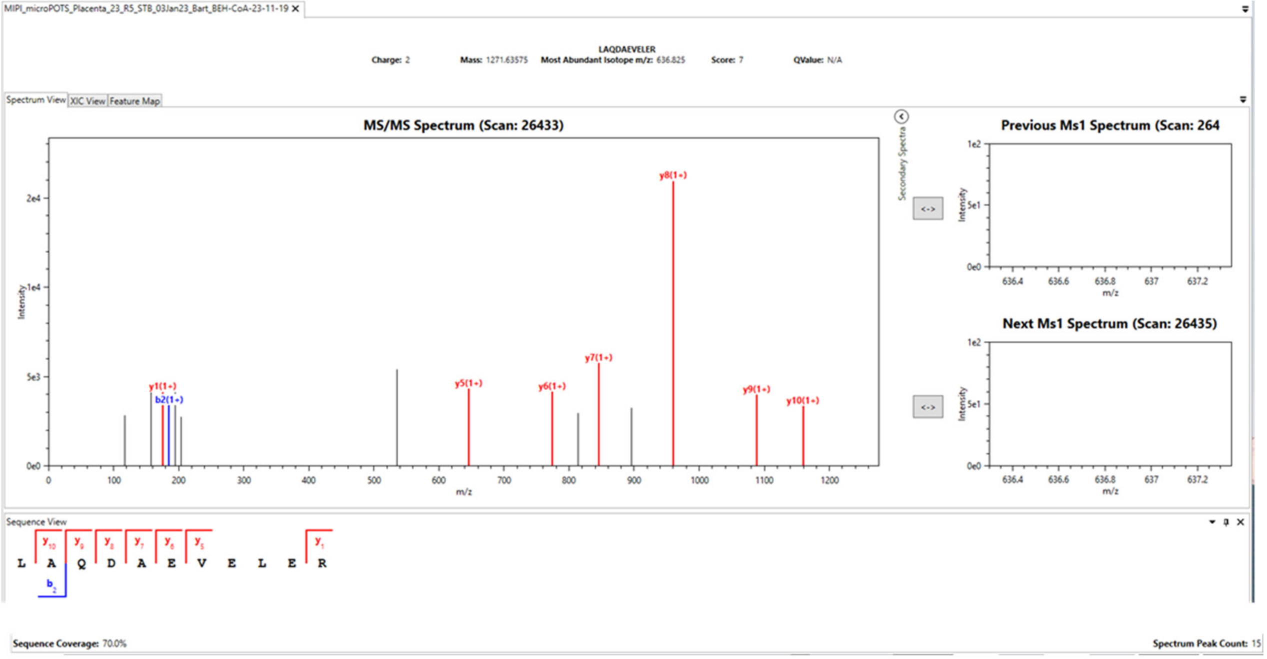

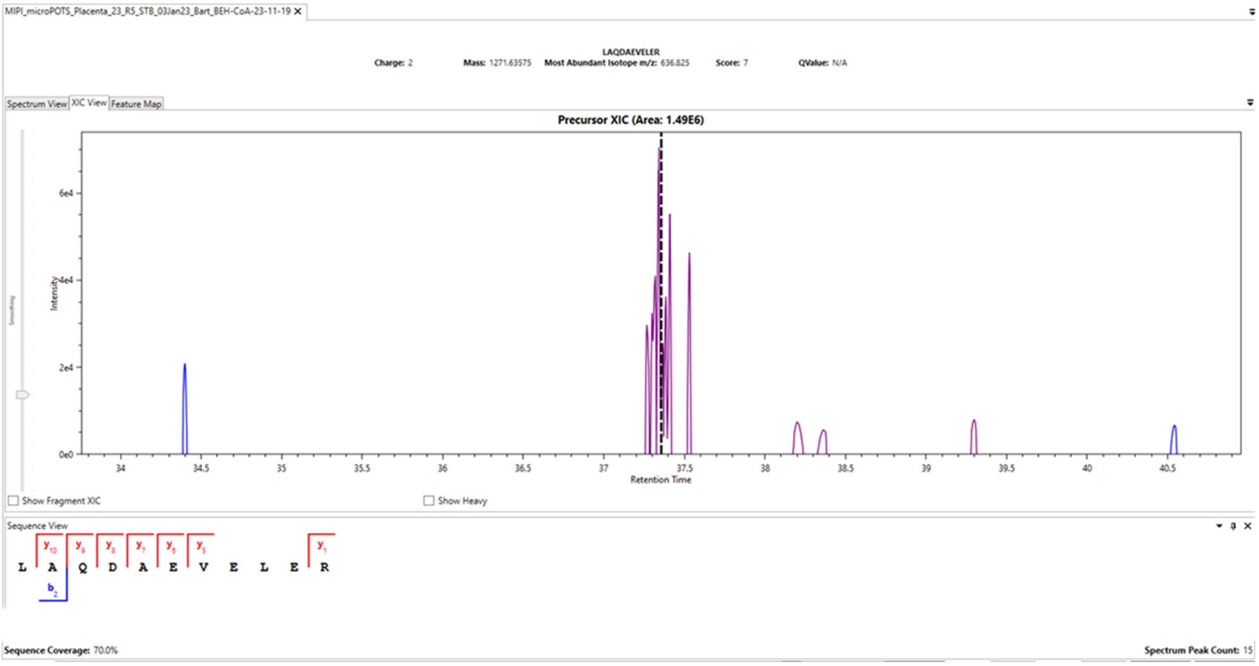

h) Protein: sp|P13073|COX41_HUMAN; Peptide: K.ESFAEM*NR.G

i) Dataset: MIPI_microPOTS_Placenta_26_R5_STB_03Jan23_Bart_BEH-CoA-23-11-19; Scan Number for MS/MS: 7625

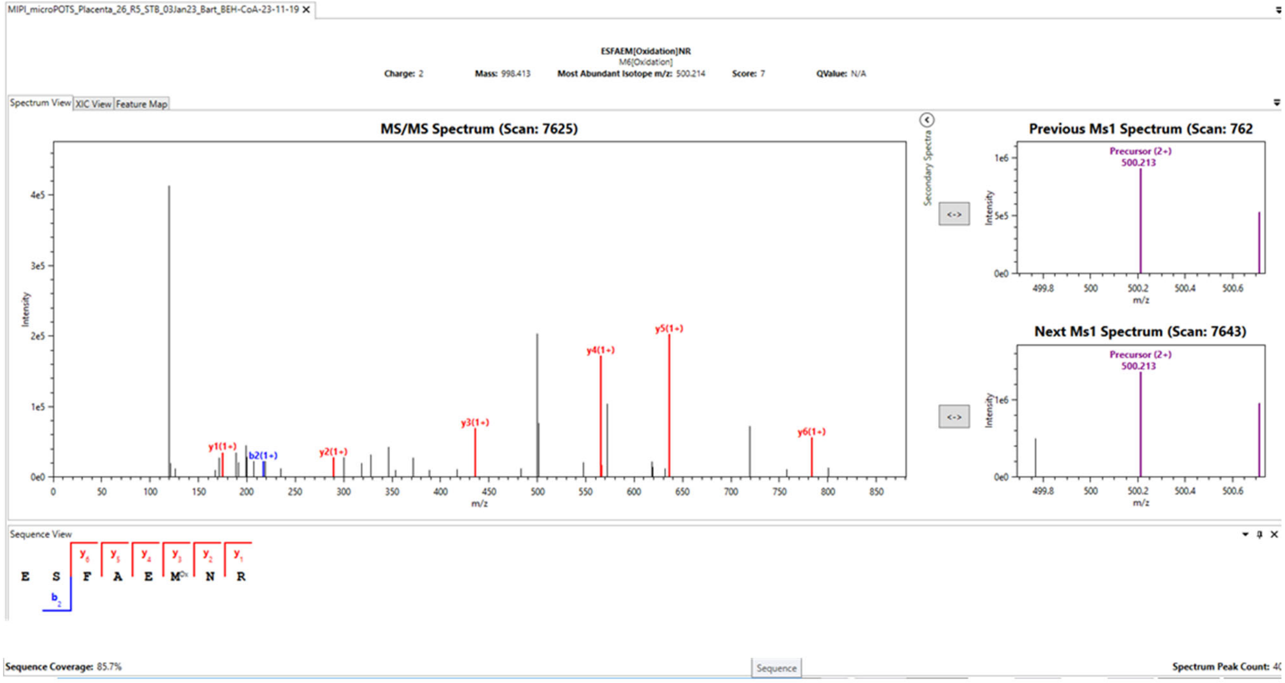

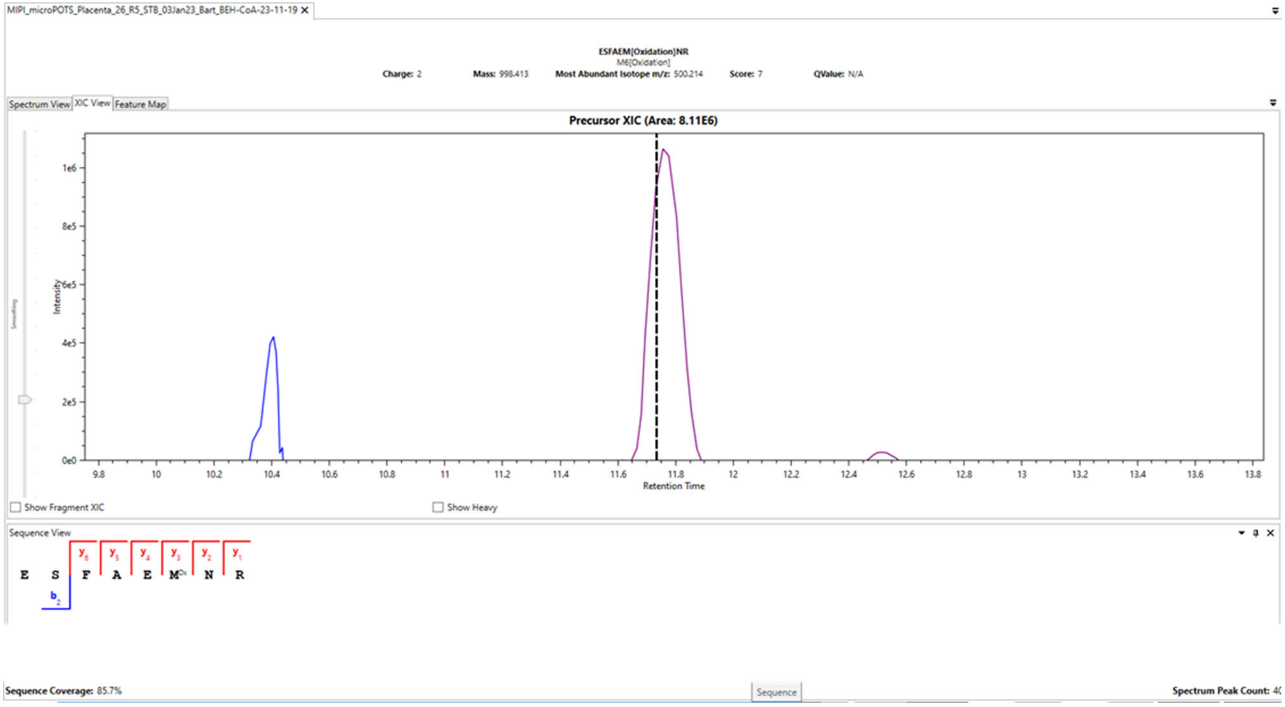

ii) Dataset: MIPI_microPOTS_Placenta_26_R4_STB_03Jan23_Bart_BEH-CoA-23-11-19; Scan Number for MS/MS: 7572

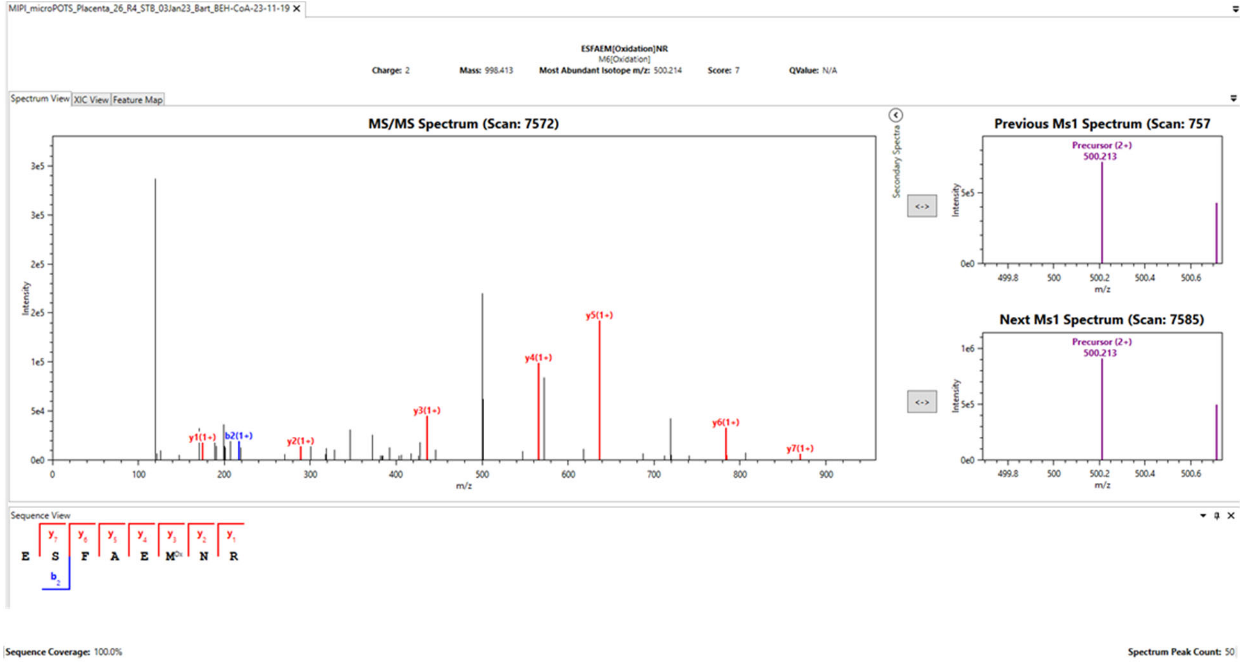

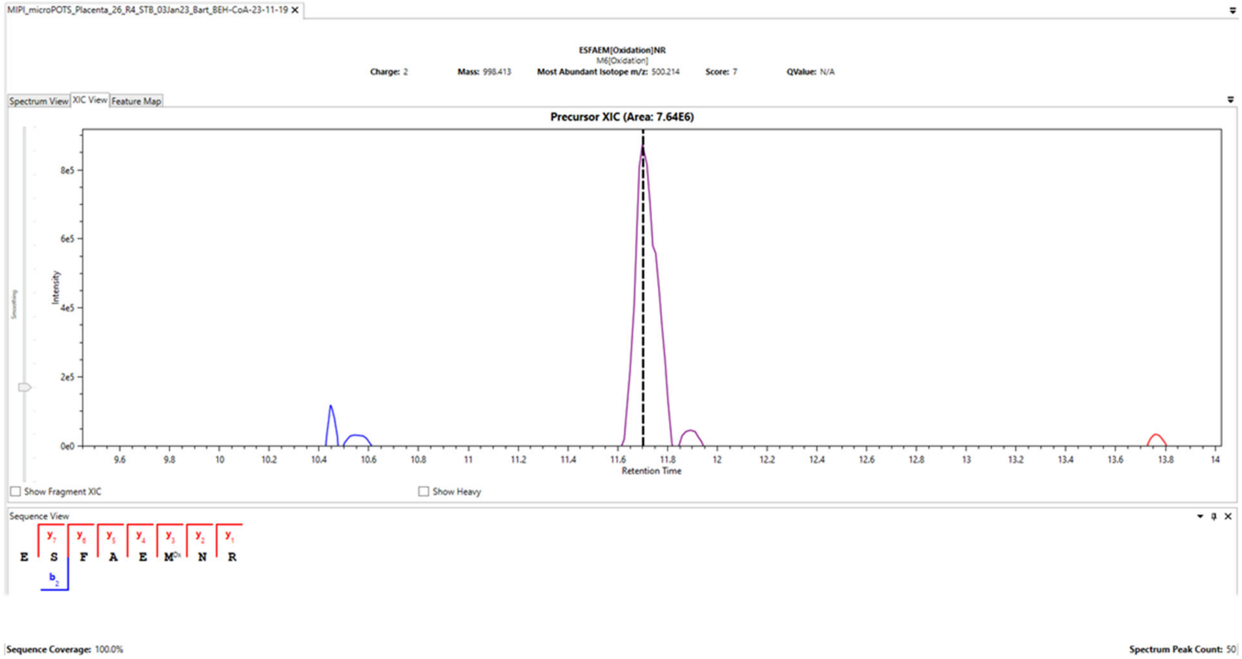

iii) Dataset: MIPI_microPOTS_Placenta_26_R1_STB_03Jan23_Bart_BEH-CoA-23-11-19; Scan Number for MS/MS: 7376

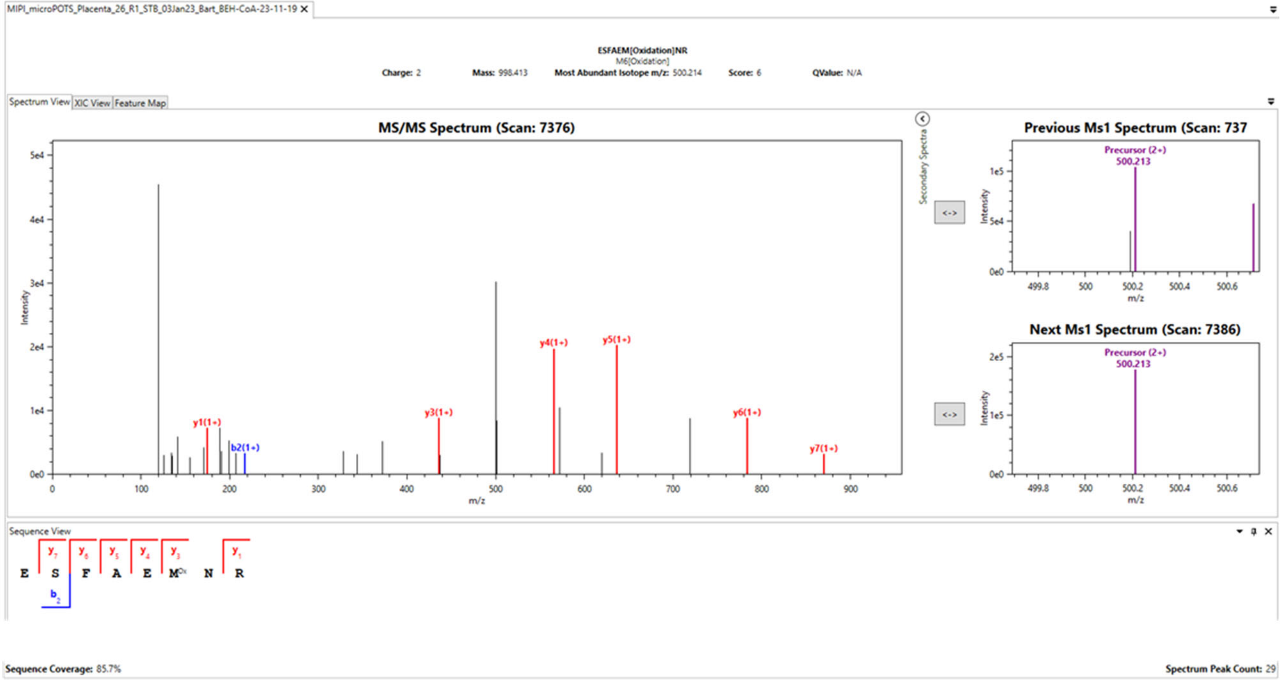

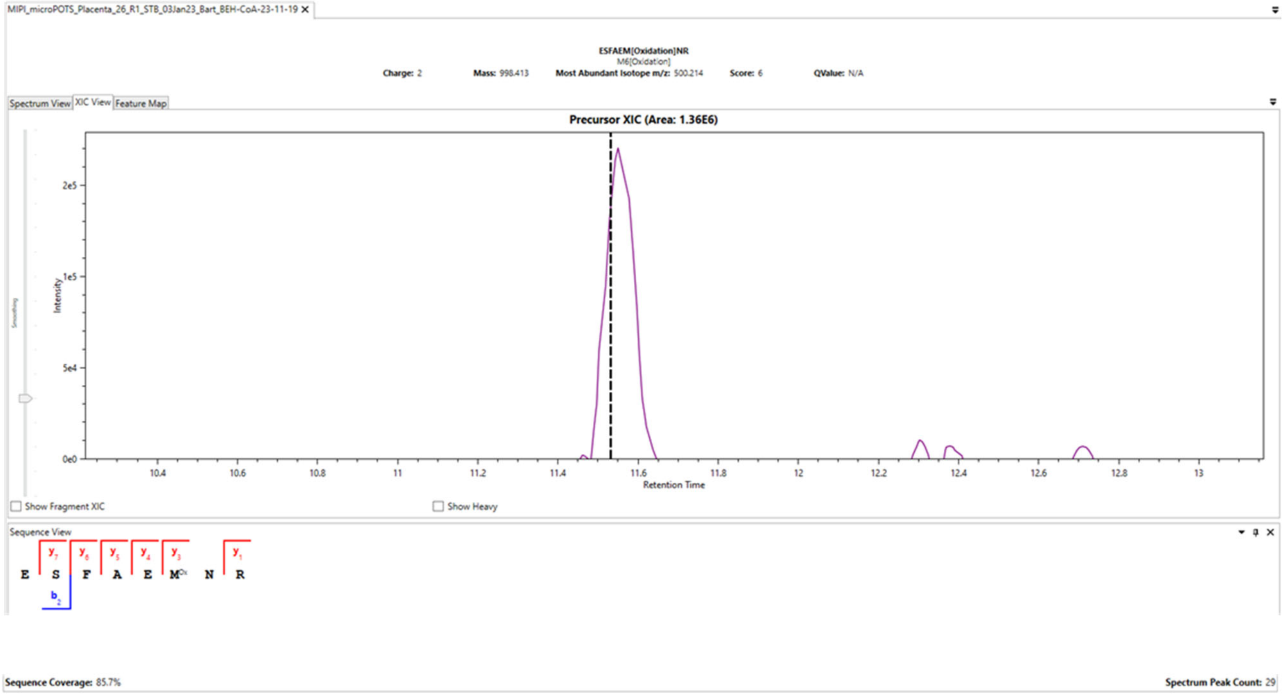

iv) Dataset: MIPI_microPOTS_Placenta_26_R2_STB_03Jan23_Bart_BEH-CoA-23-11-19; Scan Number for MS/MS: 7432

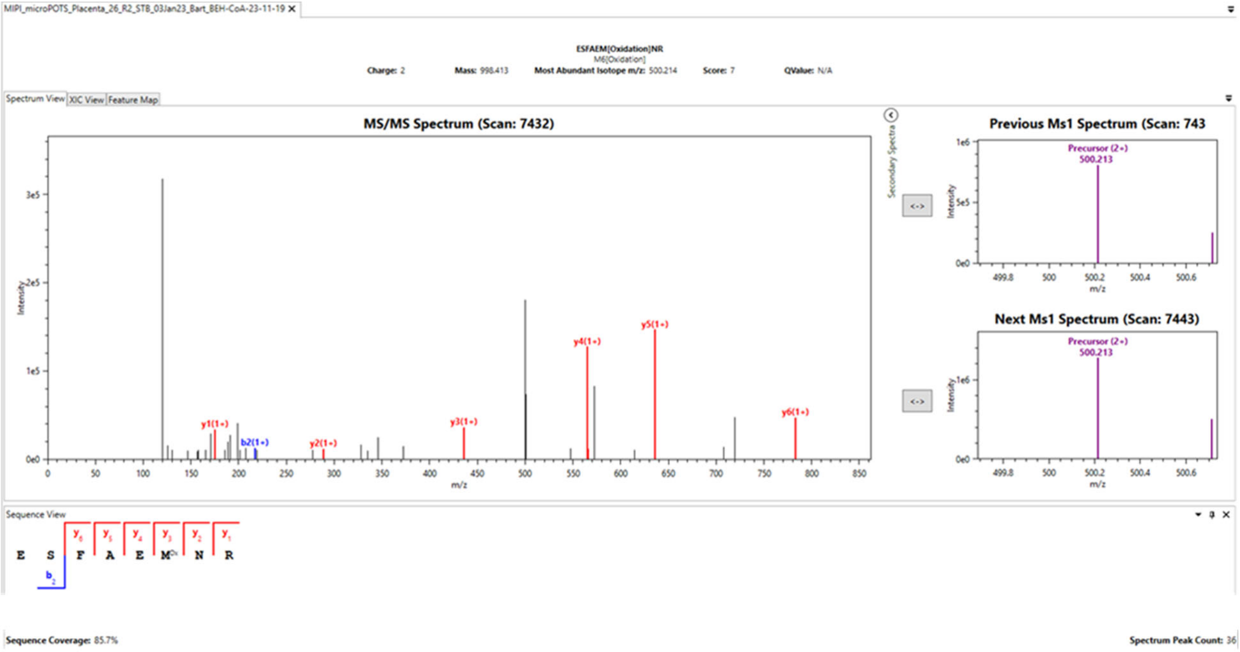

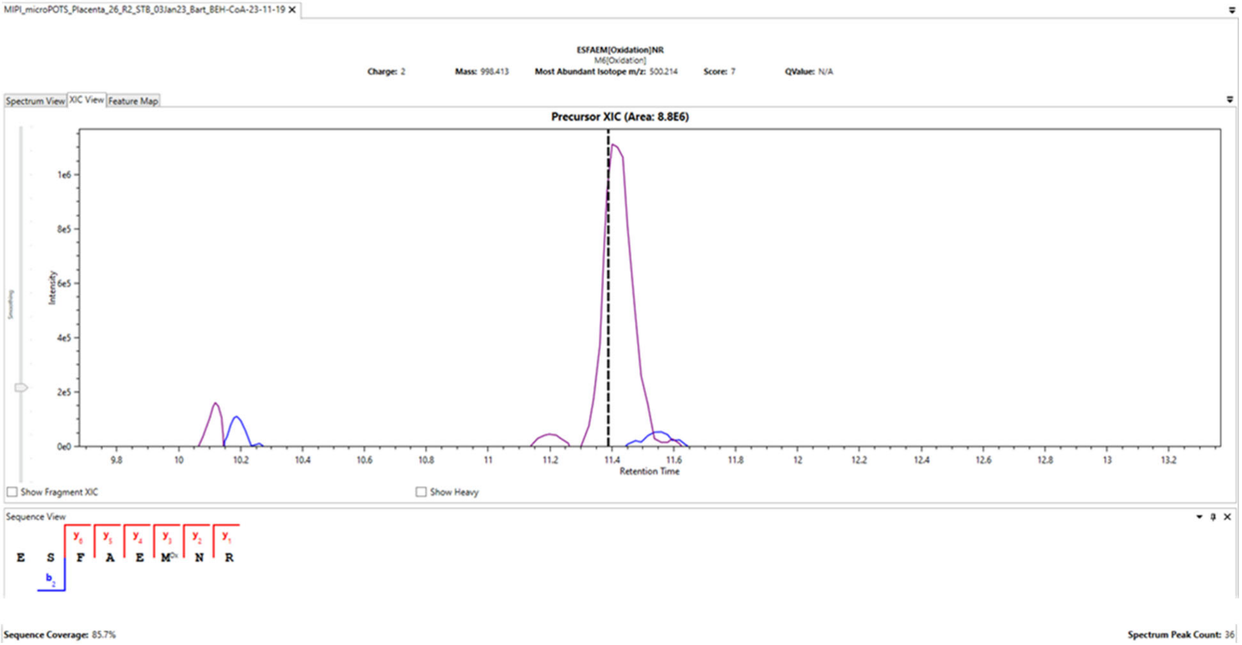

v) Dataset: MIPI_microPOTS_Placenta_26_R2_Core_03Jan23_Bart_BEH-CoA-23-11-19; Scan Number for MS/MS: 7583

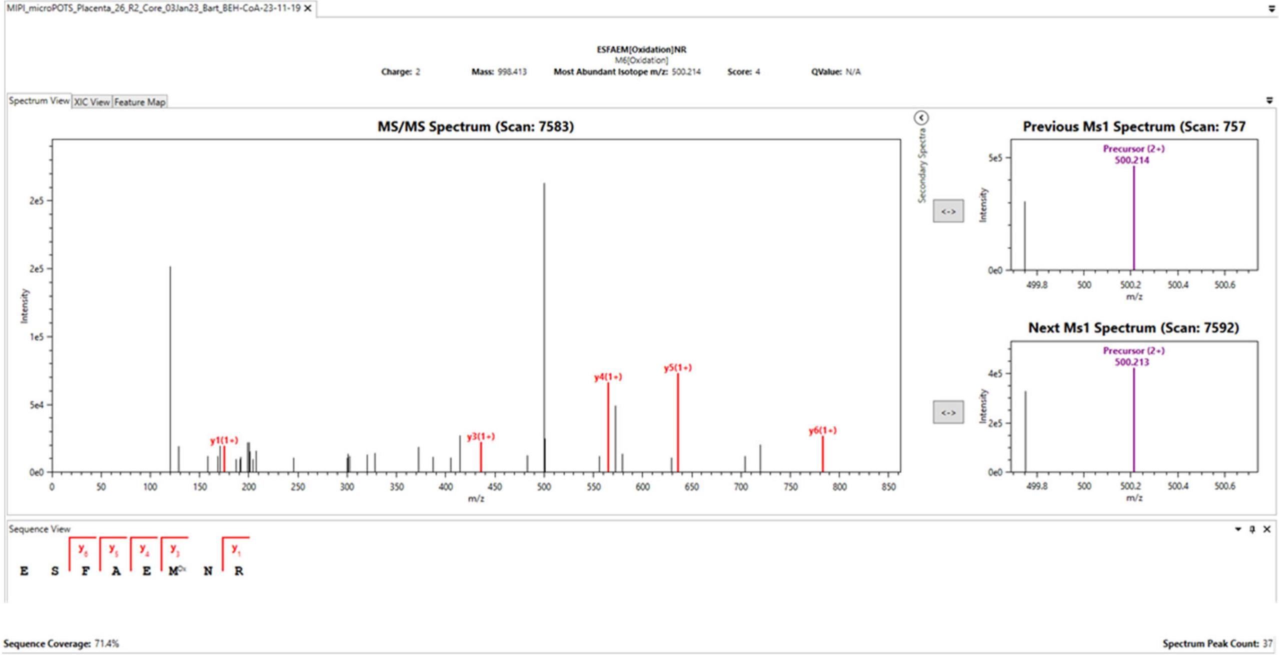

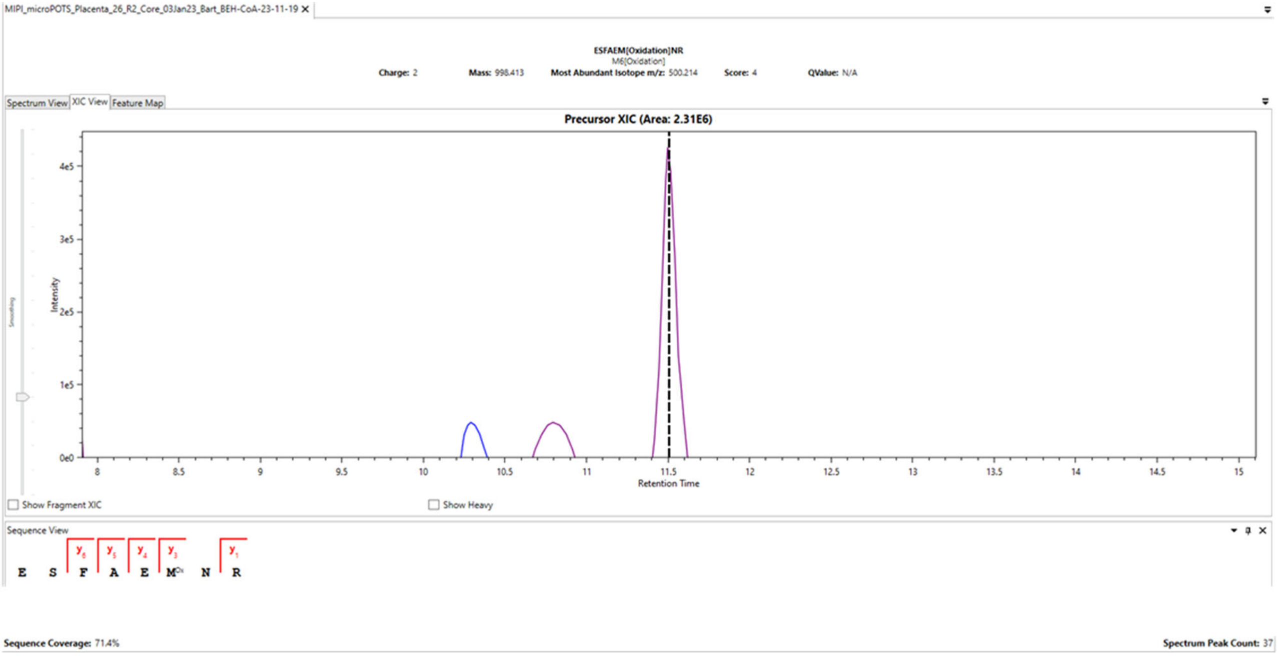

vi) Dataset: MIPI_microPOTS_Placenta_26_R3_STB_03Jan23_Bart_BEH-CoA-23-11-19; Scan Number for MS/MS: 7472

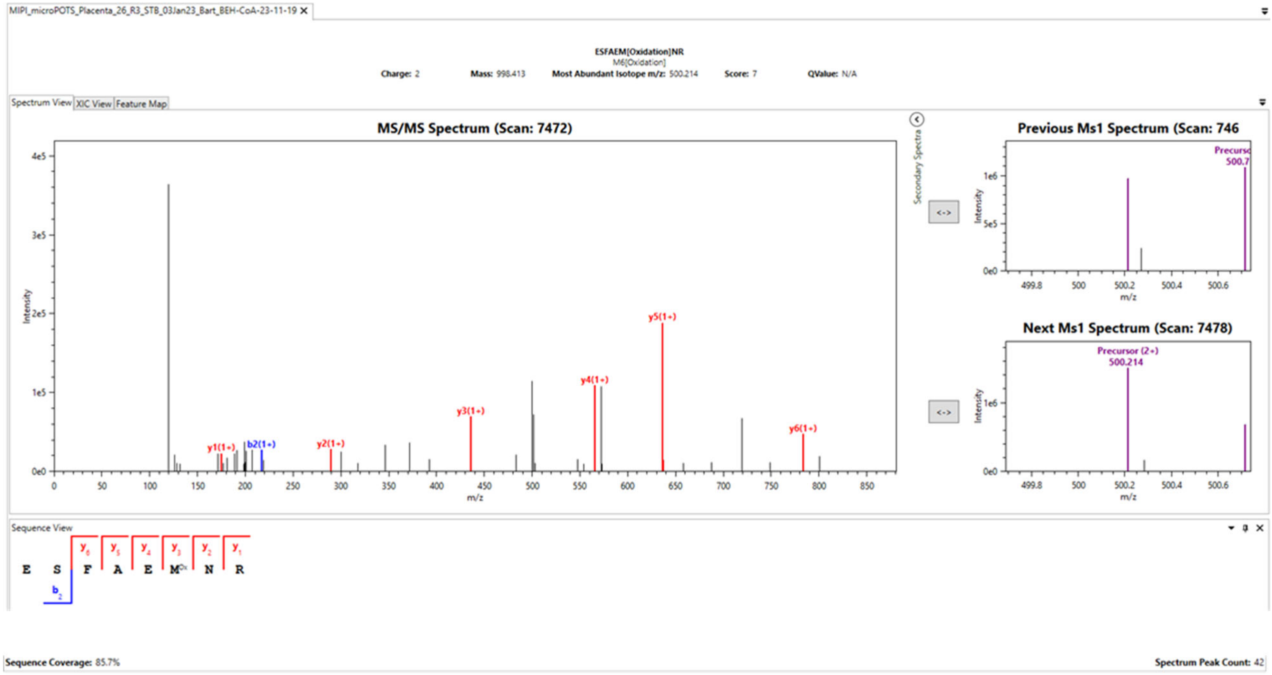

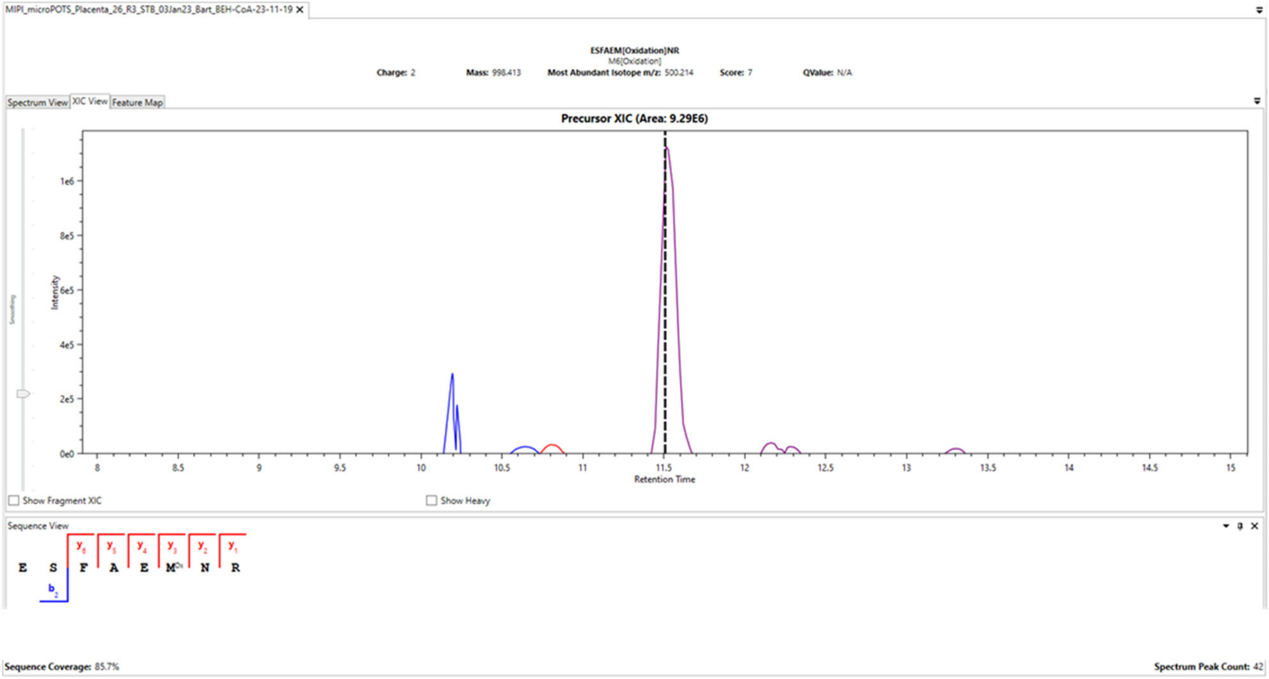

vii) Dataset: MIPI_microPOTS_Placenta_23_R4_STB_03Jan23_Bart_BEH-CoA-23-11-19; Scan Number for MS/MS: 7486

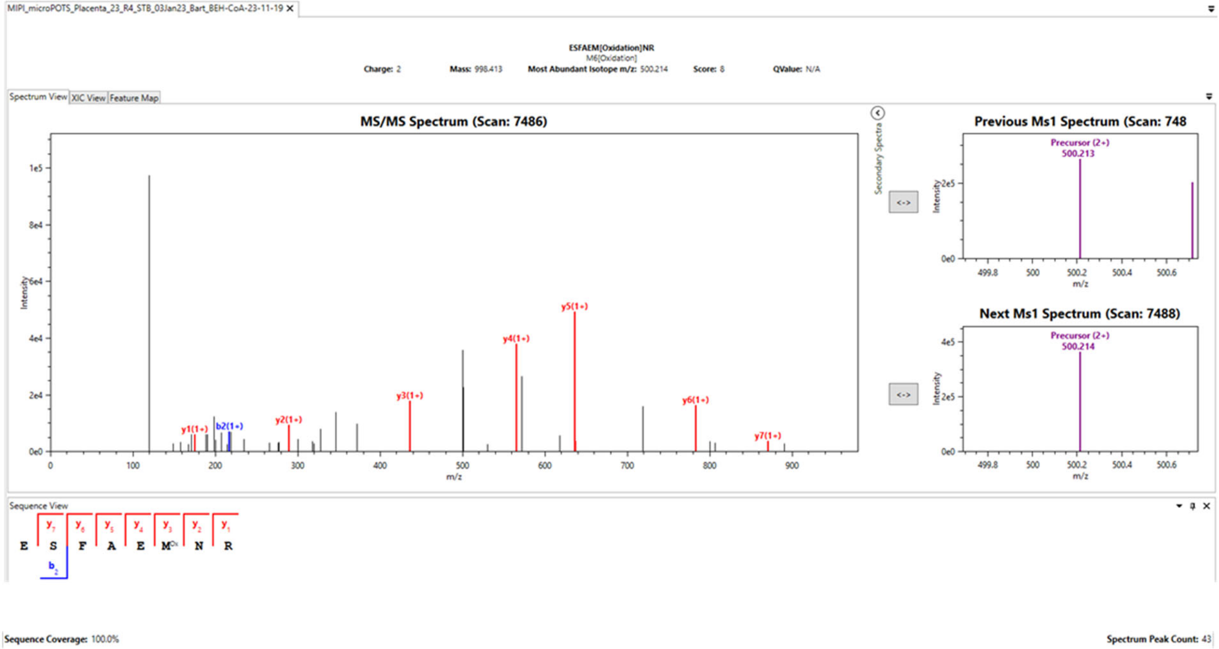

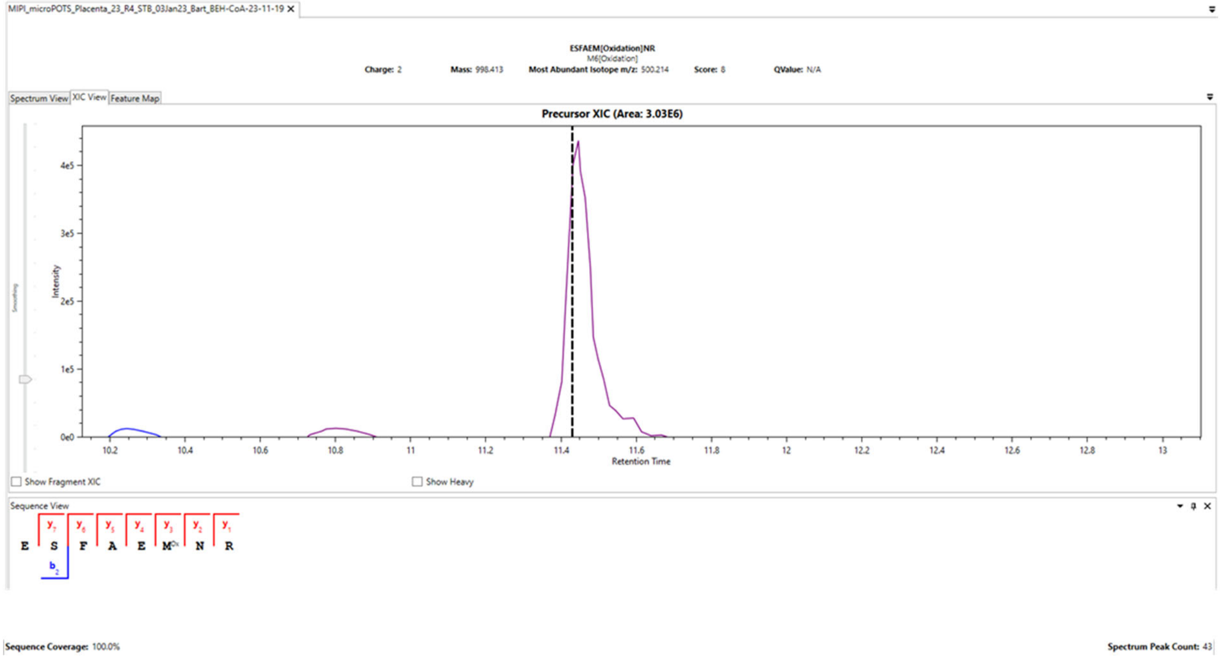

viii) Dataset: MIPI_microPOTS_Placenta_23_R2_STB_03Jan23_Bart_BEH-CoA-23-11-19; Scan Number for MS/MS: 7309

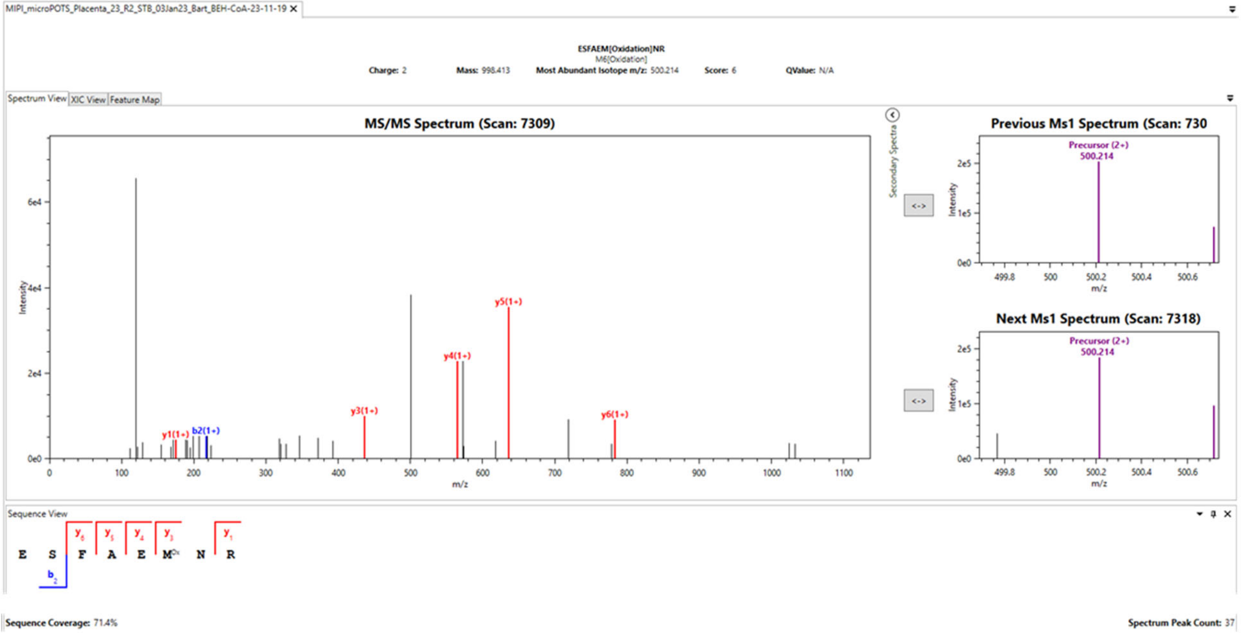

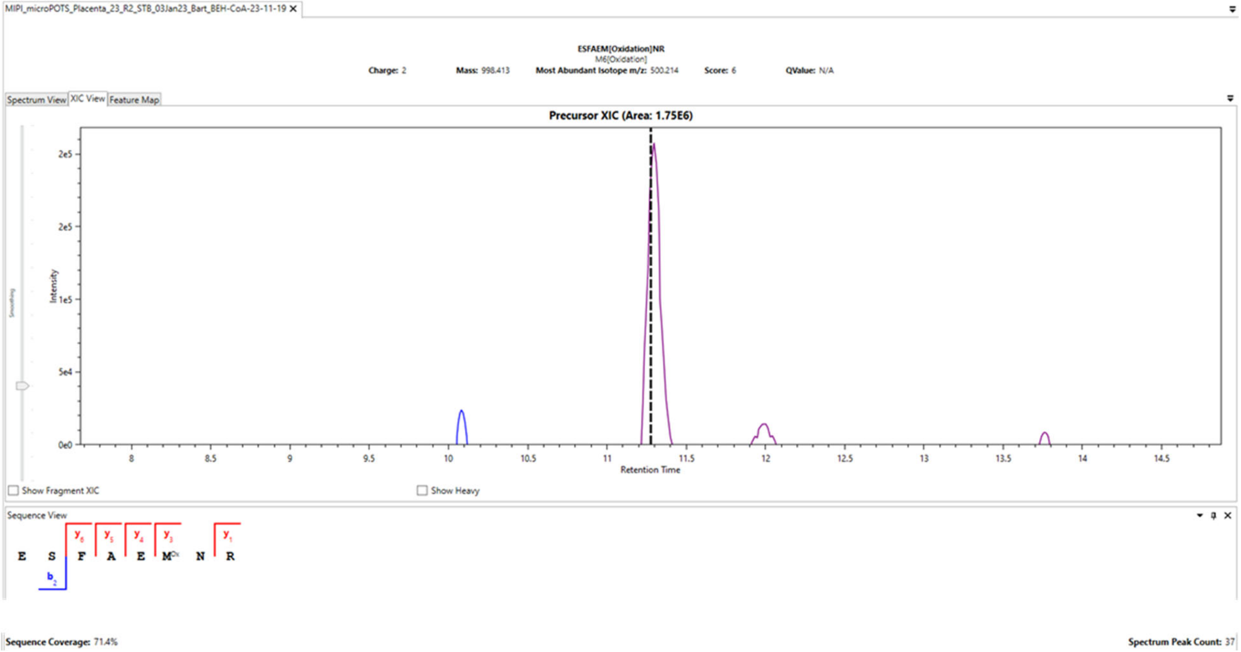

ix) Dataset: MIPI_microPOTS_Placenta_23_R5_STB_03Jan23_Bart_BEH-CoA-23-11-19; Scan Number for MS/MS: 7418

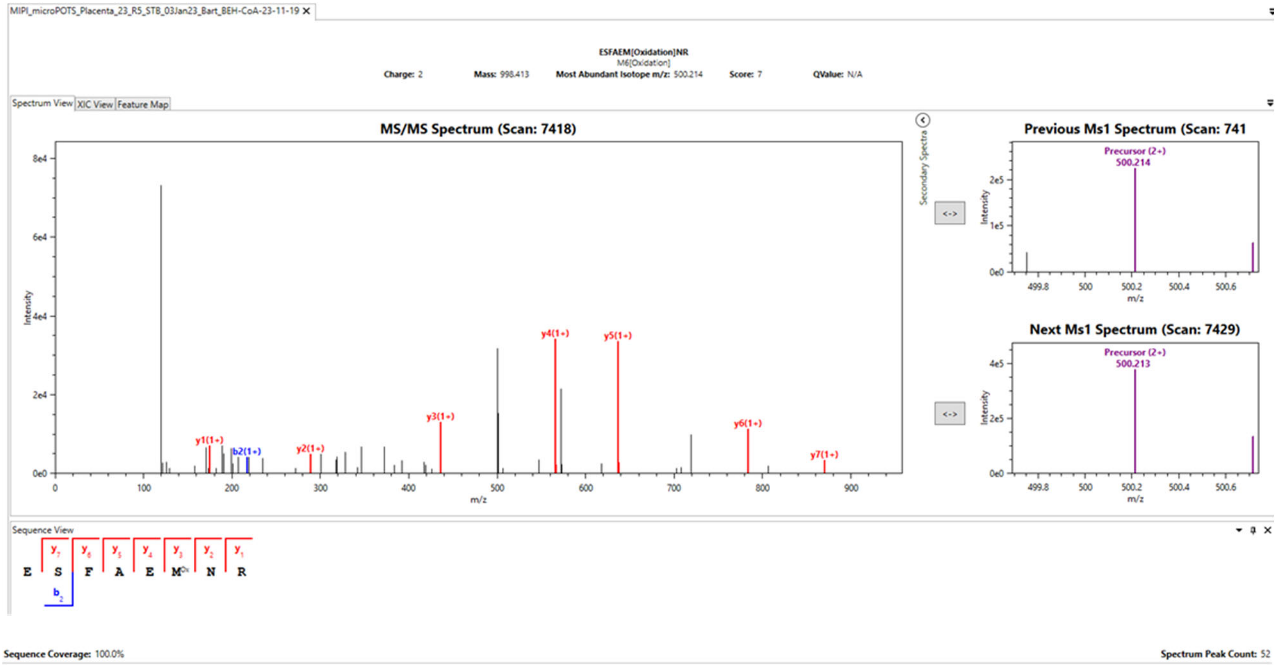

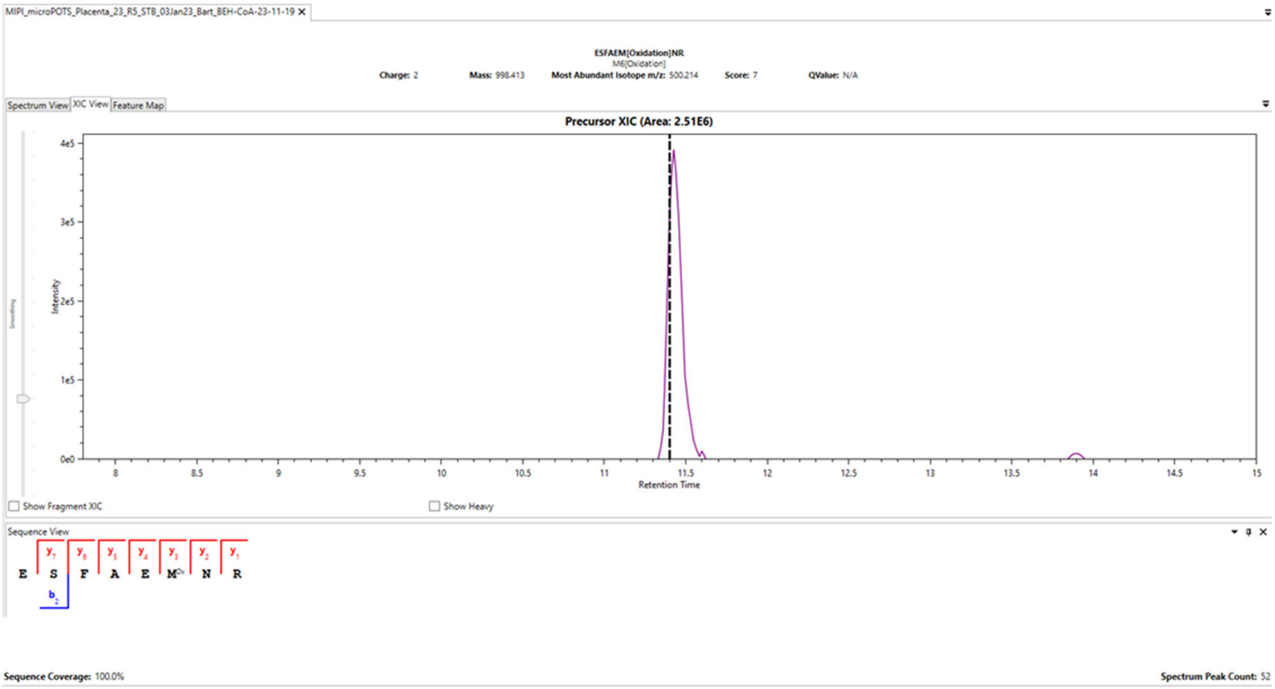

x) Dataset: MIPI_microPOTS_Placenta_23_R3_Core_03Jan23_Bart_BEH-CoA-23-11-19; Scan Number for MS/MS: 7402

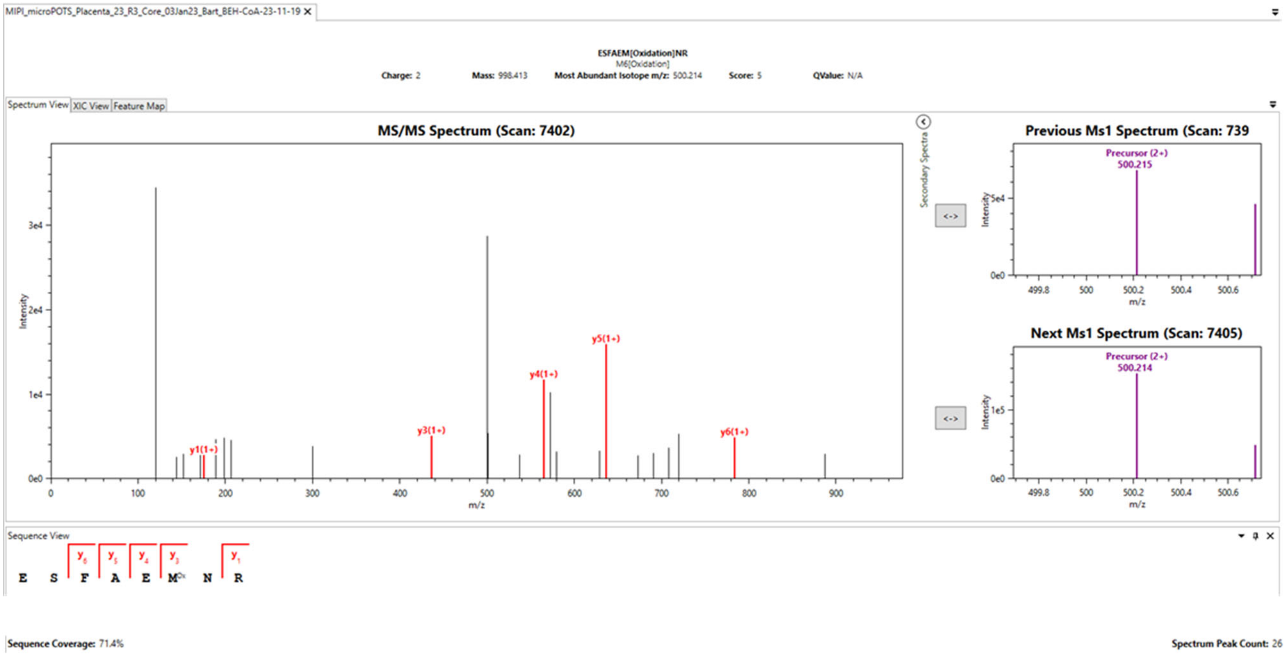

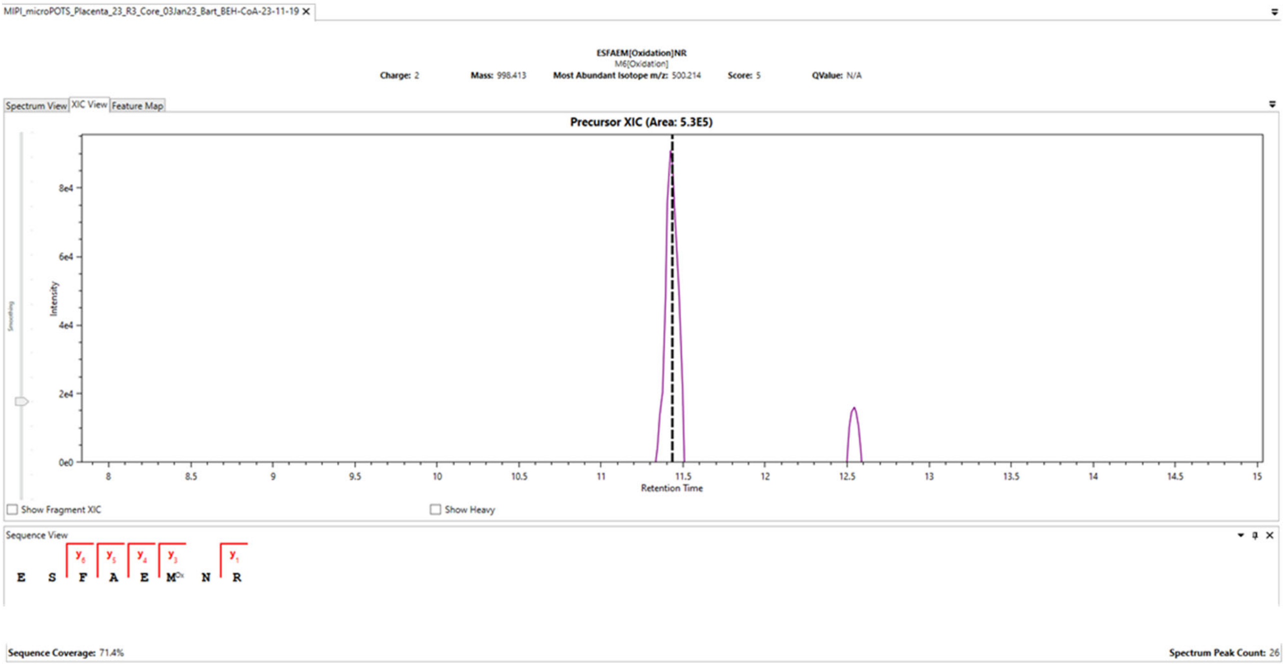

xi) Dataset: MIPI_microPOTS_Placenta_23_R1_STB_03Jan23_Bart_BEH-CoA-23-11-19; Scan Number for MS/MS: 7131

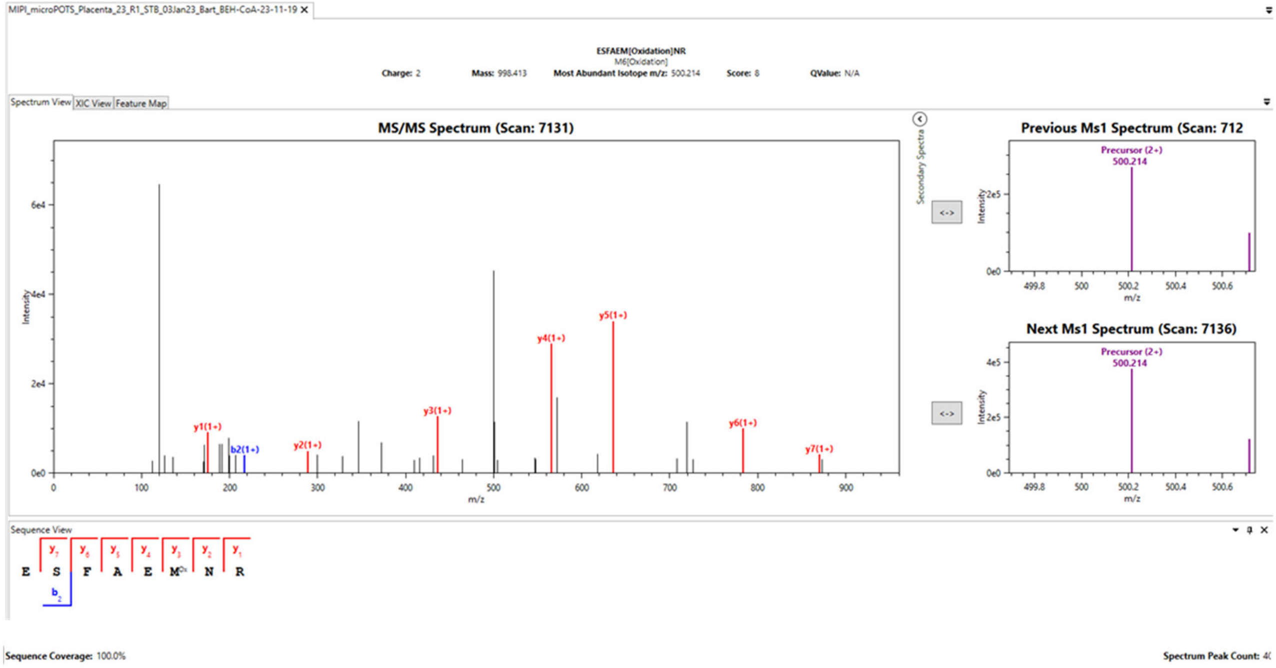

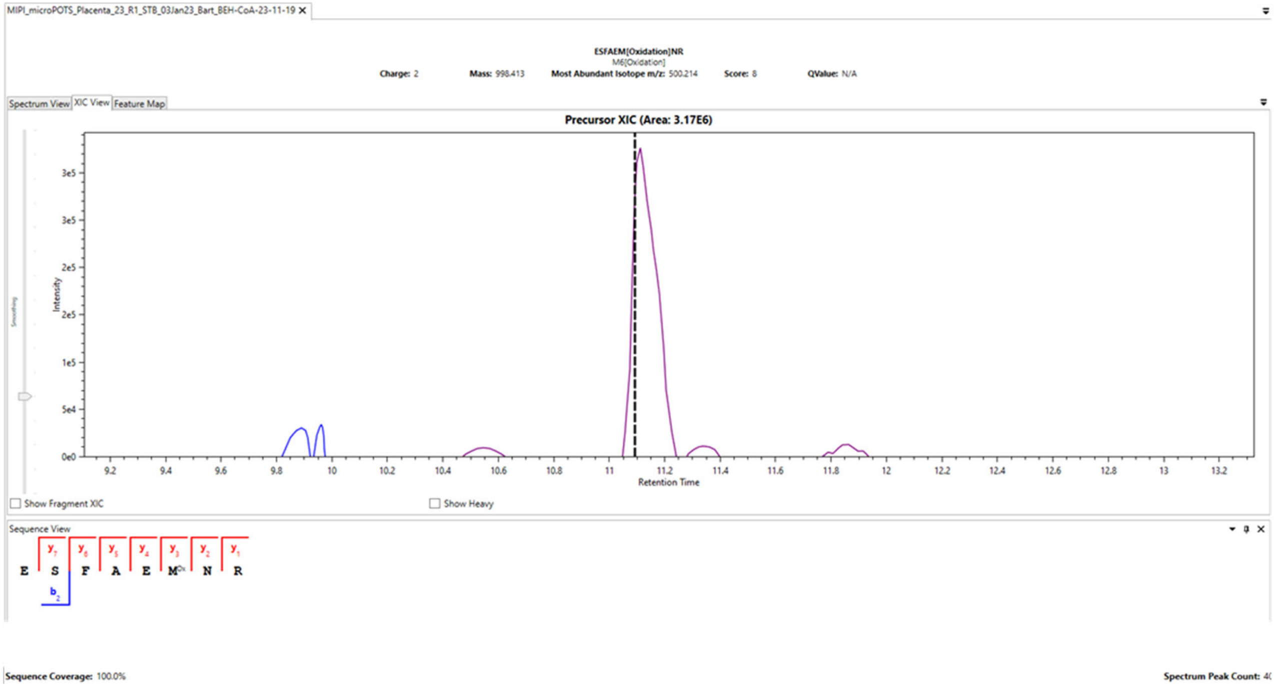

xii) Dataset: MIPI_microPOTS_Placenta_19_R1_STB_03Jan23_Bart_BEH-CoA-23-11-19; Scan Number for MS/MS: 7229

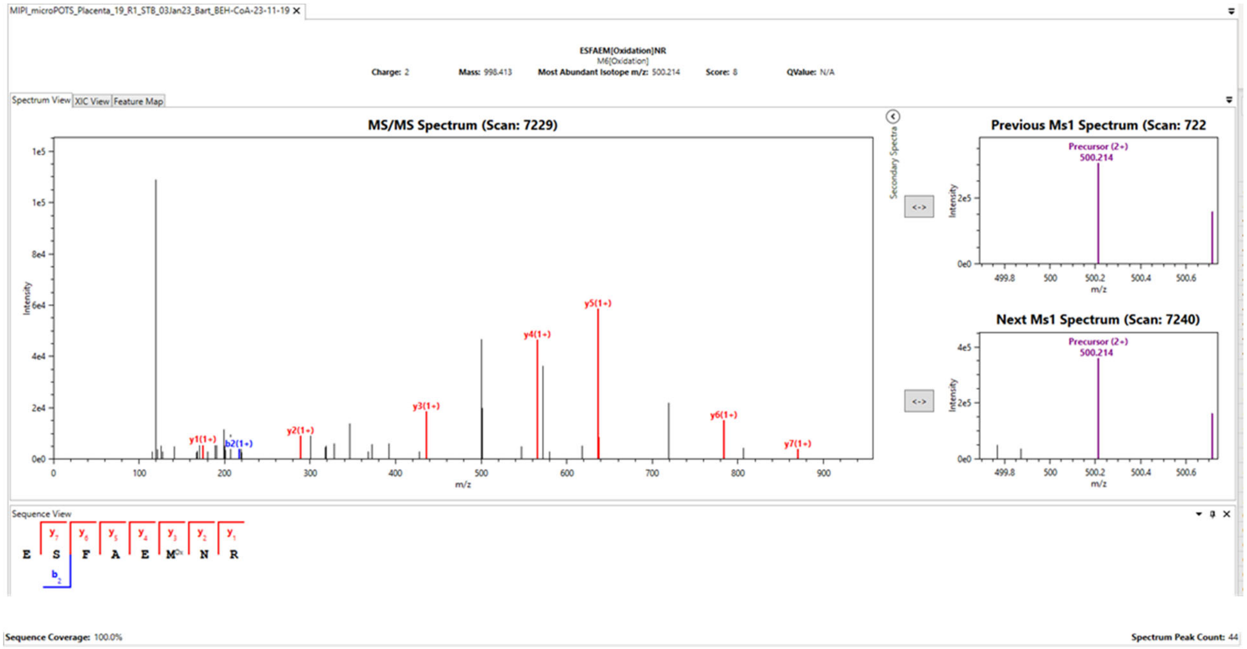

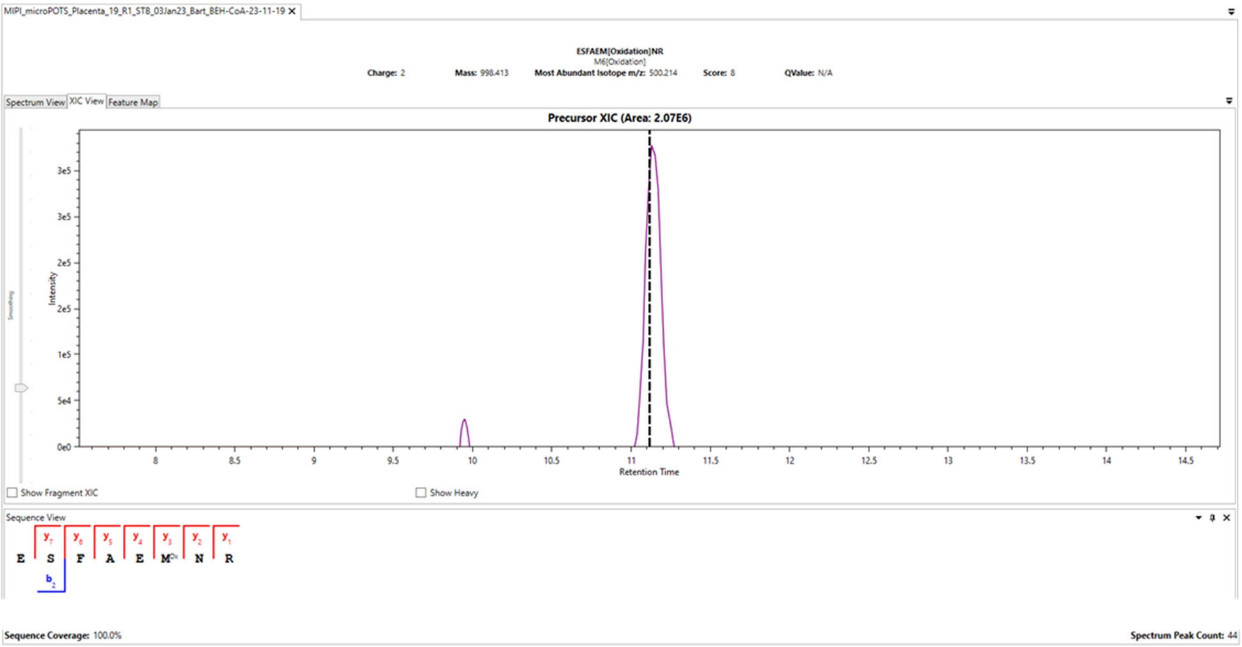

xiii) Dataset: MIPI_microPOTS_Placenta_19_R2_STB_03Jan23_Bart_BEH-CoA-23-11-19; Scan Number for MS/MS: 7112

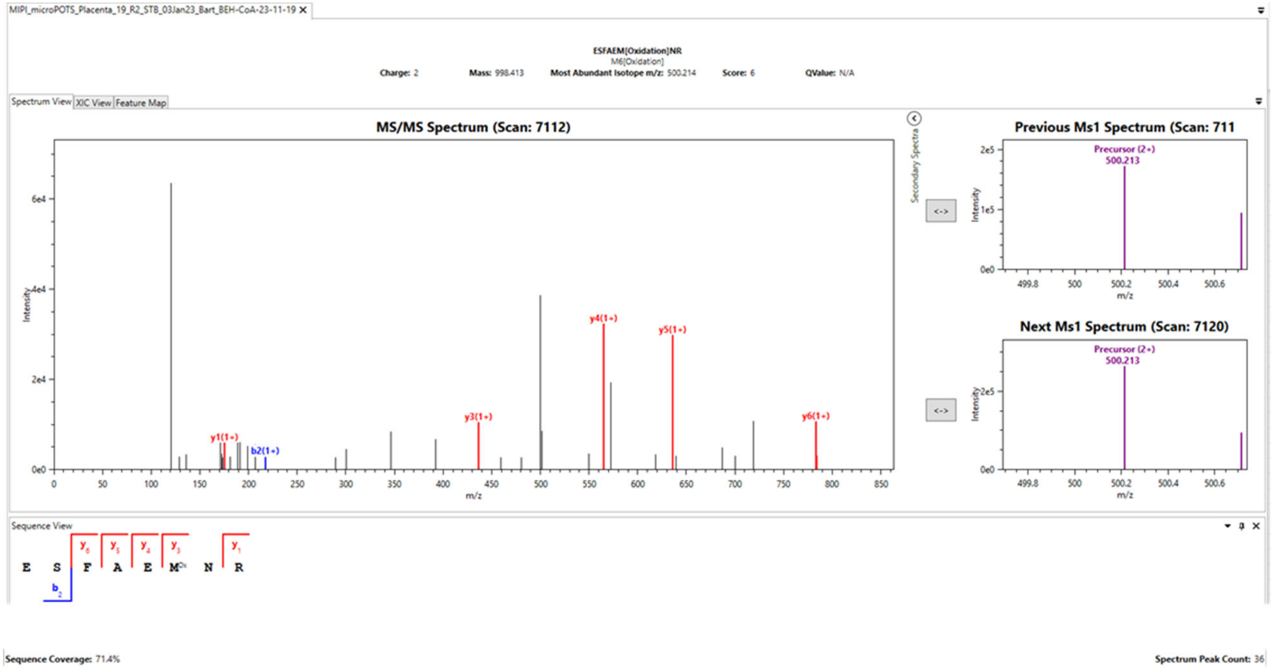

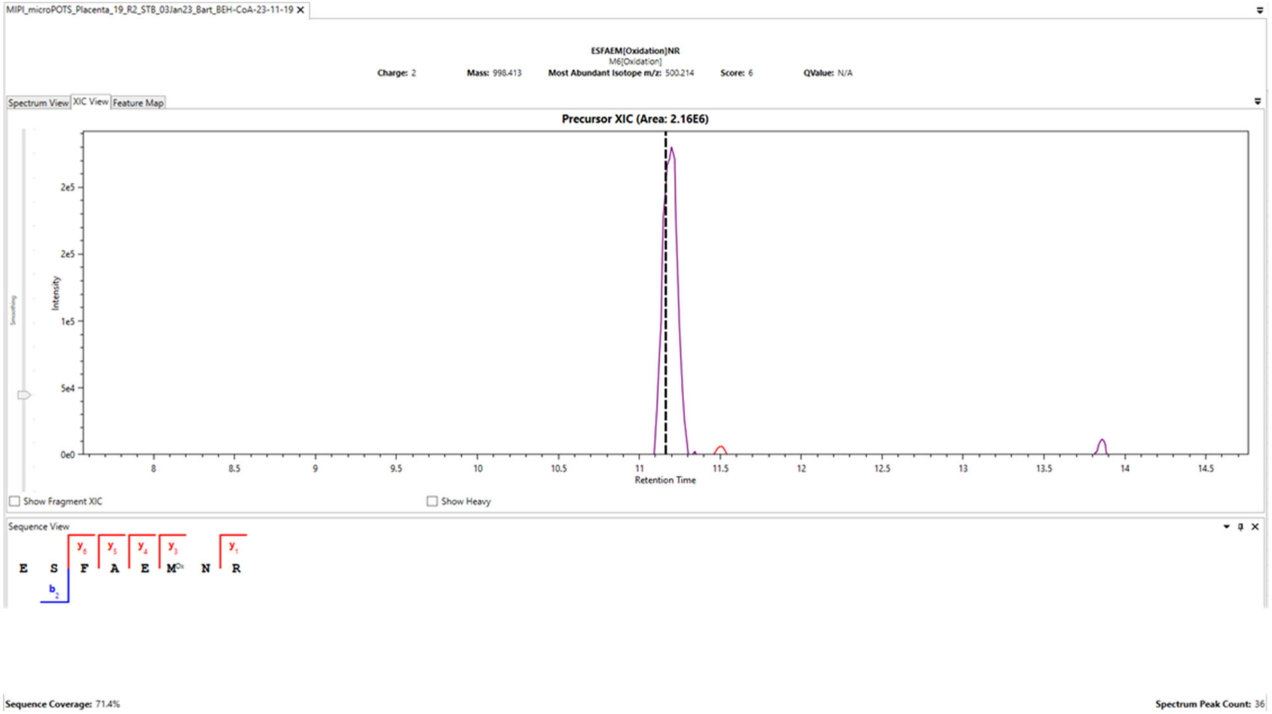

xiv) Dataset: MIPI_microPOTS_Placenta_19_R5_STB_03Jan23_Bart_BEH-CoA-23-11-19; Scan Number for MS/MS: 7273

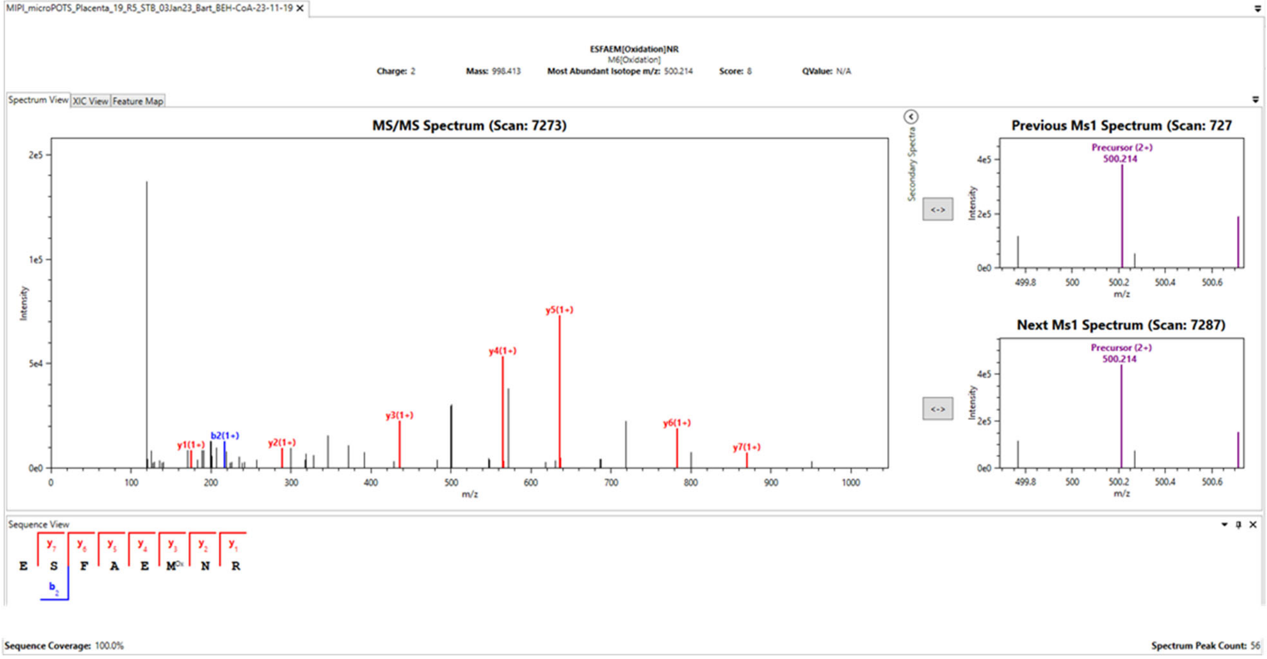

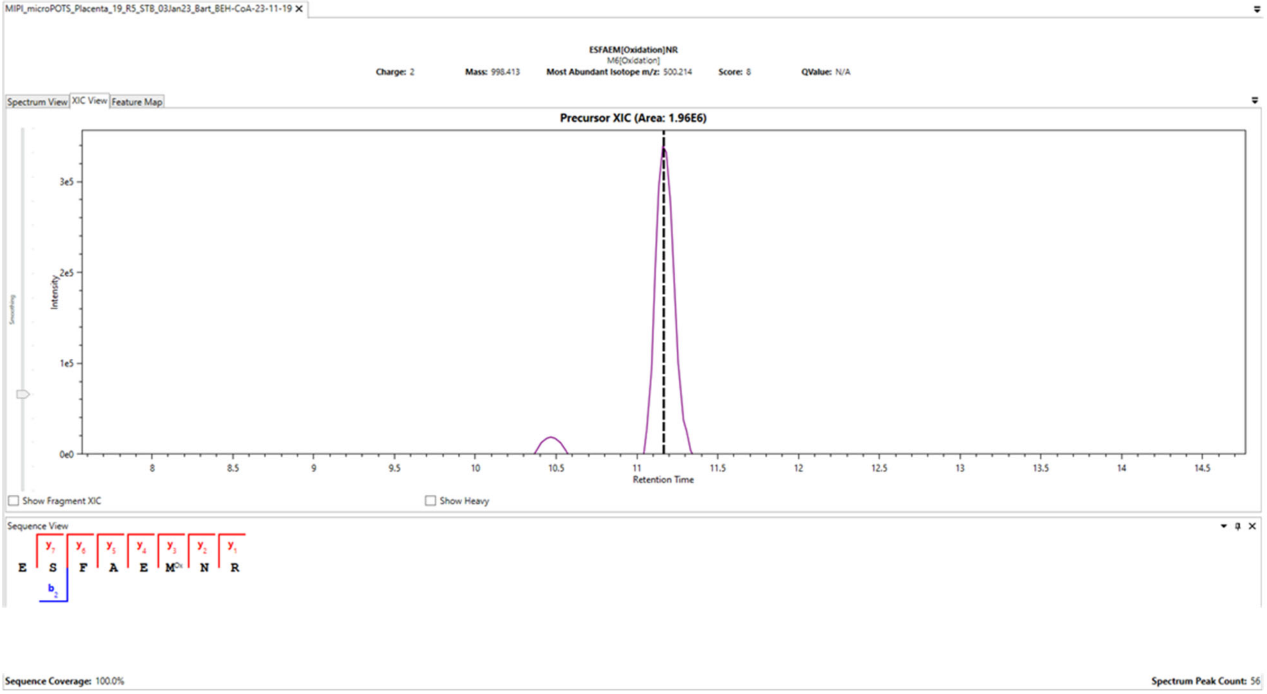

xv) Dataset: MIPI_microPOTS_Placenta_19_R3_STB_03Jan23_Bart_BEH-CoA-23-11-19; Scan Number for MS/MS: 7026

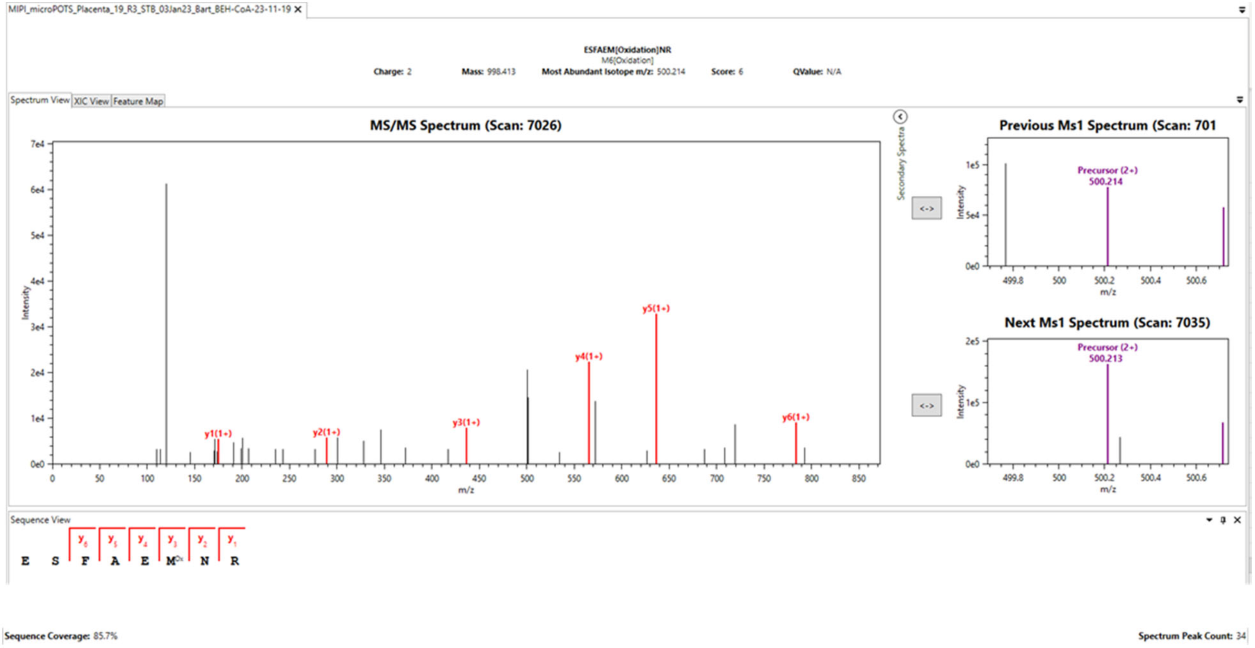

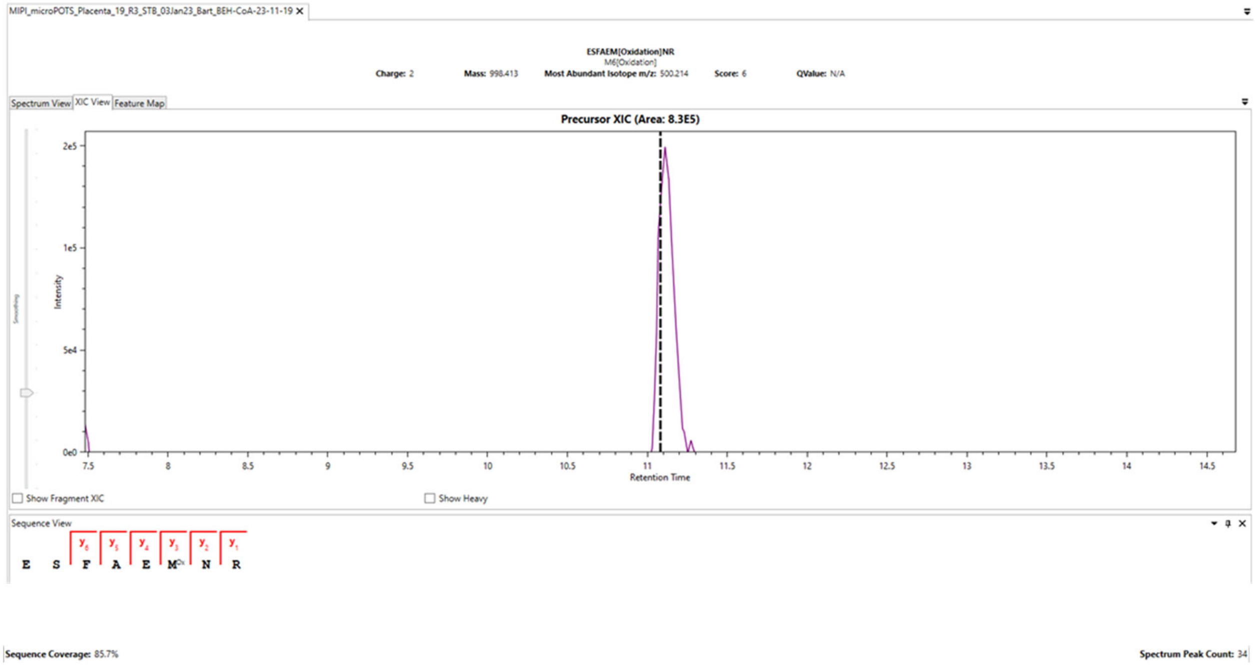

Supplementary Figure 2. Annotated mass spectrum of proteins identified with 1 peptide.
a) Protein sp|O00468|AGRIN_HUMAN, peptide: R.SIESTLDDLFR.N. b) Protein sp|O14975|S27A2_HUMAN, peptide: R.DETLTYAQVDR.R. c) Protein sp|O15269|SPTC1_HUMAN, peptide: R.VVVTVEQTEEELER.A. d) Protein sp|P20674|COX5A_HUMAN, peptide: R.LNDFASTVR.I. e) Protein sp|Q9BUT1|DHRS6_HUMAN, peptide: K.VIILTAAAQGIGQAAALAFAR.E. f) Protein sp|P23786|CPT2_HUMAN, peptide: K.SEYNDQLTR.A. g) Protein sp|P26572|MGAT1_HUMAN, peptide: R.LAQDAEVELER.Q. h) Protein sp|P13073|COX41_HUMAN, peptide K.ESFAEM*NR.G.

a)

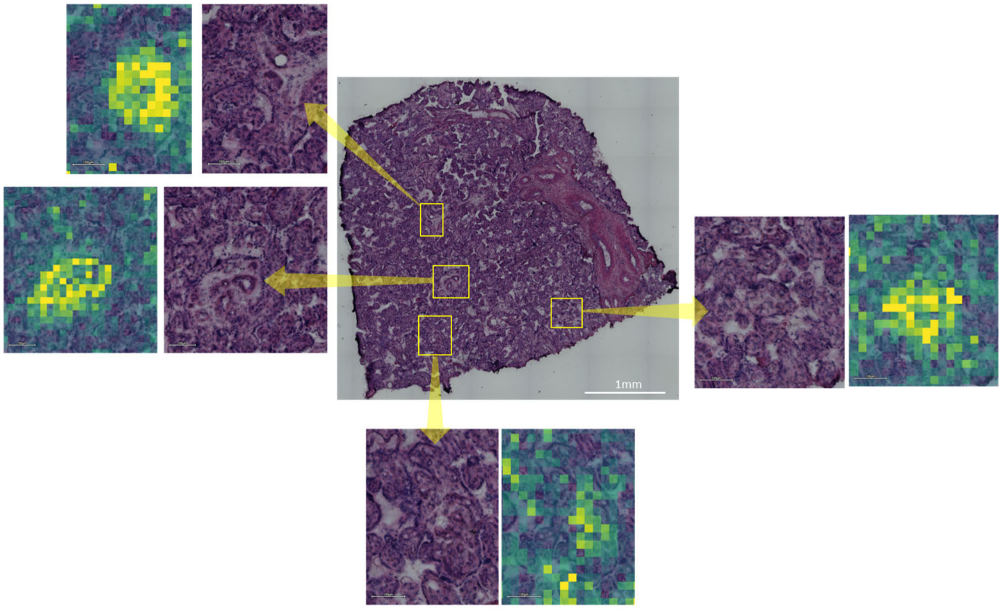

b)

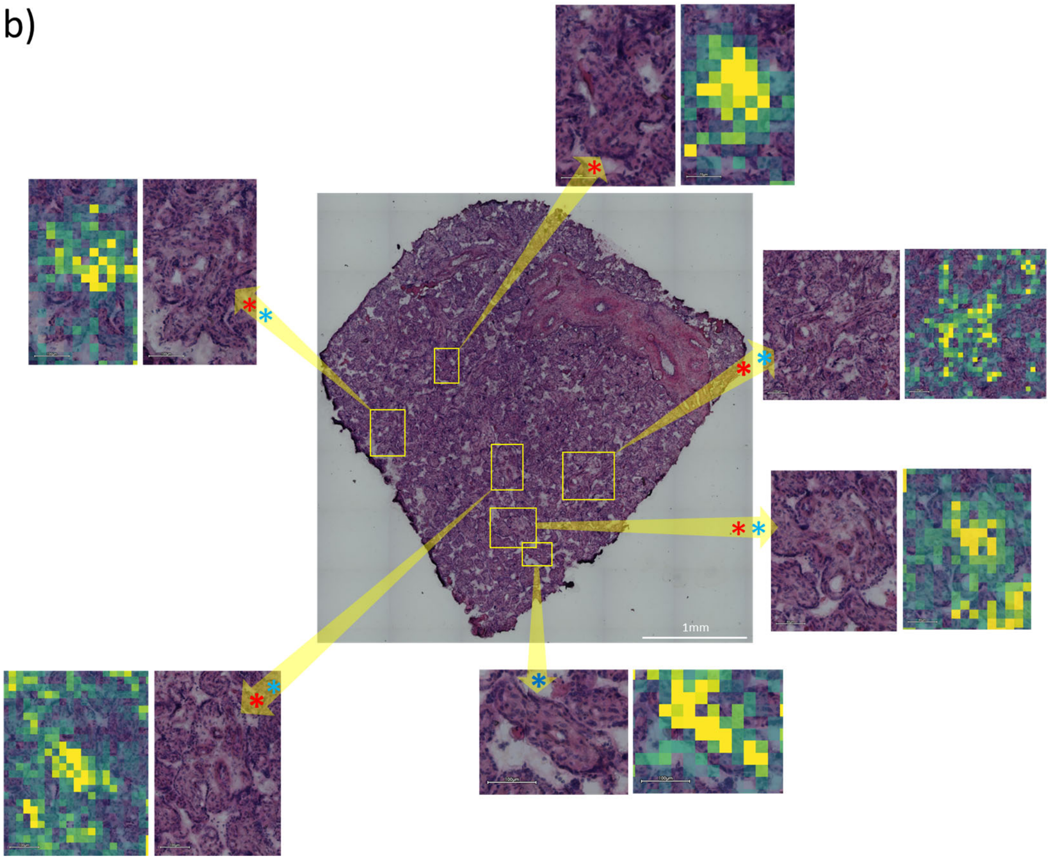

Supplementary Figure 3. Villous regions mapped for subsequent LCM collection. All mapped regions are zoomed and overlayed with ketone body ion images. a) Metabolomics section 18 with 4 marked regions collected from proteomics section 19. b) Metabolomics section 22 with 6 marked regions collected from 2 adjacent sections. Regions marked with a red asterisk were collected from proteomics section 23, while regions marked with a blue asterisk were collected from proteomics section 26.

a) GO term enrichment analysis

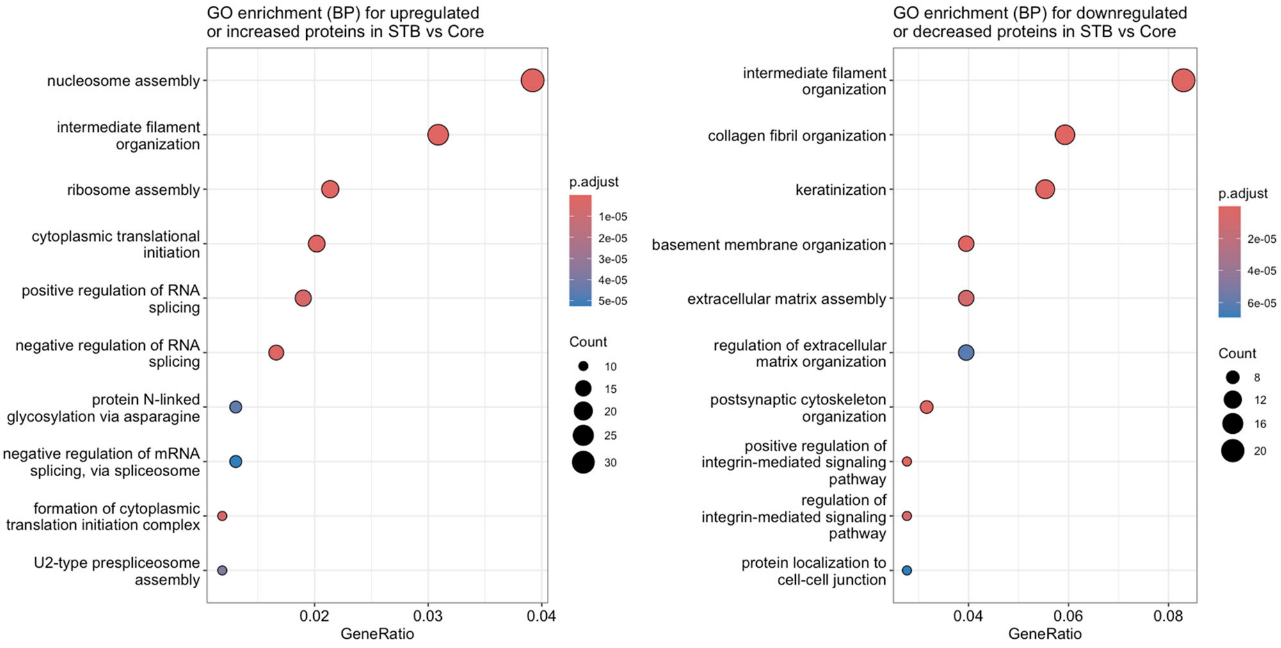

b) HumanCyc pathway enrichment analysis

Upregulated or increased proteins in STB

Enriched pathways	Raw p-value	Matches
**Generation of Precursor Metabolites and Energy**	7.22E-11	PDHB, PDHA1, DLD, TKT, TALDO1, ATP6V0D1, ATP6V1B2, ATP5PB, ATP5ME, ATP5MF, ATP5MG, ATP5PO, ATP5F1B, ATP5F1A, ACLY, GPD1, DLST, OGDH, IDH3A, SDHB, ACO2, NDUFA4, COX7A2, COX6C, COX5A, COX4I1, MT-CO2, NDUFA13, NDUFA9, NDUFS8, NDUFS3, NDUFS2, UQCRFS1, UQCRC2, UQCRB, HK1, PFKL
**Respiration**	3.52E-06	NDUFA4, COX7A2, COX6C, COX5A, COX4I1, MT-CO2, SDHB, NDUFA13, NDUFA9, NDUFS8, NDUFS3, NDUFS2, UQCRFS1, UQCRC2, UQCRB, DLD, DLST, OGDH
**Aerobic respiration I (cytochrome c)**	9.35E-05	NDUFA4, COX7A2, COX6C, COX5A, COX4I1, MT-CO2, SDHB, NDUFA13, NDUFA9, NDUFS8, NDUFS3, NDUFS2, UQCRFS1, UQCRC2, UQCRB
**Protein <i>N</i>-glycosylation initial phase (eukaryotic)**	**5.07E-04**	**STT3B, STT3A, DAD1, RPN2, RPN1, DDOST, DPM1**
**Hormone Biosynthesis**	**9.58E-05**	**CYP11A1, CYP19A1, HSD17B1, HSD3B1, HSD3B2, POR, HSD11B2, CTSD**
**TCA cycle**	**2.44E-03**	**DLD, DLST, OGDH, IDH3A, SDHB, ACO2**
**Fatty acid β-oxidation**	**0.0075**	**HADHA, SLC27A2, HADHB, HSD17B10**

Upregulated or increased proteins in core

Enriched pathways	Raw p-value	Matches
**Gluconeogenesis**	5.43E-06	GPI, MDH1, PGAM2, PGAM1, PGK1, GAPDH
**Glycolysis**	6.39E-05	GPI, PGAM2, PGAM1, PGK1, GAPDH
**Sugar Biosynthesis**	9.68E-05	UGDH, GPI, MDH1, PGAM2, PGAM1, PGK1, GAPDH
**Generation of Precursor Metabolites and Energy**	1.69E-04	ATP5PD, ATP5F1E, GAPDH, BDH2, ACAT1, MDH1, LDHA, GPI, PGAM2, PGAM1, PGK1
**Superpathway of conversion of glucose to acetyl CoA and entry into the TCA cycle**	2.43E-04	GPI, PGAM2, PGAM1, PGK1, GAPDH, MDH1
**Ketolysis**	**0.0048**	**ACAT1, BDH2**

c) KEGG module enrichment analysis

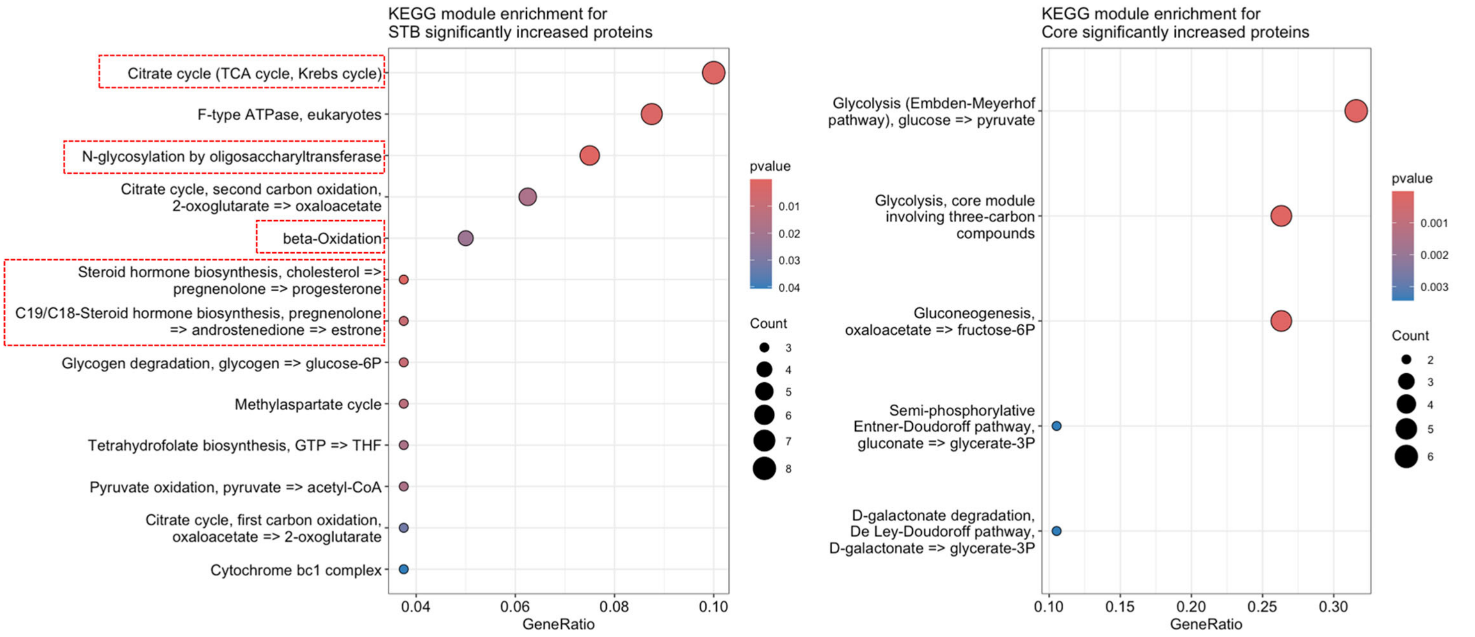

Supplementary Figure 4. Functional enrichment analyses results. a) Top 10 enrichment in GO biological pathways, both ANOVA and G-test results. b) HymanCyc pathway enrichment for significantly increased proteins using adjusted p-value for both, ANOVA and G-test, results. c) KEGG module enrichment for significantly increased proteins using adjusted p-value for both, ANOVA and G-test, results.

a) KEGG Fatty acid degradation - Reference pathway map00071

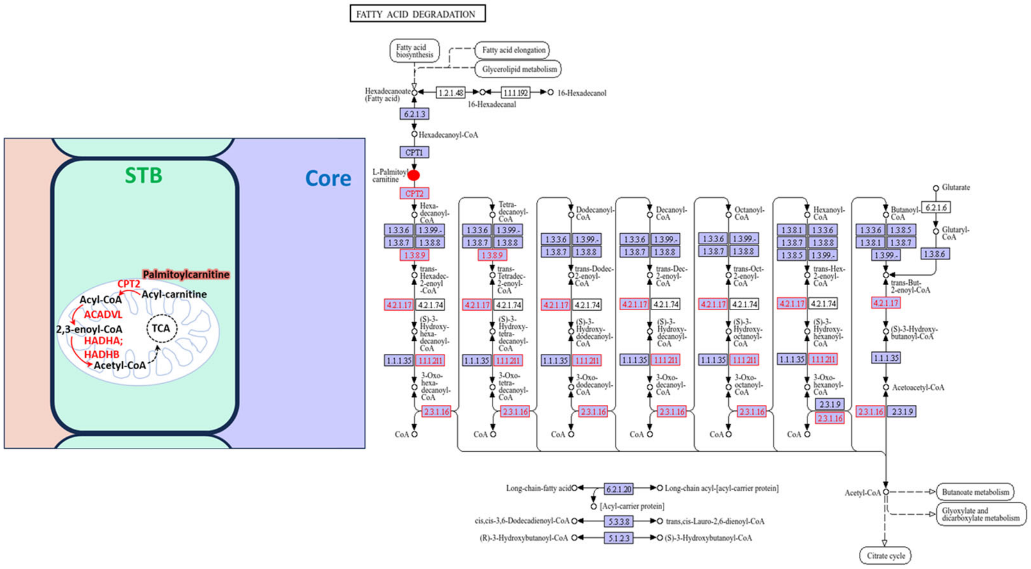

Palmitoylcarnitine detected in STB

400.3421 m/z = $[C_{23}H_{45}NO_4 + H]^+$

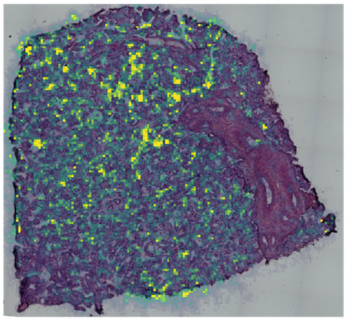

Proteins detected in STB

Protein	EC#	adj. p-value
ACADVL	1.3.8.9	4.76E-20
HADHA	4.2.1.17; 1.1.1.211	2.72E-17
HADHB	2.3.1.16	6.15E-06
CPT2	2.3.1.21	0.04 (p-value)

b) HumanCyc - Pathway ketolysis

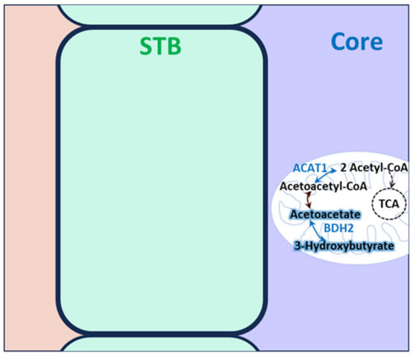

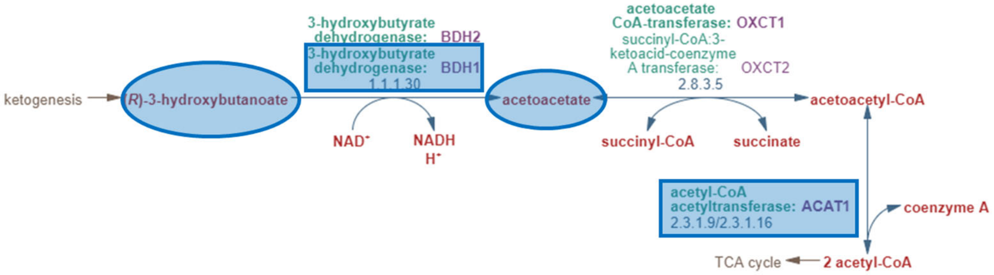

Proteins detected in CORE

Protein	EC#	adj. p-value
BDH2	1.1.1.30	0.012
ACAT1	2.3.1.9	0.001

3-Hydroxybutyrate (A.K.A. 3-hydroxybutanoate)

449.1434 m/z = $[C_4H_8O_3 + C_{18}N_2H_{22}Br]^+$

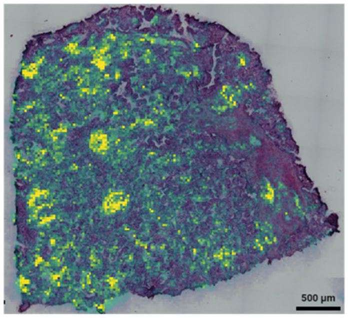

Acetoacetate

447.1278 m/z = $[C_4H_6O_3 + C_{18}N_2H_{22}Br]^+$

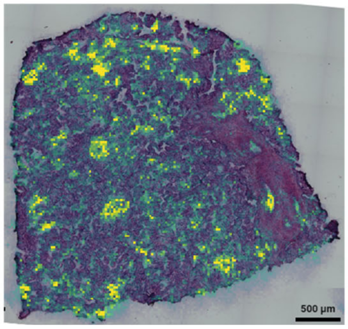

c) KEGG Steroid hormone biosynthesis - Reference pathway map00140

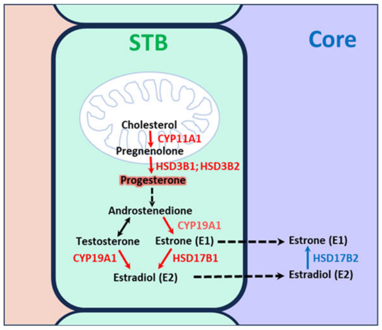

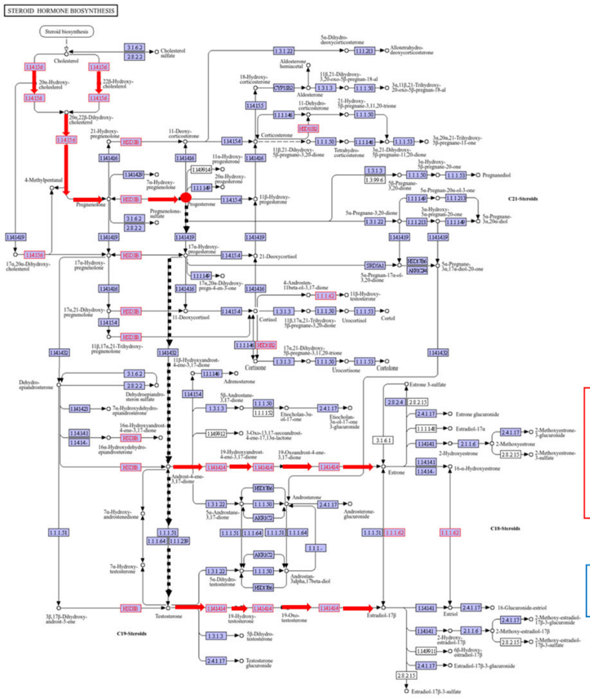

Progesterone

$[C_{21}H_{30}O_2 + C_{18}H_{22}N_2Br]^+$ = 659.3207 m/z

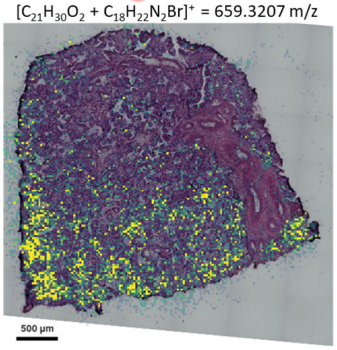

Proteins detected in STB

Protein	EC#	adj. p-value
CYP11A1	1.14.15.6	2.25E−41
HSD3B1	1.1.1.145	3.99E-36
HSD3B2	1.1.1.145	1.09E-32
CYP19A1	1.14.14.14	4.29E-37
HSD17B1	1.1.1.62	1.04E-9

Protein detected in CORE

Protein	EC#	adj. p-value
HSD17B2	1.1.1.62	8.85E-7

d) KEGG Sphingolipid metabolism - Reference pathway map00600

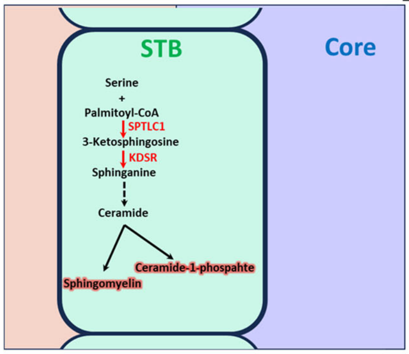

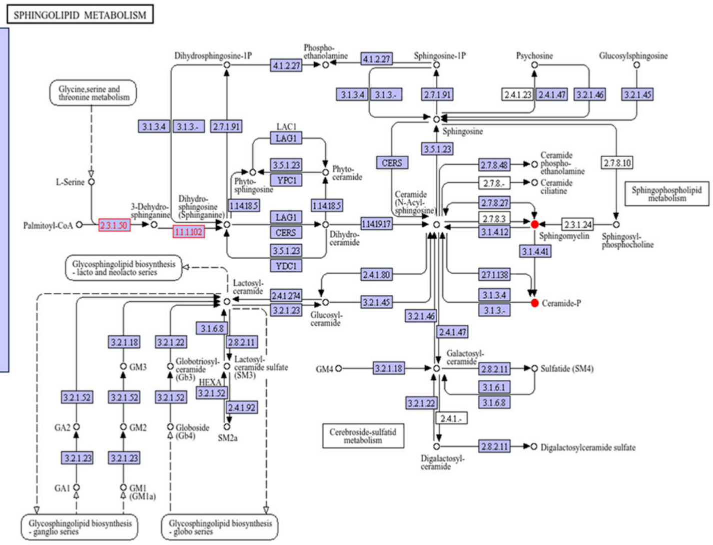

Proteins detected in STB

Protein	EC#	adj. p-value
SPTLC1	2.3.1.50	0.04
KDSR	1.1.1.102	0.0003

Sphingomyelin

SM(d18:1/24:0)/SM(d18:0/24:1)

$[C_{47}H_{95}N_2O_6P+Na]^+$ = 837.6820 m/z

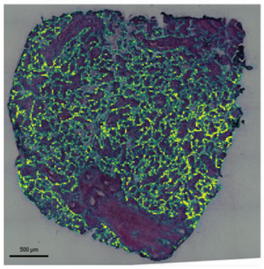

Ceramide-1-phospahte

CerP(d18:1/26:1)

$[C_{44}H_{86}NO_6P + Na]^+$= 778.6085 m/z

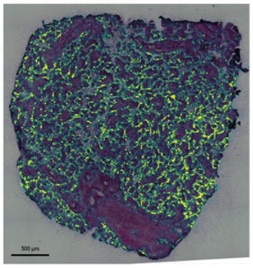

Supplementary Figure 5. Spatial multi-omics data integration. a) Reconstructed model of fatty acid degradation in villous STB subregion. b) Proposed ketone body oxidation pathway in the villous core. c) Reconstructed estrogen synthesis pathway in STB. d) Reconstructed de novo synthesis pathway of ceramide as part of sphingolipid metabolism in STB.

Supplementary Statistical Methods

Software and Code

Analyses were performed in R version 4.3.2. The following packages were used:

Package	Use	Reference
dplyr	General data processing	[1]
ggplot2	Create figures	[2]
pmartR	Processing, normalizing lipid data	[3, 4]
lme4	Fitting mixed effect models	[5]
partR2	partitioning variance components of data	[6]

Data Summary

Proteomic samples were run and analyzed for three tissues. There were 27 samples from two different subregion types. **Table 1** gives the number of samples per tissue and sample type. A total of 12,666 peptides mapping to 2,821 unique proteins were identified, with 77% missing observations.

Table 1. Number of samples per tissue and subregion type

Tissue	Core	STB
19	3	4
23	5	5
26	5	5

Data Preprocessing

Data Transformation

Peak intensities were log2 transformed, and all missing intensity values were converted to NA values. Distribution of log2 peak intensities for each sample are shown in **Figure 1**.

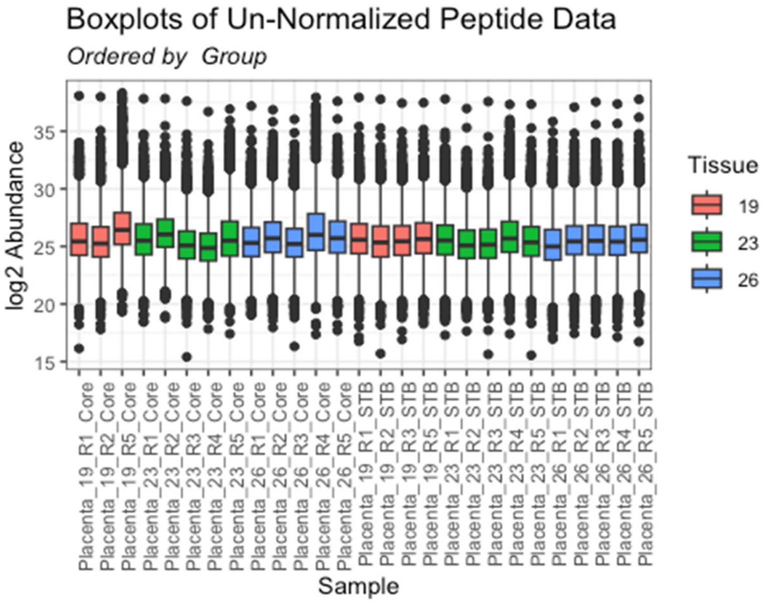

Figure 1. Boxplots of log2 peptide intensity distributions for each sample

Peptide Filter

Peptides without enough observations to conduct a statistical test, quantitative or qualitative, were filtered from the data[7]. A total of 5,874 peptides mapping to 1,048 proteins were filtered from the dataset. The resulting data consisted of 6,792 peptides mapping to 1,773 unique proteins.

Sample Outlier Identification

A robust Mahalanobis distance based on peptide abundance vectors (rMd-PAV) was calculated to identify potential sample outliers in the data[8]. This distance is calculated based on five metrics: average correlation with samples in the same group, skewness of peptide abundance distribution, kurtosis of peptide abundance distribution, median absolute deviation (MAD), and proportion of missing observations. A p-value corresponding to a test, where the null hypothesis is that the sample is not an outlier, can then be calculated based on the observed distance measure.

Figure 2 shows the log2 rMd-PAV score for each sample. The horizontal line corresponds to the log2 score associated with a p-value of 0.0001 (the recommended threshold), and samples falling above the line are labeled as potential outliers. A total of 2 samples were flagged as potential outlier samples.

Figure 3 shows the value of the five metrics for each potential outlier relative to the distribution of values observed across all samples. Placenta_26_R4_Core showed significantly lower correlation and higher MAD compared to all other samples and was removed from the dataset.

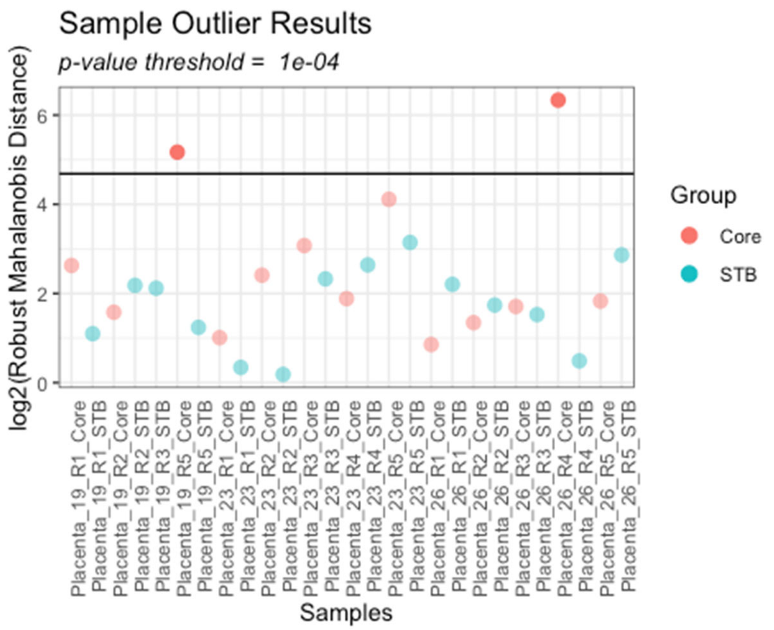

Figure 2. Log2 rMd scores for each sample colored by group. Solid black line corresponds to a log2 rMd score corresponding to p-value = 0.0001. Samples above black line are flagged as potential outliers.

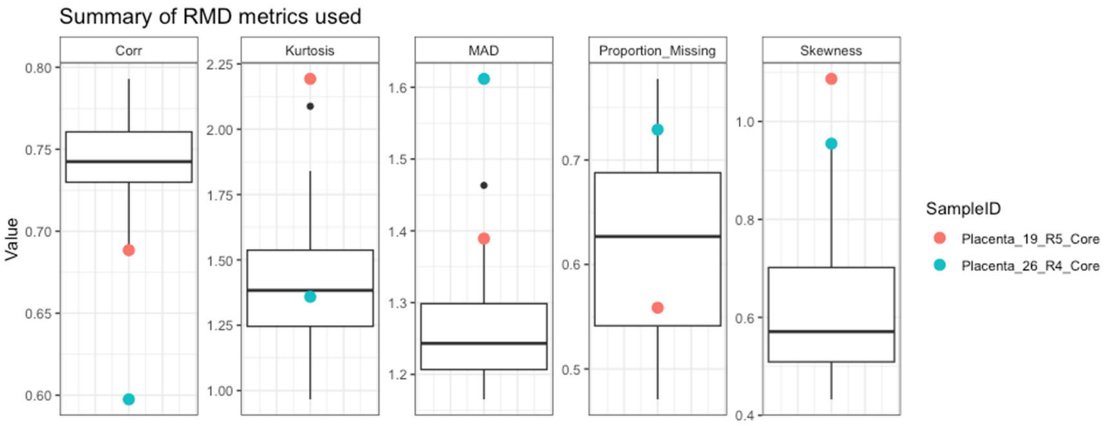

Figure 3. Distribution of values for each of the five metrics used in rMD-PAV across all samples. Each set of boxplots shows the value for the sample of concern.

Normalization

The Statistical Procedure for the Analyses of peptide abundance Normalization Strategies (SPANS) was implemented to determine the optimal normalization method without introducing bias into the data[9]. **Figure 4** shows the SPANS scores for methods passing initial checks; methods not scored did not pass the initial check for viability. The optimal normalization method of median centering was implemented to normalize the data. **Figure 5** shows the boxplots of the normalized log2 transformed abundance values by sample, across all peptides.

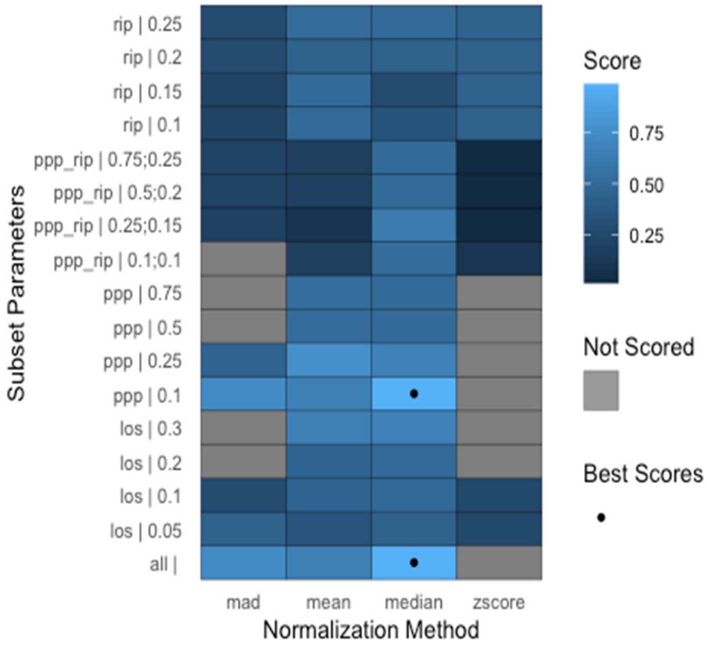

Figure 4. Results from SPANS algorithm to determine optimal normalization method.

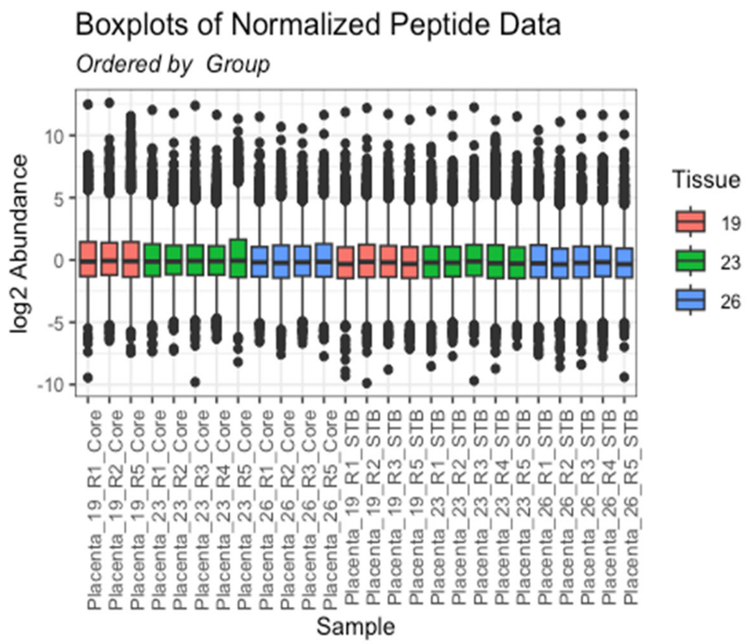

Figure 5. Boxplots of log2 global median normalized peptide data

The final peptide-level dataset used consisted of 6792 peptides mapping to 1,773 unique proteins. Sequential projection pursuit principal component analysis (PCA) was run[10]; this method provides the benefit that missing data does not need to be imputed for the algorithm to run. **Figure 6** shows the first two principal component scores for each sample with points colored by condition.

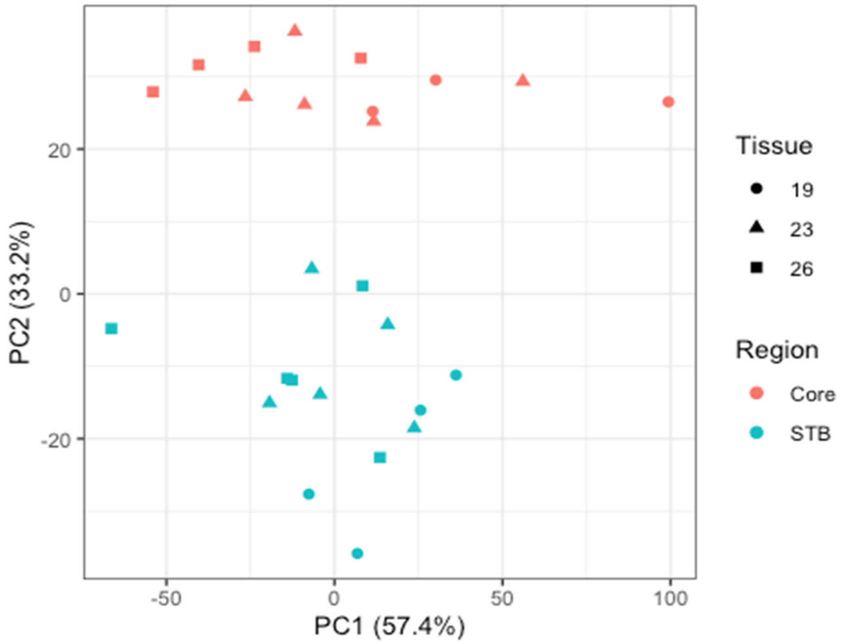

Figure 6. Scores for the first two principal components, based on peptide abundance profiles, for each sample with points colored by subregion type.

Variability Partitioning

Inter- and Intra-Section Variability

The intra-section variability and inter-section variability were considered in terms of two outcomes: peptide abundance and peptide detection. For each peptide, two models were fit: 1) a linear mixed effect model with abundance as the response variable with a fixed effect for subregion type and a random effect for tissue section, and 2) a generalized linear mixed effects model with peptide detection as the response variable was fit to the data with a conditional binomial distribution with a fixed effect for subregion type and a random effect for tissue section.

For each peptide and model, the variability of measurements, after accounting for subregion type, was quantified using the partR2 package[6]. The percent variability explained (R^2) can be attributed to the within section variability and the between section variability (while accounting for subregion type); lower R^2 means more consistent measurements. **Figure 7** shows the distribution of R^2 for within tissue measurements - R^2 for between tissue measurements, for the abundance and detection models, where negative values indicate more consist measurements within a tissue. A majority of peptides show consistency in measurements within a tissue, while accounting for subregion type.

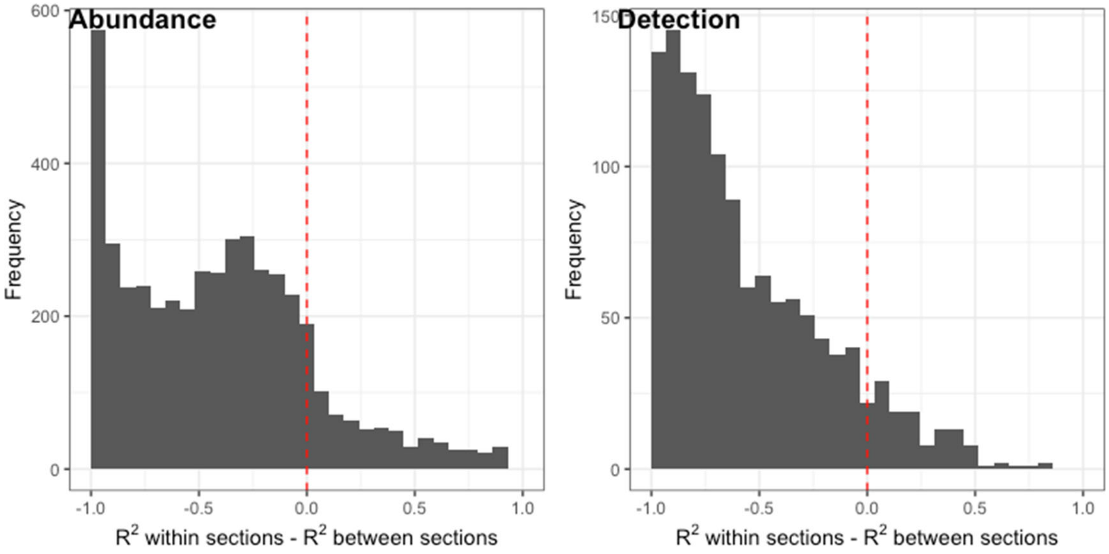

Figure 7. Distribution of R^2 for within tissue measurements - R^2 for between tissue measurements, for the abundance and detection models, where negative values indicate more consistent measurements within a tissue

Sample to Sample Correlation

Pearson's correlation of sample abundance profiles were calculated. **Figure 8** gives a correlation heatmap with samples ordered by tissue, replicate pair, and subregion. There is very little clustering of samples by replicate pair. **Figure 9** gives the correlation heatmap with samples ordered by subregion, tissue, and replicate. From this plot, it is clear that the primary source of sample similarity is the subregion type (STB or Core).

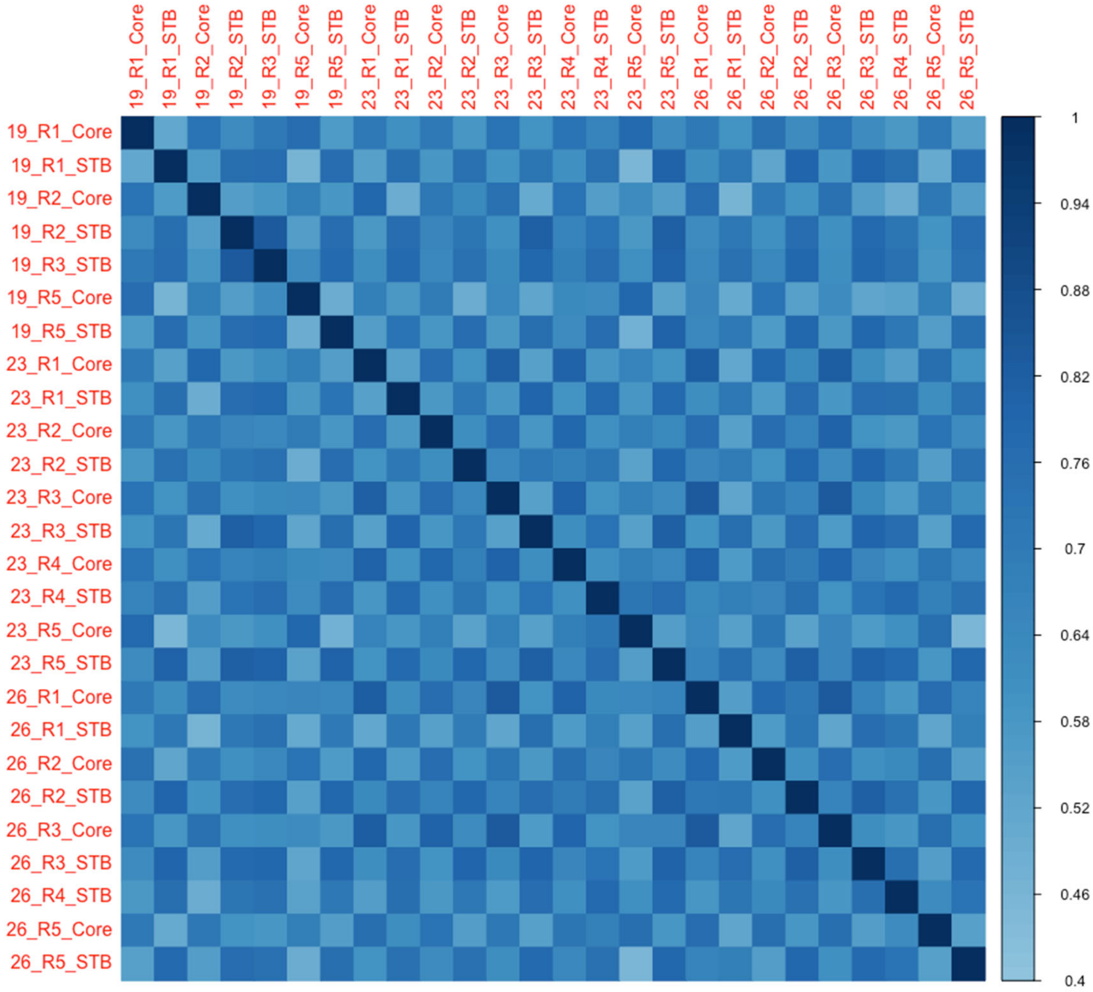

Figure 8. Correlation heatmap of sample-to-sample correlation, ordered by tissue, replicate, and subregion.

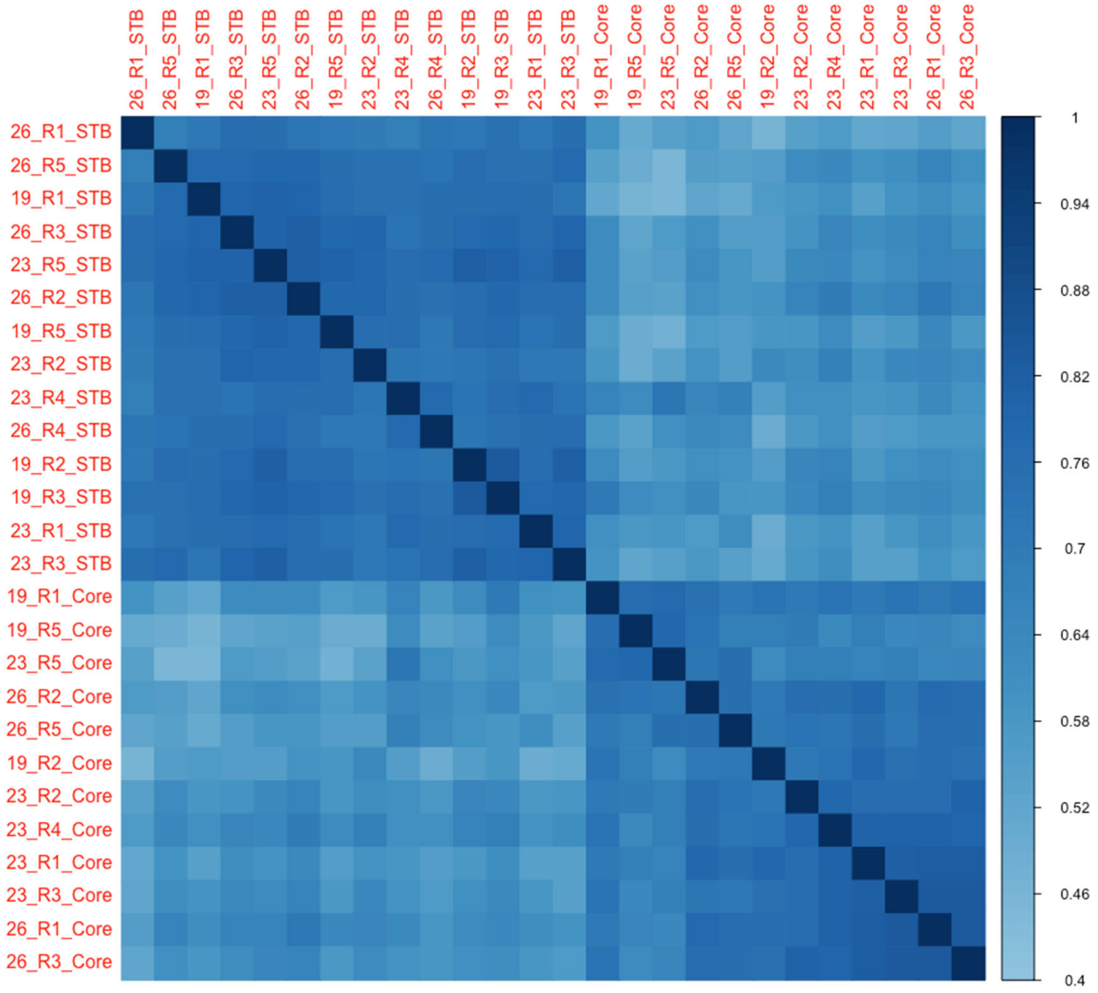

Figure 9. Correlation heatmap of sample-to-sample correlation, ordered by subregion (STB or Core), tissue, and replicate.

Statistical Analysis

For each protein, data for all peptides mapping to the protein were analyzed simultaneously. A linear mixed effects model was fit to the data, for each protein, with a fixed effect for subregion type and random effects for peptide and tissue section. A test for a difference in mean abundance between STB and Core subregions was conducted while accounting for the non-independence of peptide and tissue section. Additionally, a generalized linear mixed effects model was fit to the data with a conditional binomial distribution. The detection of a peptide of a peptide was coded as a 0/1 as the dependent variable, and them model included a fixed effect for subregion type and random effects for peptide and tissue section. A χ^2 likelihood ratio test was conducted to test for a difference in the probability of observing a protein while accounting for the non-independence of peptide and tissue section. A Benjamini-Hochberg[11] was used to correct for multiple comparisons.

Model Assumptions

For each protein, two models were fit to the data.

1. Abundance model: When modeling the abundance response variable, a linear mixed effects model was used. Tissue subregion was included as a fixed effect. This model assumes: (a) error variances are normally distributed, (b) error variances are independent, and (c) variances are homoskedastic. Because multiple samples were taken from each tissue section and each protein (in almost all cases) has multiple peptides being analyzed, condition (b) does not hold. Thus, peptide and tissue section were included in the model to properly partition variation and ensure a valid statistical comparison of means from tissue subregion. Assumptions (a) and (c) are commonly assumed to be valid for log transformed mass spectrometry data.

2. Detection model: When modeling the probability of detecting a given protein, a generalized linear mixed effects model, with a conditional binomial distribution and a logit link function was used. Tissue subregion was included as a fixed effect. This model assumes: (a) error variances are independent. Because multiple samples were taken from each tissue section and each protein (in almost all cases) has multiple peptides being analyzed, condition (a) does not hold. Thus, peptide and tissue section were included in the model to properly partition variation and ensure a valid statistical comparison of means from tissue subregion.

Statistical Test Results

Figure 10 shows the number of significant proteins for each test by direction of change. **Figure 11** gives a volcano plot (-log10 adjusted p-value vs log2 fold change) for the abundance-based model. **Figure 12** gives a modification of a volcano plot where we define the odds ratio as OR_p for protein p. We then define the relative odds ratio, ROR_p as: $ROR_p = OR_p$ if $OR_p > 1$, else $ROR_p = -1 * OR_p$ if $OR_p < 1$.

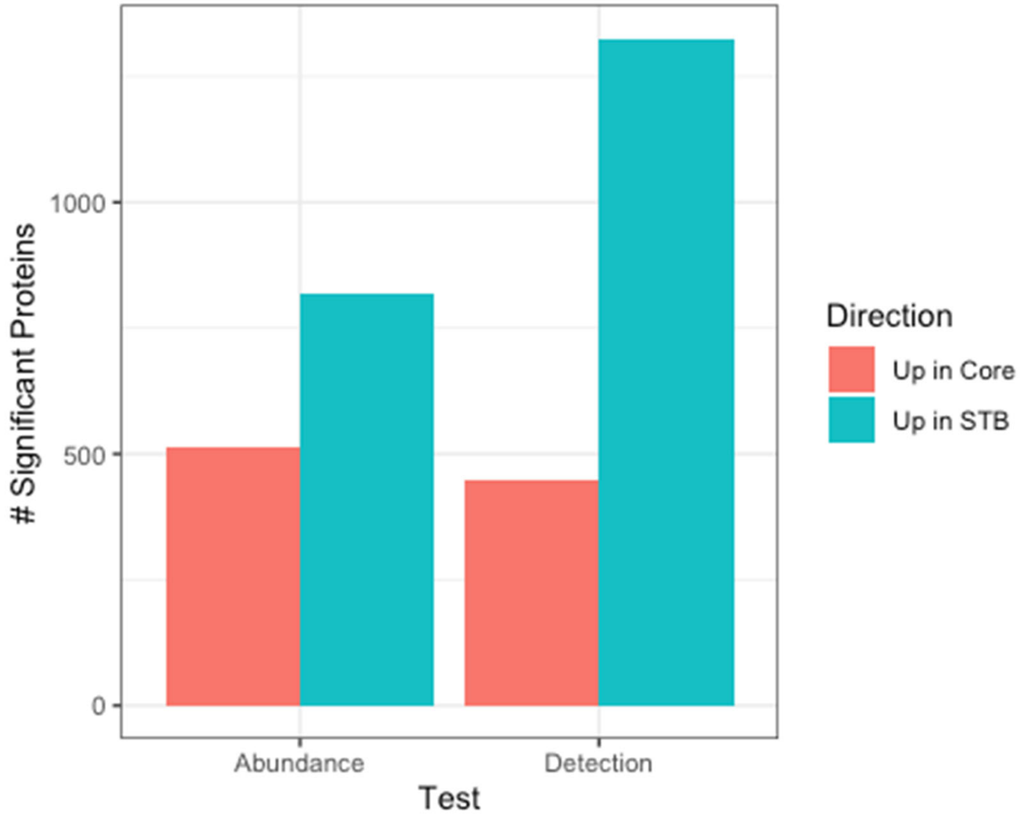

Figure 10. Number of significant proteins, with an adjusted p-value < 0.05, for each test by direction of change.

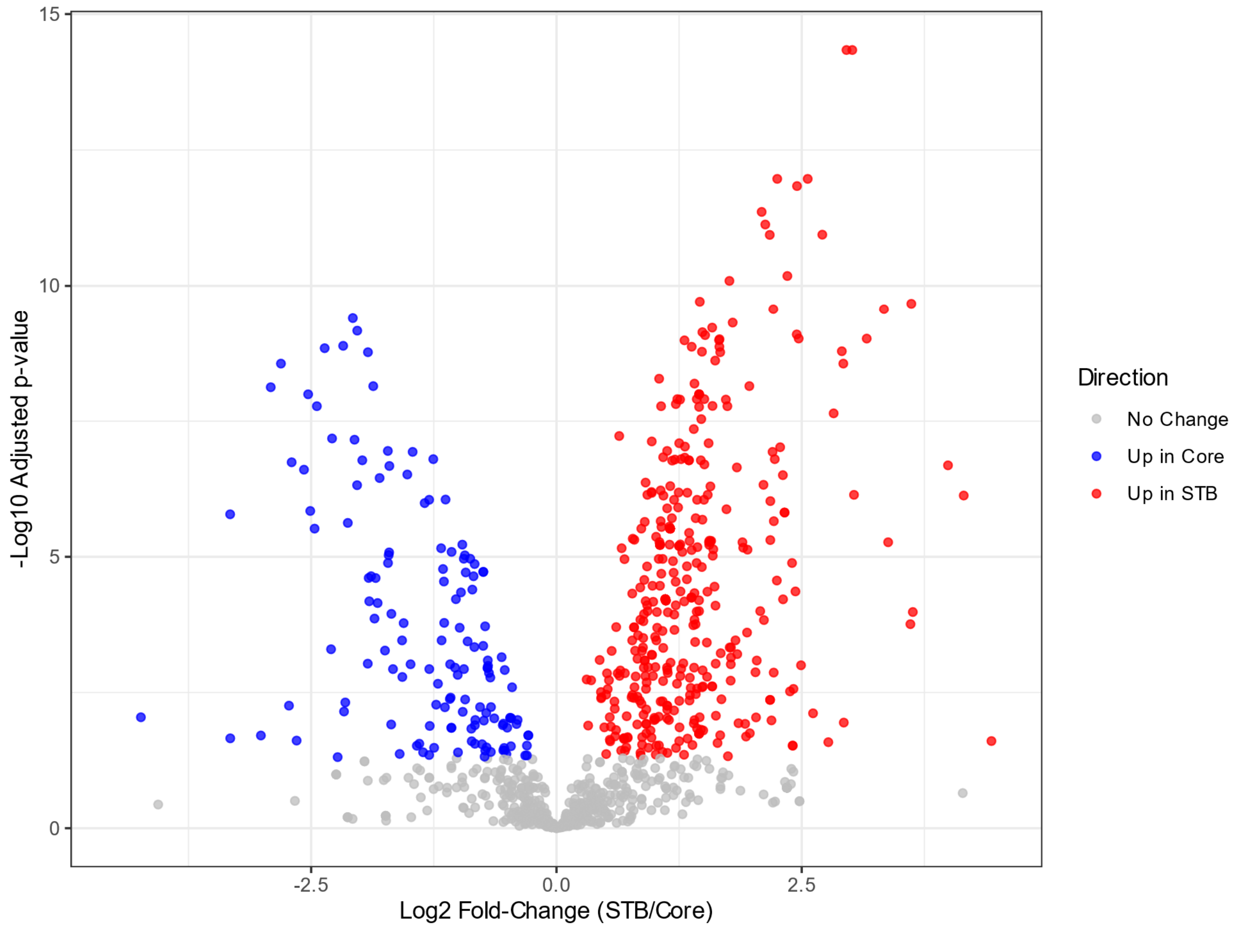

Figure 11. Volcano plot for the abundance-based model comparing tissue subregion means for each protein. Source data are provided as a Source Data file.

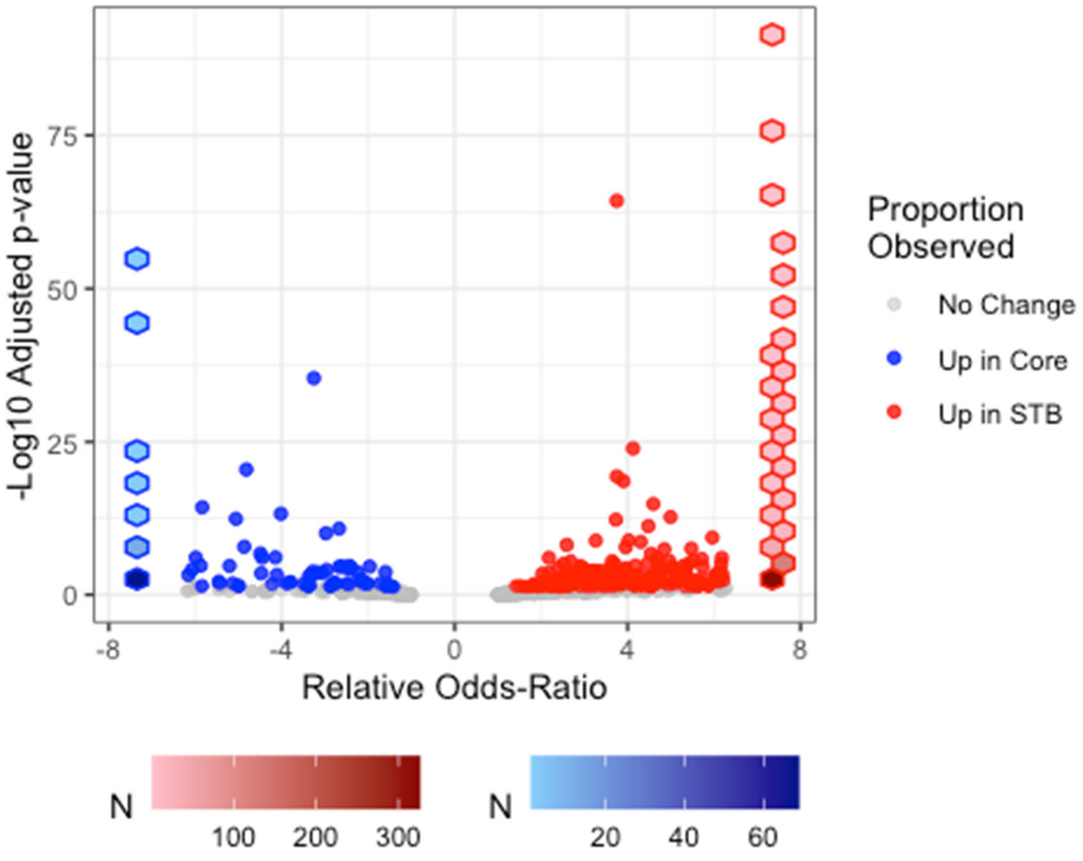

Figure 12. Volcano plot for the probability of detection-based model comparing tissue subregion mean detection probabilities for each protein. Red hexagons and blue hexagons represent proteins identified exclusively in the STB and Core, respectively. The color of each hexagon corresponds to the scale bars below the graph, which indicate the number of proteins (N). Source data are provided as a Source Data file.

Supplementary References

1. Wickham, H., et al. *dplyr: A Grammar of Data Manipulation*. 2023; Available from: https://cran.r-project.org/web/packages/dplyr/index.html.
2. Wickham, H. *Ggplot2: Elegant Graphics for Data Analysis*. 2016; Available from: https://ggplot2.tidyverse.org.
3. Degnan, D.J., et al., *pmartR 2.0: A Quality Control, Visualization, and Statistics Pipeline for Multiple Omics Datatypes.* Journal of Proteome Research, 2023.
4. Stratton, K.G., et al., *: Quality Control and Statistics for Mass Spectrometry-Based Biological Data.* Journal of Proteome Research, 2019. **18**(3): p. 1418-1425.
5. Bates, D., et al., *Fitting Linear Mixed-Effects Models Using lme4.* Journal of Statistical Software, 2015. **67**(1): p. 1-48.
6. Stoffel, M.A., S. Nakagawa, and H. Schielzeth, *partR2: partitioning R in generalized linear mixed models.* Peerj, 2021. **9**.
7. Webb-Robertson, B.J.M., et al., *Combined Statistical Analyses of Peptide Intensities and Peptide Occurrences Improves Identification of Significant Peptides from MS-Based Proteomics Data.* Journal of Proteome Research, 2010. **9**(11): p. 5748-5756.
8. Matzke, M.M., et al., *Improved quality control processing of peptide-centric LC-MS proteomics data.* Bioinformatics, 2011. **27**(20): p. 2866-2872.
9. Webb-Robertson, B.J.M., et al., *A statistical selection strategy for normalization procedures in LC-MS proteomics experiments through dataset-dependent ranking of normalization scaling factors.* Proteomics, 2011. **11**(24): p. 4736-4741.
10. Stacklies, W., et al., *pcaMethods - a bioconductor package providing PCA methods for incomplete data.* Bioinformatics, 2007. **23**(9): p. 1164-1167.
11. Benjamini, Y. and Y. Hochberg, *Controlling the False Discovery Rate - a Practical and Powerful Approach to Multiple Testing.* Journal of the Royal Statistical Society Series B-Statistical Methodology, 1995. **57**(1): p. 289-300.
