# Supplementary material for: Advanced multi-modal mass spectrometry imaging reveals functional differences of placental villous compartments at microscale resolution
Source: Nat Commun. 2025 Feb 28;16:2061. doi: 10.1038/s41467-025-57107-y (PMC11871073; doi:10.1038/s41467-025-57107-y)
Supplement: Supplementary file 2 — Reporting Summary [file 41467_2025_57107_MOESM2_ESM.pdf]

nature portfolio

Corresponding author(s): Kristin Burnum-Johnson

Last updated by author(s): Jan 20, 2025

Reporting Summary

Nature Portfolio wishes to improve the reproducibility of the work that we publish. This form provides structure for consistency and transparency in reporting. For further information on Nature Portfolio policies, see our Editorial Policies and the Editorial Policy Checklist.

Statistics

For all statistical analyses, confirm that the following items are present in the figure legend, table legend, main text, or Methods section.

n/a	Confirmed	
☐	☒	The exact sample size (*n*) for each experimental group/condition, given as a discrete number and unit of measurement
☐	☒	A statement on whether measurements were taken from distinct samples or whether the same sample was measured repeatedly
☐	☒	The statistical test(s) used AND whether they are one- or two-sided<br>*Only common tests should be described solely by name; describe more complex techniques in the Methods section.*
☒	☐	A description of all covariates tested
☐	☒	A description of any assumptions or corrections, such as tests of normality and adjustment for multiple comparisons
☐	☒	A full description of the statistical parameters including central tendency (e.g. means) or other basic estimates (e.g. regression coefficient) AND variation (e.g. standard deviation) or associated estimates of uncertainty (e.g. confidence intervals)
☐	☒	For null hypothesis testing, the test statistic (e.g. *F*, *t*, *r*) with confidence intervals, effect sizes, degrees of freedom and *P* value noted<br>*Give P values as exact values whenever suitable.*
☒	☐	For Bayesian analysis, information on the choice of priors and Markov chain Monte Carlo settings
☒	☐	For hierarchical and complex designs, identification of the appropriate level for tests and full reporting of outcomes
☐	☒	Estimates of effect sizes (e.g. Cohen's *d*, Pearson's *r*), indicating how they were calculated

Our web collection on statistics for biologists contains articles on many of the points above.

Software and code

Policy information about availability of computer code

Data collection	Metabolomic imaging: MALDI-12T-FTICR MS runs on FlexImaging (v 4.1).<br>Lipidomic imaging: UHMR Q-Exactive HF operates on Tune (v.2.12 Build 3134) operated under custom priviledge licenses. Spectroglyph EP-MALDI source runs on MALDI Injector (v.1.3.1.595).<br>Proteomics: Orbitrap Exploris 240 operates on Tune (4.2-4.2.319.22-SP3/4.2.362.36), Thermo Xcalibur software (v 4.6.67.17)
Data analysis	Metabolomic imaging: METASPACE (https://metaspace2020.eu) and SCiLS Lab (Version 2024b Core).<br>Lipidomics imaging: METASPACE (https://metaspace2020.eu), SCILS Lab Premium (v.2021c) for import of .RAW and .xml files, SCILS Lab Premium (v.2024b), and Mozaic (v2023.4.0.b3) for .imzML generation from .RAW and .xml.<br>Proteomics: MSGFPlus (v2023.01.12), and MASIC (Release 3.2.8710).<br>Statistical analysis: R (v 4.3.2)

For manuscripts utilizing custom algorithms or software that are central to the research but not yet described in published literature, software must be made available to editors and reviewers. We strongly encourage code deposition in a community repository (e.g. GitHub). See the Nature Portfolio guidelines for submitting code & software for further information.

Data

Policy information about availability of data

All manuscripts must include a data availability statement. This statement should provide the following information, where applicable:

- Accession codes, unique identifiers, or web links for publicly available datasets
- A description of any restrictions on data availability
- For clinical datasets or third party data, please ensure that the statement adheres to our policy

MALDI MSI data generated for lipidomic and metabolomic imaging can be found at:
Section 1 – lipidomic imaging
https://metaspace2020.eu/annotations?db_id=24&ds=2024-07-12_21h29m37s&fdr=0.2&row=15
Section 2 – lipidomic imaging
https://metaspace2020.eu/annotations?db_id=24&prj=0b2d571a-42e1-11ef-86c2-4b75175175b6&ds=2024-07-12_21h26m04s&fdr=0.2&page=2&row=4
Section 3 – metabolomic imaging
https://metaspace2020.eu/annotations?db_id=38&ds=2024-02-26_22h34m46s&sort=fdr_msm
Section 4 – metabolomic imaging
https://metaspace2020.eu/annotations?db_id=38&prj=0b2d571a-42e1-11ef-86c2-4b75175175b6&ds=2024-02-26_22h36m04s&row=9

The Raw proteomics, lipidomics, and metabolomics data have been deposed to Mass Spectrometry Interactive Virtual Environment (MassIVE) (https://massive.ucsd.edu) and can be accessed with dataset identifier MSV000095456 for MassIVE and PXD054289 for ProteomeXchange.

Research involving human participants, their data, or biological material

Policy information about studies with human participants or human data. See also policy information about sex, gender (identity/presentation), and sexual orientation and race, ethnicity and racism.

Reporting on sex and gender	The study was performed on placental tissue hence collected from a female individual. Sex was considered in the study design as a placenta (and fetus) can be of either sex. In this study only one placenta was collected from a female fetus (sex assigned at birth). This was stated in the methods section.
Reporting on race, ethnicity, or other socially relevant groupings	This study describes a novel methodology and utilizes tissue from a single individual. Hence data on race/ethnicity was not relevant to the study and was not collected.
Population characteristics	This study describes a novel methodology and utilizes tissue from a single individual. The subject characteristics were not relevant to the study.
Recruitment	Pregnant individuals scheduled for elective cesarean section at term are approached by trained staff to ask if they are willing to donate the delivered placenta for research studies. Tissue was collected with informed consent into a repository under a protocol approved by the Institutional Review Board of Oregon Health & Science University. The tissue was de-identified before being released to the investigators.
Ethics oversight	The study protocol was approved by the Institutional Review Board of Oregon Health & Science University

Note that full information on the approval of the study protocol must also be provided in the manuscript.

Field-specific reporting

Please select the one below that is the best fit for your research. If you are not sure, read the appropriate sections before making your selection.

☒ Life sciences ☐ Behavioural & social sciences ☐ Ecological, evolutionary & environmental sciences

For a reference copy of the document with all sections, see nature.com/documents/nr-reporting-summary-flat.pdf

Life sciences study design

All studies must disclose on these points even when the disclosure is negative.

Sample size	No sample-size calculation was performed. 14 independent biological replicates of each villous subregion (villous STB and villous core) were collected and processed.
Data exclusions	A robust Mahalanobis distance based on peptide abundance vectors (rMd-PAV) was calculated to identify potential sample outliers in the data using a p-value of 0.0001; one flagged sample was removed after being determined to be an outlier confirmed by principal component analysis (PCA).
Replication	For metabolomics imaging, 2 adjacent placenta sections were used to test the reproducibility of the metabolomics data.<br>For lipidomics imaging, 2 adjacent placenta sections were used to test the reproducibility of the lipidomic data.<br>For proteomic profiling, 3 sections in close proximity to MALDI-imaged placental sections were obtained. Villous subregions that were previously mapped by MALDI-MSI analyses were excised and collected. 14 villous STB replicates and 14 villous core replicates were collected

	from 3 adjacent sections and independently processed to demonstrate the reproducibility in proteomics data.
Randomization	Villous STB and villous core replicates collected from a single placenta tissue section were considered as an experimental group where samples were processed and analyzed using randomized orders.
Blinding	Investigators were not blinded to group allocation during data collection or analysis

Reporting for specific materials, systems and methods

We require information from authors about some types of materials, experimental systems and methods used in many studies. Here, indicate whether each material, system or method listed is relevant to your study. If you are not sure if a list item applies to your research, read the appropriate section before selecting a response.

Materials & experimental systems

n/a	Involved in the study
☒	☐ Antibodies
☒	☐ Eukaryotic cell lines
☒	☐ Palaeontology and archaeology
☒	☐ Animals and other organisms
☒	☐ Clinical data
☒	☐ Dual use research of concern
☒	☐ Plants

Methods

n/a	Involved in the study
☒	☐ ChIP-seq
☒	☐ Flow cytometry
☒	☐ MRI-based neuroimaging

Plants

Seed stocks	N/A
Novel plant genotypes	N/A
Authentication	N/A
